# Raptor downregulation rescues neuronal phenotypes in mouse models of Tuberous Sclerosis Complex

**DOI:** 10.1038/s41467-022-31961-6

**Published:** 2022-08-09

**Authors:** Vasiliki Karalis, Franklin Caval-Holme, Helen S. Bateup

**Affiliations:** 1 Department of Molecular and Cell Biology, University of California, Berkeley, Berkeley, CA 94720 USA; 2 Helen Wills Neuroscience Institute, University of California, Berkeley, Berkeley, CA 94720 USA; 3 Chan Zuckerberg Biohub, San Francisco, CA 94158 USA

**Keywords:** Developmental disorders, Epilepsy

## Abstract

Tuberous Sclerosis Complex (TSC) is a neurodevelopmental disorder caused by mutations in the *TSC1* or *TSC2* genes, which encode proteins that negatively regulate mTOR complex 1 (mTORC1) signaling. Current treatment strategies focus on mTOR inhibition with rapamycin and its derivatives. While effective at improving some aspects of TSC, chronic rapamycin inhibits both mTORC1 and mTORC2 and is associated with systemic side-effects. It is currently unknown which mTOR complex is most relevant for TSC-related brain phenotypes. Here we used genetic strategies to selectively reduce neuronal mTORC1 or mTORC2 activity in mouse models of TSC. We find that reduction of the mTORC1 component Raptor, but not the mTORC2 component Rictor, rebalanced mTOR signaling in Tsc1 knock-out neurons. Raptor reduction was sufficient to improve several TSC-related phenotypes including neuronal hypertrophy, macrocephaly, impaired myelination, network hyperactivity, and premature mortality. Raptor downregulation represents a promising potential therapeutic intervention for the neurological manifestations of TSC.

## Abstract (web summary)

Karalis et al show that genetic reduction of the mTORC1 component Raptor improves multiple phenotypes in mouse models of TSC. Their findings suggest that Raptor could be a potential therapeutic target for treating the neurological aspects of TSC.

## Introduction

Tuberous Sclerosis Complex (TSC) is a neurodevelopmental disorder resulting in benign tumors in multiple organs and focal cortical malformations called tubers^1^. Some of the most significant problems associated with TSC are the neurological and psychiatric aspects^2^. Approximately 85% of TSC patients develop epilepsy, which often begins in infancy and becomes intractable in two-thirds of cases^3,4^. TSC is also associated with varying degrees of intellectual disability, cognitive impairments, and behavioral conditions including autism spectrum disorder and attention deficit hyperactivity disorder^5^.

Current treatment strategies for TSC include inhibitors of mTOR signaling called rapalogs, which are analogs of the naturally occurring macrolide rapamycin^6,7^. Rapamycin has been successful in treating neuropsychiatric phenotypes in rodent models of TSC, especially when treatment is started early in development^8,9^. In the clinic, rapalogs are moderately effective at treating seizures and subependymal giant cell astrocytomas (SEGAs), which are benign brain tumors that affect about 5–15% of TSC patients^10–14^. However, symptoms can return after treatment cessation^10^ and chronic rapalog use is associated with significant side effects including immunosuppression and systemic metabolic changes such as insulin resistance^15^. In addition, several recent clinical trials have reported that rapalogs did not significantly improve the cognitive and psychiatric aspects of TSC^16,17^. As a result, additional therapeutic interventions for TSC are needed.

TSC is caused by loss-of-function mutations in either the *TSC1* or *TSC2* genes^18,19^. At the biochemical level, the protein products of *TSC1* and *TSC2* form a complex that inhibits Rheb, a small GTPase that activates mTOR complex 1 (mTORC1)^20^. The TSC2 protein harbors the GTPase activating protein (GAP) domain, while TSC1 is required for complex stability^21^. mTORC1 is composed of several proteins including mTOR and the obligatory component Regulatory-associated protein of mTOR (Raptor)^22–24^. mTORC1 is a kinase that phosphorylates several targets including P70S6 kinase (P70S6K) and the 4E binding proteins (4E-BPs), which are involved in translational control^25–27^. P70S6K in turn phosphorylates ribosomal protein S6, a canonical read-out of mTORC1 activity^28^. The activity of mTORC1 is regulated by various intra- and extracellular signals including growth factors, insulin, nutrients, and neuronal activity^29,30^. When active, mTORC1 promotes anabolic cellular processes such as protein, lipid, and nucleotide synthesis and suppresses catabolic processes, including autophagy^30,31^. In the absence of regulation by the TSC1/2 complex, mTORC1 is constitutively active^20^.

Rapamycin is thought to suppress mTORC1 signaling by inducing a trimeric complex between itself, mTOR and FK506-binding protein 12 (FKBP12)^22^. The binding of rapamycin-FKBP12 on mTORC1 occludes access of some substrates to the catalytic site of the mTOR kinase^22^. While acute administration of rapamycin reduces mTORC1 signaling, chronic treatment also suppresses the activity of a second mTOR complex, mTORC2, potentially by preventing *de novo* mTORC2 assembly^32^. mTORC2 shares several protein components with mTORC1 but instead of Raptor, mTORC2 contains Rapamycin-insensitive companion of mTOR (Rictor) as an essential component^31,33,34^. mTORC2 has been reported to control aspects of cytoskeletal organization, synaptic transmission, cell survival, and metabolism^30,35–38^, although its functions are less well understood. The most well-characterized phosphorylation target of mTORC2 is Ser473 of Akt^39^. While mTORC1 and mTORC2 have conventionally been thought to have distinct upstream regulators and targets, studies in various systems have revealed potential points of crosstalk between the two complexes^35,40,41^.

TSC-related phenotypes are canonically attributed to mTORC1 hyperactivity; however, recent studies have raised the possibility that mTORC2 may also be involved. In particular, a study in mice with forebrain-specific disruption of Pten, a negative regulator of mTORC1 that is upstream of the Tsc1/2 complex, showed that downregulation of mTORC2, but not mTORC1, could prevent behavioral abnormalities, seizures, and premature mortality^42^. Moreover, it was shown that mTORC2, but not mTORC1, is required for hippocampal mGluR-dependent long-term depression (LTD)^43^, a form of synaptic plasticity that is altered in mouse models of TSC^44–47^. Therefore, a careful investigation of the relationships between Tsc1/2, mTORC1, and mTORC2 in the context of brain development and function is needed to design the most effective therapeutic strategy for TSC.

Here we used in vitro and in vivo mouse models of TSC to investigate whether manipulation of mTORC1 or mTORC2 signaling via genetic reduction of Raptor or Rictor, respectively, could ameliorate TSC-related brain phenotypes. We find that reduction, but not complete elimination, of Raptor rebalances both mTORC1 and mTORC2 signaling in the context of Tsc1 loss. We show that heterozygous deletion of *Rptor* in conditional *Tsc1* knock-out mice (Tsc1-cKO) improves several brain phenotypes including neuronal hypertrophy, neural network hyperactivity, impaired myelination, altered cortical and hippocampal architecture, and premature mortality. By contrast, heterozygous or homozygous loss of *Rictor* does not significantly improve TSC-related neuronal phenotypes. Finally, we demonstrate that postnatal Raptor downregulation rescues neuronal hypertrophy and myelination deficits in Tsc1-cKO mice and extends survival. Our results establish Raptor as a potential target for treating the neurological presentations of TSC.

## Results

### Chronic rapamycin treatment suppresses mTORC1 and mTORC2 signaling in Tsc1-cKO hippocampal cultures

**Fig. 1:**
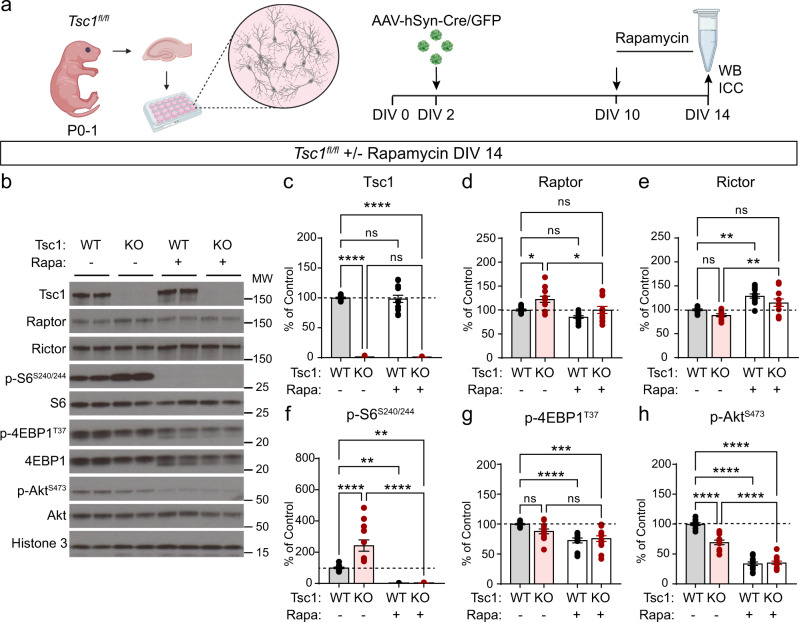
Chronic rapamycin suppresses mTORC1 and mTORC2 signaling in cultured Tsc1-cKO neurons. **a** Schematic of the experiment, created with BioRender.com. **b** Representative western blots (WB) from *Tsc1*^*fl/fl*^ cultures with (+) or without (−) four-day rapamycin (Rapa) treatment. MW indicates molecular weight. WT=*Tsc1*^*fl/fl*^ + AAV-GFP; KO=*Tsc1*^*fl/fl*^ + AAV-Cre-GFP. Two independent samples per genotype are shown. This experiment was replicated 5 times. **c**–**h** Bar graphs display WB quantification (mean ± SEM) for the indicated proteins, expressed as a percentage of Control (WT) levels. Phospho-proteins were normalized to their respective total proteins. Dots represent data from individual culture wells. WT *n* = 11, KO *n* = 12, WT+Rapa *n* = 13 and KO+Rapa *n* = 13 culture wells from 5 independent culture preps; 2 mice per culture. ns = non-significant. Dashed lines at 100% indicate Control levels. All statistical tests were two-sided and *P* values were corrected for multiple comparisons. **c** Tsc1, Kruskal–Wallis, *p* < 0.0001; WT vs KO, ****p < 0.0001; WT vs WT+Rapa, *p* > 0.9999; WT vs KO+Rapa, ****p < 0.0001; KO vs KO+Rapa, *p* > 0.9999; Dunn’s multiple comparisons tests. **d** Raptor, One-way ANOVA, *p* = 0.0001, *F*(3, 45) = 8.495; WT vs KO, *p = 0.0221; WT vs WT+Rapa, *p* = 0.2187; WT vs KO+Rapa, *p* > 0.9999; KO vs KO+Rapa, *p = 0.0161; Sidak’s multiple comparisons tests. **e** Rictor, One-way ANOVA, *p* < 0.0001, *F*(3, 45) = 12.09; WT vs KO *p* = 0.4323; WT vs WT+Rapa, **p = 0.0010; WT vs KO+Rapa, *p* = 0.1773; KO vs KO+Rapa, **p = 0.0025, Sidak’s multiple comparisons tests. **f** p-S6 Ser240/244, One-way ANOVA, *p* < 0.0001, *F*(3, 45) = 41.21; WT vs KO, ****p < 0.0001; WT vs WT+Rapa, **p = 0.0014; WT vs KO+Rapa, **p = 0.0015; KO vs KO+Rapa, ****p < 0.0001; Sidak’s multiple comparisons tests. **g** p-4EBP1 T37, One-way ANOVA, *p* < 0.0001, *F*(3, 45) = 9.997; WT vs KO, *p* = 0.1355; WT vs WT+Rapa, ****p < 0.0001; WT vs KO+Rapa, ***p = 0.0003; KO vs KO+Rapa, *p* = 0.1218; Sidak’s multiple comparisons tests. **h** p-Akt Ser473, One-way ANOVA, *p* < 0.0001, *F*(3, 45) = 107.6; WT vs KO, ****p < 0.0001; WT vs WT+Rapa, ****p < 0.0001; WT vs KO+Rapa, ****p < 0.0001; KO vs KO+Rapa, ****p < 0.0001; Sidak’s multiple comparisons tests. See also Supplementary Figs. 1, 2. Source data are provided as a Source Data file.

To investigate how loss of Tsc1 affects mTORC1 and mTORC2 signaling, we generated primary hippocampal cultures from *Tsc1*^*fl/fl*^ mice^48^ and treated them with adeno-associated virus (AAV) expressing GFP (control) or Cre (Tsc1-cKO) at two days in vitro (DIV 2) (Fig. 1a and Supplementary Fig. 1a, b). To assess mTORC1 signaling we quantified Raptor protein levels and two canonical downstream phosphorylation targets, ribosomal protein S6 (p-S6 Ser240/244) and 4E-BP1 (p-4E-BP1 Thr37). To measure mTORC2 signaling we quantified Rictor protein levels and Akt phosphorylation at Ser 473 (p-Akt Ser473). Changes in phosphorylation state were assessed by normalizing phospho-proteins to their respective total proteins. We found that on DIV 14, Tsc1-cKO cultures had complete loss of Tsc1 and a small increase in Raptor protein (Fig. 1b–d). No significant changes in Rictor protein were observed (Fig. 1e). Phosphorylation of S6 was increased in Tsc1-cKO cultures, as expected (Fig. 1f). However, we did not observe strong upregulation in p-4E-BP1 in this system (Fig. 1g), which may be due to the timing of Tsc1 loss, as discussed further below (see also additional discussion in the Methods). In terms of mTORC2, we found that *Tsc1* deletion decreased Akt phosphorylation at Ser473 (Fig. 1h), indicating reduced mTORC2 activity. Together these data show that in hippocampal cultures, loss of *Tsc1* has opposing effects on the two mTOR complexes: it increases mTORC1 signaling, particularly the S6 branch, and decreases mTORC2 activity.

We next examined the effects of rapamycin on mTOR signaling to provide a benchmark for comparing the effects of genetic manipulation of the two mTOR complexes. Four-day treatment with 50 nM rapamycin had no effect on Tsc1 levels but slightly reduced Raptor and increased Rictor levels in Tsc1-cKO cultures (Fig. 1c–e). We observed the expected complete loss of p-P70S6K1, p-S6 and partial reduction of p-4E-BP1, indicative of suppressed mTORC1 signaling (Fig. 1f, g and Supplementary Fig. 2a–c). Notably, we observed a strong reduction of p-Akt in both control and Tsc1-cKO neurons treated chronically with rapamycin, which was greater than with Tsc1 loss alone (Fig. 1h). Together, these data demonstrate that rapamycin treatment does not restore balanced mTOR signaling in Tsc1-cKO hippocampal cultures but rather strongly suppresses the S6 branch of mTORC1, partially decreases p-4E-B1, and further reduces mTORC2-dependent Akt phosphorylation.

### Downregulation of Raptor, but not Rictor, improves mTOR signaling abnormalities in Tsc1-cKO neurons

**Fig. 2:**
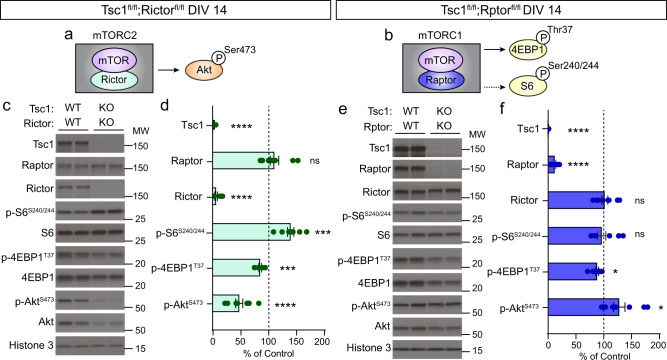
Genetic reduction of Raptor ameliorates mTOR signaling abnormalities in Tsc1-cKO neurons. **a** Simplified schematic of mTORC2 showing mTOR and its obligatory binding partner Rictor. mTORC2 phosphorylates Ser473 on Akt. **b** Simplified schematic of mTORC1 showing mTOR and its obligatory binding partner Raptor. mTORC1 phosphorylates 4EBP1 (Thr37) and P70S6K, which in turn phosphorylates S6 at Ser240/244 (represented by the dashed line). **c** Representative western blots (WB) from *Tsc1*^*fl/fl*^;*Rictor*^*fl/fl*^ hippocampal cultures treated with AAV-GFP (WT;WT) or AAV-Cre-GFP (KO;KO) and harvested on DIV 14. MW indicates molecular weight. Two independent samples per genotype are shown. This experiment was replicated three times. **d** Bar graphs display WB quantification (mean ± SEM) for the indicated proteins, expressed as a percentage of Control (WT) levels. *n* = 9 culture wells from 3 independent cultures per genotype; 2 mice per culture. Tsc1 ****p < 0.0001; Raptor *p* = 0.2380; Rictor ****p < 0.0001; p-S6 Ser240/244 ***p = 0.0007; p-4EBP1 T37 ***p = 0.0002; p-Akt Ser473 ****p < 0.0001; two-sided Welch’s *t*-tests. **e** Representative WB of lysates collected from *Tsc1*^*fl/fl*^;*Rptor*^*fl/fl*^ hippocampal cultures treated with AAV-GFP (WT;WT) or AAV-Cre-GFP (KO;KO) and harvested on DIV 14. Two independent samples per genotype are shown. This experiment was replicated three times. **f** Bar graphs display WB quantification (mean ± SEM) for the indicated proteins, expressed as a percentage of Control (WT) levels. *n* = 9 culture wells from 3 independent cultures per genotype; 2 mice per culture. Tsc1 ****p < 0.0001; Raptor ****p < 0.0001; Rictor *p* = 0.8199; p-S6 Ser240/244 *p* = 0.6616; p-4EBP1 T37 *p = 0.0146; p-Akt Ser473 *p = 0.0283; two-sided Welch’s *t* tests. For panels (**d**) and (**f**), phospho-proteins were normalized to their respective total proteins and dots represent data from individual culture wells. Dashed lines at 100% indicate Control levels. ns = non-significant. See also Supplementary Figs. 1–3. Source data are provided as a Source Data file.

To investigate whether genetic reduction of mTORC1 or mTORC2 could improve signaling abnormalities in Tsc1-cKO neurons, we crossed *Tsc1*^*fl/fl*^ mice to mice with either floxed *Rictor*^49,50^ or *Rptor*^51^ alleles for simultaneous Cre-dependent deletion of *Tsc1* and *Rictor* or *Tsc1* and *Rptor*, respectively (Fig. 2a, b and Supplementary Fig. 1c, d). We found that mTORC1 hyperactivity persisted in Tsc1-cKO cultures with deletion of *Rictor*, evidenced by significantly increased levels of p-S6 in Tsc1-cKO;Rictor-cKO cultures compared to controls (Fig. 2c, d). p-Akt-473 was strongly reduced in Tsc1-cKO;Rictor-cKO neurons (Fig. 2c, d). Thus, the mTOR signaling perturbations in Tsc1-cKO hippocampal cultures were not corrected by genetic reduction of Rictor.

We next examined how the reduction of Raptor affected mTOR signaling in Tsc1-cKO neurons. Homozygous deletion of *Rptor* effectively normalized p-S6 and p-P70S6K1 levels in Tsc1-cKO neurons harvested on DIV 14 (Fig. 2e, f and Supplementary Fig. 2f, g). Deletion of *Rptor* also reversed the reduction of p-Akt caused by Tsc1 loss, suggesting a boosting of mTORC2 activity (Fig. 2e, f). The enhanced Akt phosphorylation resulting from Raptor loss may be due to relief of mTORC1-dependent negative feedback, either on mTORC2 itself or upstream regulators^41^. These data indicate that Raptor downregulation could be an effective strategy for rebalancing mTOR signaling abnormalities due to loss of Tsc1.

**Fig. 3:**
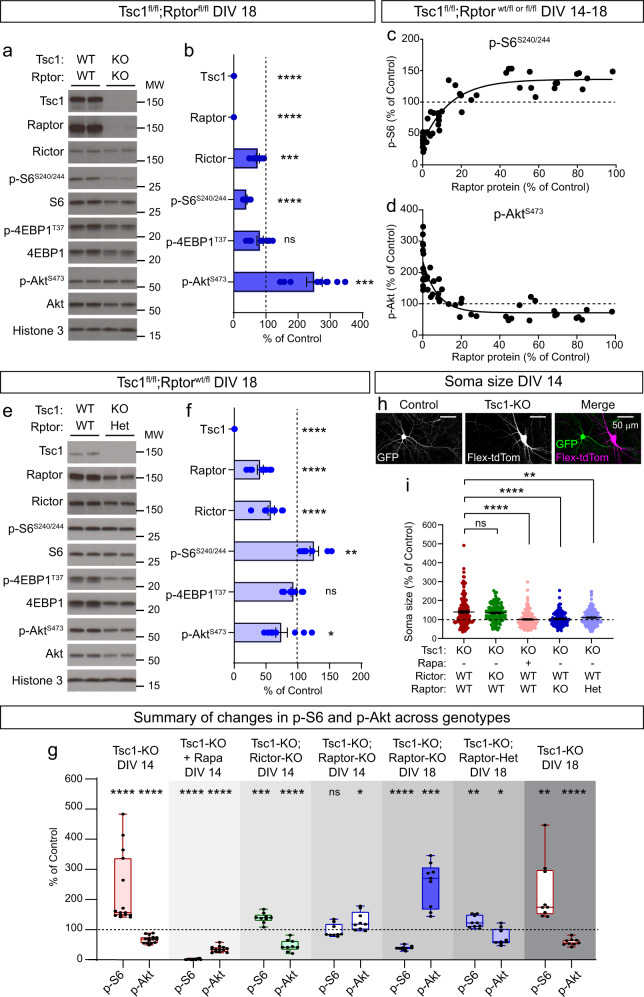
Partial reduction of Raptor normalizes mTOR signaling in Tsc1-cKO cultures. **a** Representative western blots (WB) from *Tsc1*^*fl/fl*^;*Rptor* ^*fl/fl*^ hippocampal cultures treated with AAV-GFP (WT;WT) or AAV-Cre-GFP (KO;KO) and harvested on DIV 18. MW indicates molecular weight. Two independent samples per genotype are shown. This experiment was replicated three times. **b** Bar graphs display WB quantification (mean ± SEM) for the indicated proteins, expressed as a percentage of Control (WT) levels. **c**, **d** Correlation of Raptor protein levels to p-S6 Ser240/244 (**c**) or p-Akt Ser473 (**d**) within each culture, expressed as a percentage of their respective Control. **e** Representative WBs from *Tsc1*^*fl/fl*^;*Rptor*^*wt/fl*^ hippocampal cultures treated with AAV-GFP (WT;WT) or AAV-Cre-GFP (KO;Het) and harvested on DIV 18. Two independent samples per genotype are shown. This experiment was replicated three times. **f** Bar graphs display WB quantification (mean ± SEM) for the indicated proteins, expressed as a percentage of Control (WT) levels. **g** Box-and-whisker plot summary of p-S6 Ser240/244 and p-Akt Ser473 WB results for the indicated conditions, expressed as a percentage of their respective controls. Within each box, lines represent the median, boxes extend from the 25th to the 75th percentile, and whiskers represent minimum to maximum values. Data are replotted from Figs. 1–3, except for Tsc1-KO DIV 18, which is shown here for comparison. **h** Representative images showing a GFP-expressing control neuron (left panel) and an AAV-mCherry-Cre + AAV-Flex-tdTomato transduced Tsc1-KO neuron (middle panel). Right panel shows the merged image. Scale bars = 50 μm. **i** Scatter dot plot of soma area of cultured hippocampal neurons of the indicated genotypes, expressed as a percentage of Control (WT) neurons from the same culture. Dots represent individual neurons. Black lines indicate mean ± SEM. Dashed lines at 100% indicate control levels. For panels (**b**, **c**, **d**, **f**, **g**), phospho-proteins were normalized to their respective total proteins. For panels (**b**, **f**, **g**), dots represent data from individual culture wells. See also Supplementary Figs. 3, 4. Statistics for this figure are reported in Supplementary Data 1. Source data are provided as a Source Data file.

During this experiment, we noted that low levels of Raptor protein remained in the cultures on DIV 14, which could have accounted for the incomplete suppression of mTORC1 signaling compared to rapamycin treatment (see Fig. 1). To investigate this further, we added AAV-Cre to Tsc1-cKO;Rptor-cKO cultures on DIV 2 and harvested the cells at different time points (Supplementary Fig. 3a–g). We found that Raptor levels decreased over time such that by DIV 18 (16 days post-Cre) there was less than 1% of Raptor protein remaining (Supplementary Fig. 3c). This contrasts with Tsc1 protein, which was already undetectable at DIV 14 (Supplementary Fig. 3b). The slow turnover of Raptor may reflect high stability of Raptor protein when bound within mTORC1. We therefore harvested Tsc1-cKO;Raptor-cKO cultures on day 18 to assess the consequences of more complete loss of Raptor on mTORC1 and mTORC2 signaling. At this time point, we found that concomitant deletion of *Tsc1* and *Rptor* strongly reduced p-S6 and increased p-Akt levels (Fig. 3a, b and Supplementary Fig. 3e, g). We did not observe a consistent reduction in 4E-BP1 phosphorylation (Fig. 3a, b and Supplementary Fig. 3f), suggesting that, as with rapamycin^52^, mTORC1 targets are differentially susceptible to Raptor downregulation.

Our findings suggest that relatively low levels of Raptor protein may be sufficient to sustain mTORC1 signaling in the absence of negative regulation by the Tsc1/2 complex.

To further evaluate the relationships between Raptor, mTORC1, and mTORC2, we analyzed results from cultures harvested at different time points, which had complete loss of Tsc1 protein and variable loss of Raptor protein (as in Supplementary Fig. 3b, c). We plotted the correlation of Raptor protein levels with p-S6 and p-Akt, expressed as a percentage of control cultures from the same genotype and time point. We observed a non-linear relationship whereby p-S6 remained elevated in Tsc1-cKO cultures even when Raptor protein levels were reduced by up to 80% (Fig. 3c). Only when Raptor levels dropped below 20% of control did we observe a reduction in p-S6 levels. The inverse relationship was observed for p-Akt, which was consistently reduced with Tsc1 loss and increased sharply as Raptor levels fell below 20% (Fig. 3d). Similar relationships were observed in hippocampal cultures with downregulation of Raptor alone, which were wild-type for *Tsc1* (Supplementary Fig. 3h–j). In this case, mild mTORC1 suppression was observed with 10–80% loss of Raptor protein; however, when Raptor levels were less than ~10% of control, p-S6 was strongly suppressed and p-Akt was robustly increased. Together, these analyses reveal a non-linear relationship between Raptor and mTORC1 signaling and suggest that relatively low Raptor protein levels can maintain mTORC1 activity in neuronal cultures.

The above results indicate that partial reduction of Raptor protein may be an effective strategy to normalize both mTORC1 and mTORC2 signaling in Tsc1-cKO neurons. This would avoid the strong suppression of both complexes observed with rapamycin treatment and the inhibition of mTORC1 and increase in mTORC2 activity observed with complete Raptor loss. To test this, we assessed mTOR signaling in cultures from *Tsc1*^fl/fl^;*Rptor*^*wt/fl*^ mice, which had loss of one copy of *Rptor* in the Tsc1-cKO background. We harvested Tsc1-cKO;Raptor-cHet cultures on DIV 18 and found that p-S6 and p-Akt levels exhibited an intermediate phenotype between Tsc1-cKO and Tsc1-cKO;Raptor-cKO cultures (Fig. 3e, f). These data show that loss of one copy of *Rptor* can significantly ameliorate, although not completely prevent, the mTORC1 and mTORC2 signaling abnormalities associated with Tsc1 loss. Figure 3g provides a summary of the effects of rapamycin, Rictor, and Raptor manipulations on p-S6 and p-Akt levels in Tsc1-cKO cultures.

### Reduction of Raptor prevents hypertrophy of Tsc1-cKO neurons

Cellular hypertrophy is a well-established consequence of mTORC1 hyperactivity^31^. To test whether genetic reduction of Raptor could improve somatic hypertrophy, we sparsely labeled cultured neurons with florescent proteins and measured soma size. In this experiment, control cells expressed GFP and mutant cells within the same culture expressed nuclear Cre-mCherry and Cre-dependent cytoplasmic tdTomato (Fig. 3h). We observed the expected increase in soma size of Tsc1-cKO neurons compared to controls, which was reversed by four-day treatment with rapamycin (Fig. 3i and Supplementary Fig. 4a–f). Homozygous deletion of *Rictor* did not prevent cellular hypertrophy as Tsc1-cKO;Rictor-cKO neurons exhibited significantly increased soma size compared to controls (Fig. 3i and Supplementary Fig. 4g–i). By contrast, homozygous or heterozygous deletion of *Rptor* reduced the size of Tsc1-cKO neurons, effectively normalizing soma area to control levels (Fig. 3i and Supplementary Fig. 4j–o). Together, these results demonstrate that genetic reduction of Raptor is sufficient to prevent hypertrophy of Tsc1-cKO neurons in vitro.

### Genetic reduction of Raptor, but not Rictor, extends the lifespan of Tsc1-cKO mice

**Fig. 4:**
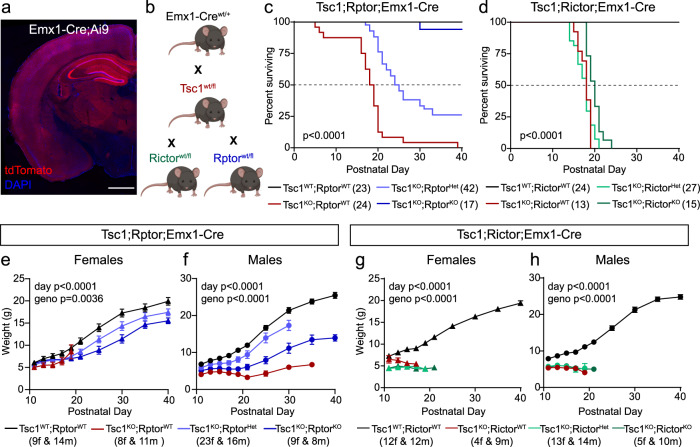
Reduction of *Rptor* prolongs the lifespan of Tsc1-cKO mice. **a** Representative image showing one hemisphere of a coronal section from an *Emx1-Cre*^+^;*Ai9*^+^ mouse. The Cre-dependent tdTomato reporter is in red. DAPI labels cell nuclei (blue). This experiment was performed once. Scale bar = 1 mm. **b** Schematic of the crosses used to generate experimental mice. *Emx1-Cre*^*wt/+*^ mice were crossed with *Tsc1*^*wtl/fl*^ mice. This line was then bred to either *Rictor*^*wt/fl*^ or *Rptor*^*wt/fl*^ mice. Created with BioRender.com. **c**, **d** Survival analysis of *Tsc1;Rptor;Emx1-Cre* (**c**) and *Tsc1;Rictor;Emx1-Cre* (**d**) mice of the indicated genotypes. The starting number of mice for each genotype is indicated in parentheses. Dashed lines indicate 50% of the population surviving. *P* values from Log-rank Mantel–Cox tests are shown. **e**–**h** Mean ± SEM body weight in grams measured from postnatal day 11–40 for mice of the indicated sex and genotype. The starting number of mice for each genotype and sex is indicated in parentheses. f = females, m = males. Mixed-effect model (REML) with Geisser-Greenhouse correction statistics: *Tsc1;Rptor;Emx1-Cre* females (**e**), day *p* < 0.0001, *F*(1.447, 51.30) = 398.9; geno *p* = 0.0036, *F*(3, 45) = 5.192. *Tsc1;Rptor;Emx1-Cre* males (**f**), day p < 0.0001, *F*(2.287, 79.03) = 197.4; geno *p* < 0.0001, *F*(3, 45) = 26.85. *Tsc1;Rictor;Emx1-Cre* females (**g**), day *p* < 0.0001, *F*(4.257, 69.52) = 221.1; geno *p* < 0.0001, *F*(3, 30) = 53.74. *Tsc1;Rictor;Emx1-Cre* males (**h**), day *p* < 0.0001, *F*(2.389, 48.04) = 370.4; geno *p* < 0.0001, *F*(3, 41) = 28.86. Multiple comparisons testing was not performed. See also Supplementary Figs. 5, 9 and Supplementary Tables 1, 2. Source data are provided as a Source Data file.

To test whether Raptor reduction could improve TSC-related phenotypes in vivo, we used the *Emx1*^IRES*Cre*^ (Emx1-Cre)^53^ mouse line to induce *Tsc1* deletion around embryonic day (E) 9.5, in excitatory neurons and glial cells of the cortex and hippocampus (Fig. 4a). Deletion of *Tsc1* in Emx1-expressing cells leads to a severe phenotype characterized by poor development, seizures, and premature mortality in the first few weeks of life^54,55^. To test whether genetic reduction of mTORC1 or mTORC2 could prevent these phenotypes, we generated mice with heterozygous or homozygous deletion of *Rptor* or *Rictor* in the *Tsc1-cKO;Emx1-Cre* background (Fig. 4b). We monitored the survival and body weight of mice born from these crosses for the first 40 postnatal days. As expected, *Tsc1*^*fl/fl*^;*Emx1-Cre*^*+*^ (Tsc1-cKO) mice exhibited premature mortality with a median survival of 18.5 days (Fig. 4c, d and Supplementary Table 1). Tsc1-cKO mice of both sexes also had reduced body weight, although the timing of death was not correlated with weight (Fig. 4e–h, Supplementary Fig. 5a, and Supplementary Table 2).

Loss of Raptor extended the lifespan of Tsc1-cKO mice in a gene-dose-dependent manner, with homozygous *Rptor* deletion resulting in near normal survival (16/17 *Tsc1*^*fl/fl*^;*Rptor;*^*fl/fl*^*Emx1-Cre*^*+*^ animals survived until at least P40, Fig. 4c and Supplementary Table 1). Heterozygous deletion of *Rptor* led to a significant shift in the median survival of Tsc1-cKO mice from 18.5 to 24.5 postnatal days and 26% of the Tsc1-cKO;Raptor-cHet mice survived past P40 (Fig. 4c). While Tsc1-cKO;Raptor-cHet males and females had identical median survival, the length of lifespan extension was sex-dependent. After P26, the surviving female population stabilized with 40% of Tsc1-cKO;Raptor-cHet females surviving past P40 (Supplementary Fig. 5b and Supplementary Table 1). By contrast, the Tsc1-cKO;Raptor-cHet male population steadily declined and only 5% of mice survived past P40 (Supplementary Fig. 5b and Supplementary Table 1). Notably, while Tsc1-cKO;Raptor-cKO mice regularly survived past P40, they exhibited significantly decreased body weight (Fig. 4e, f and Supplementary Table 2). Similar body weight changes were observed with homozygous loss of *Rptor* in mice WT for *Tsc1* (Supplementary Table 2). Compared to WT mice, Tsc1-cKO;Raptor-cHet mice had reduced body weight but were significantly larger than Tsc1-cKO;Raptor-WT mice, suggesting improved physical development (Fig. 4e, f and Supplementary Table 2). The body weight differences between mice of different genotypes generally persisted among surviving mice at P150 (Supplementary Fig. 5c).

A study using *Pten*^*fl/fl*^;*CamKII*-*Cre*^*+*^ mice (Pten-cKO) showed that concurrent deletion of *Pten* and *Rictor* significantly shifted median survival from *P*~50 to ~110 days; however, no mice survived past P130. Surprisingly, homozygous *Rptor* deletion did not significantly affect the median survival age of Pten-cKO mice^42^. We generated *Tsc1;Rictor;Emx1-Cre* mice and found that heterozygous loss of *Rictor* did not affect the survival of Tsc1-cKO mice and homozygous loss of *Rictor* only slightly shifted the median survival from P18 to P20, with no Tsc1-cKO;Rictor-cKO animals surviving past P24 (Fig. 4d and Supplementary Table 1). It was previously shown that *Rictor*^*fl/fl*^*;Emx1-Cre*^*+*^ animals exhibited reduced body weight^56^. Consistent with this, we observed decreased body weight in mice with homozygous deletion of *Rictor*, independent of *Tsc1* genotype (Fig. 4g, h and Supplementary Table 2). Together, these findings indicate that premature mortality due to *Tsc1* deletion in the forebrain can be prevented by loss of Raptor, but not Rictor.

### Genetic downregulation of Raptor improves multiple TSC-related brain phenotypes

**Fig. 5:**
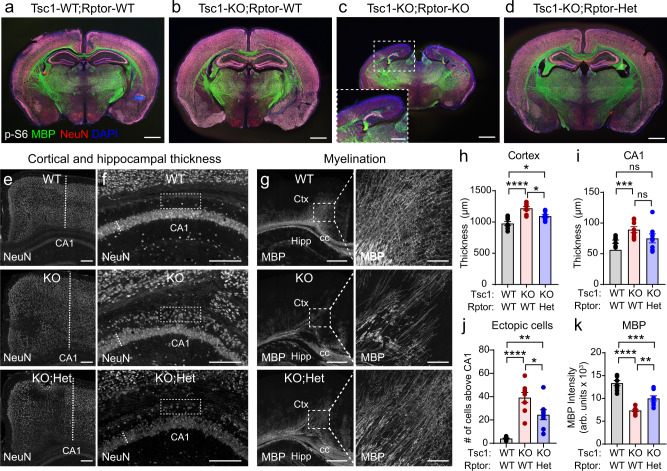
Removal of one copy of *Rptor* improves forebrain development in Tsc1-cKO mice. **a**–**d** Representative coronal brain sections from mice of the indicated genotypes. Immunostaining for p-S6 Ser240/244 is in gray, myelin basic protein (MBP) is in green, NeuN is in red and DAPI-labeled nuclei are in blue. Scale bars = 1 mm. In panel (**c**), inset shows zoomed-in image of the cortex and hippocampal region (scale bar = 500 μm). This experiment was replicated 8 times per genotype. **e** Representative images of the cortex with NeuN immunostaining. Dashed lines denote the measurement of cortical thickness. WT = Tsc1-WT;Rptor-WT, KO = Tsc1-KO;Rptor-WT, KO;Het = Tsc1-KO;Rptor-Het. Scale bars = 500 μm. **f** Representative images of the hippocampal CA1 region with NeuN immunostaining. Dashed lines denote the measurement of CA1 thickness. Boxed regions highlight the area above the CA1 that contains ectopic p-S6 expressing neurons in Tsc1-KO;Rptor-WT mice (quantified in panel (**j**)). Scale bars = 250 μm. **g** Representative images of MBP immunostaining in the cortex (Ctx) & dorsal hippocampus (Hipp). cc=corpus callosum. Scale bars = 500 μm. Right panels show higher magnification of the boxed regions in the cortex. Scale bars = 100 μm. **h** Mean ± SEM cortical thickness. One-way ANOVA, *p* < 0.0001, *F*(2, 21) = 18.40; WT vs KO, ****p < 0.0001; WT vs KO;Het, *p = 0.0226; KO vs KO;Het, *p = 0.0157; Sidak’s multiple comparisons tests. **i** Mean ± SEM CA1 thickness. One-way ANOVA, *p* = 0.0010, *F*(2, 21) = 9.703; WT vs KO, ***p = 0.0008; WT vs KO;Het, *p* = 0.0628; KO vs KO;Het, *p* = 0.1968; Sidak’s multiple comparisons tests. ns non-significant. **j** Mean ± SEM number of p-S6 positive neurons above the CA1 (boxed regions in panel (**f**)). One-way ANOVA, *p* < 0.0001, *F*(2, 21) = 24.77; WT vs KO, ****p < 0.0001; WT vs KO;Het, **p = 0.0016; KO vs KO;Het, *p = 0.0237; Sidak’s multiple comparisons tests. **k** Mean ± SEM bulk MBP fluorescence intensity (from the boxed cortical regions in panel (**g)**). One-way ANOVA, *p* < 0.0001, *F*(2, 21) = 39.19; WT vs KO, ****p < 0.0001; WT vs KO;Het, ***p = 0.0002; KO vs KO;Het, **p = 0.0025; Sidak’s multiple comparisons tests. For panels (**h**–**k**), dots represent values from individual mice, *n* = 8 mice per genotype. Two-sided statistical tests were performed and *P* values were corrected for multiple comparisons. See also Supplementary Figs. 5, 6 and Supplementary Table 3. Source data are provided as a Source Data file.

Given that downregulation of Raptor extended the lifespan of Tsc1-cKO mice in a gene-dose-dependent manner, we next examined whether TSC-related brain phenotypes could be improved. We explored a battery of common phenotypes in mouse models of TSC including macrocephaly, hypertrophic neurons, impaired myelination, and reactive astrogliosis^54,55,57^. We harvested brain tissue from P14-15 mice and found the expected cortical hypertrophy and elevated p-S6 in the forebrain of Tsc1-cKO mice (Fig. 5a, b and Supplementary Fig. 5d, e). Strikingly, when we examined the brains of Tsc1-cKO;Raptor-cKO mice, we found decreased overall brain size, severely reduced cortical thickness, and lack of a clear hippocampal structure in comparison to Tsc1-WT;Raptor-WT animals (Fig. 5c and Supplementary Fig. 5f, h, i), despite near normal survival of these mice. In contrast, heterozygous deletion of *Rptor* in Tsc1-cKO animals did not cause gross changes in overall brain architecture or weight (Fig. 5d and Supplementary Fig. 5g–i). Underdeveloped cortical and hippocampal structures were also observed in Tsc1-WT;Raptor-cKO mice (Supplementary Fig. 5j), indicating that impaired forebrain development was due to homozygous *Rptor* deletion alone, independent of *Tsc1* genotype. These results demonstrate that while embryonic loss of *Tsc1* causes cortical hypertrophy, suppression of mTORC1 signaling leads to severely impaired cortical and hippocampal development, signifying the importance of balanced mTOR activity during forebrain development.

**Fig. 6:**
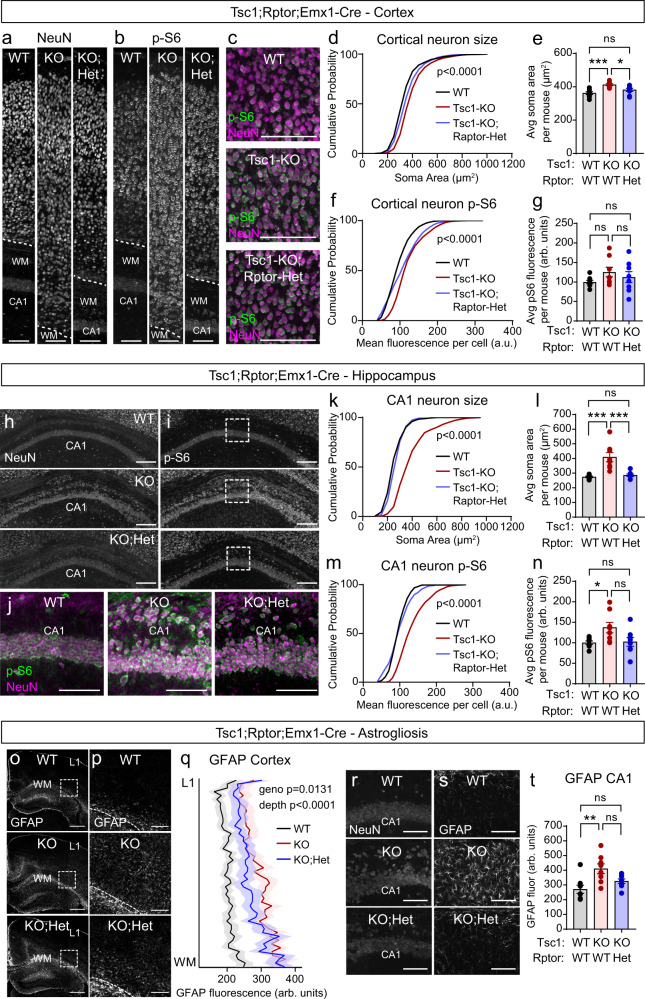
Heterozygous deletion of *Rptor* improves cellular phenotypes in Tsc1-cKO mice. **a**, **b** Representative images of somatosensory cortex showing NeuN (**a**) and p-S6 Ser240/244 (**b**) immunostaining in Tsc1-WT;Rptor-WT (WT), Tsc1-KO;Rptor-WT (KO), and Tsc1-KO;Rptor-Het (KO;Het) mice. Scale bars = 100 μm. **c** Representative zoomed-in images of the cortex showing p-S6 (green) and NeuN (magenta) immunostaining for the indicated genotypes. Scale bars = 100 μm. **d** Cumulative distributions of cortical neuron soma area for the indicated genotypes. **e** Mean ± SEM cortical neuron soma area per mouse. **f** Cumulative distributions of cortical p-S6 levels per neuron. **g** Mean ± SEM cortical neuron p-S6 levels per mouse. **h**, **i** Representative images of hippocampal area CA1 from mice of the indicated genotypes showing NeuN (**h**) and p-S6 (**i**) immunostaining. Scale bars = 250 μm. **j** Representative zoomed-in images of the boxed regions in panel (**i**), showing p-S6 (green) and NeuN (magenta) immunostaining for the indicated genotypes. Scale bars = 100 μm. **k** Cumulative distributions of CA1 neuron soma area. **l** Mean ± SEM CA1 neuron soma area per mouse. **m** Cumulative distributions of CA1 p-S6 levels per neuron. **n** Mean ± SEM CA1 neuron p-S6 levels per mouse. **o** Representative images of GFAP immunostaining in the hippocampus and cortex from mice of the indicated genotypes. Scale bars = 500 μm. **p** Zoomed-in images of GFAP immunofluorescence from the boxes regions in panel (**o**). Scale bars = 150 μm. **q** Quantification of GFAP immunofluorescence across cortical layers for mice of the indicated genotypes. Lines represent mean and shaded regions represent SEM. **r**,**s** Representative images of CA1 from mice of the indicated genotypes showing NeuN (**r**) and GFAP (**s**) immunostaining. Scale bars = 100 μm. **t** Mean ± SEM bulk CA1 GFAP fluorescence per mouse. WM white matter, L1 cortical layer 1, CC corpus callosum. For panels (**e**, **g**, **l**, **n**, **t**), dots represent individual mice. See also Supplementary Fig. 6 and Supplementary Table 3. Statistics for this figure are reported in Supplementary Data 1. Source data are provided as a Source Data file.

Given the significant neurodevelopmental abnormalities associated with complete loss of Raptor, we tested whether partial Raptor reduction could improve brain architecture phenotypes in Tsc1-cKO mice. We found that loss of one copy of *Rptor* reduced cortical and hippocampal hypertrophy, ameliorated hippocampal lamination defects, and improved cortical myelination, resulting in an intermediate phenotype between WT and Tsc1-cKO mice (Fig. 5e–k, Supplementary Fig. 6a–c, and Supplementary Table 3). At the cellular level, heterozygous loss of *Rptor* reduced mTORC1 signaling, as measured by p-S6 levels, and partially prevented neuronal hypertrophy in the cortex, CA1 and dentate gyrus (DG) (Fig. 6a–n, Supplementary Fig. 6d–g, and Supplementary Table 3).

Glial abnormalities have previously been observed in the *Tsc1*^*fl/fll*^;*Emx1-Cre*^*+*^ mouse model^54,55^. Astrocytes specifically appear dysmorphic, exhibiting hypertrophic processes and increased expression of glial fibrillary acidic protein (GFAP)^54,55^. Consistent with these findings, we observed increased GFAP fluorescence across all cortical layers of Tsc1-cKO mice, which was partially attenuated, particularly in the middle cortical layers, in Tsc1-cKO;Raptor-cHet mice (Fig. 6o–q). We also observed a significant increase in GFAP fluorescence intensity in the hippocampal CA1 region of Tsc1-cKO mice that was restored to near WT levels in Tsc1-cKO;Rptor-cHet mice (Fig. 6r–t). Notably, the severity of phenotypes caused by Tsc1 loss and their ability to be prevented by Raptor reduction were both cell type- and sex-dependent (Supplementary Fig. 6h–k and Supplementary Table 3). Taken together, these results demonstrate that partial reduction of Raptor in the forebrain is sufficient to improve multiple developmental brain phenotypes resulting from loss of Tsc1.

### Hyperexcitability of Tsc1-cKO neurons is reduced by Raptor downregulation

**Fig. 7:**
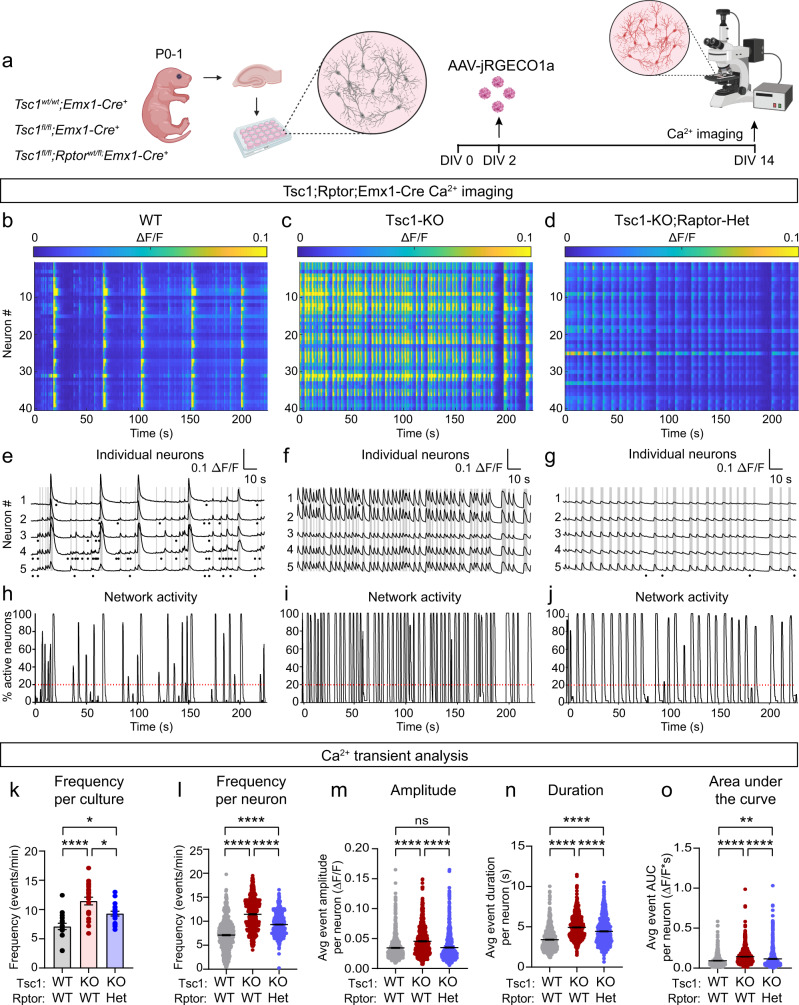
Raptor downregulation reduces hyperactivity of Tsc1-cKO neurons. **a** Schematic of the experiment, created with BioRender.com. **b**–**d** Representative heatmaps of ΔF/F for 40 neurons imaged in a field of view (FOV) from *Tsc1*^*wt/wt*^;*Rptor*^*wt/wt*^;*Emx1-Cre*^+^ (WT, **b**), *Tsc1*^*fl/fl*^;*Rptor*^*wt/wt*^;*Emx1-Cre*^*+*^ (Tsc1-KO, **c**), and *Tsc1*^*fl/fl*^;*Rptor*^*wt/fl*^*;Emx1-Cre*^*+*^ (Tsc1-KO;Raptor-Het, **d**) cultures. **e**–**g** Ca^2+^ transients from 5 representative neurons imaged in a FOV from a WT (**e**), Tsc1-KO (**f**) and Tsc1-KO;Raptor-Het (**g**) culture. Gray lines indicate network events. Black dots represent spontaneous Ca^2+^ transients. **h**–**j** Graphs display the percentage of neurons in the FOV active at a given time for a representative WT (**h**), Tsc1-KO (**i**) and Tsc1-KO;Raptor-Het (**j**) culture. Red dashed lines indicate the threshold for a network event. **k** Mean ± SEM Ca^2+^ transient frequency per culture. Dots represent values from individual cultures. *n* = 20 culture wells from 6 WT and 7 KO and KO;Het independent culture preps. One-way ANOVA, *p* < 0.0001, *F*(2, 57) = 15.82; WT vs KO, ****p < 0.0001; WT vs KO;Het, *p = 0.0178; KO vs KO;Het, *p = 0.0226; Sidak’s multiple comparisons tests. **l** Scatter dot plot of Ca^2+^ transient frequency per neuron. n=800 neurons per genotype. Kruskal–Wallis test, p<0.0001; WT vs KO, ****p < 0.0001; WT vs KO;Het, ****p < 0.0001. KO vs. KO;Het, ****p < 0.0001; Dunn’s multiple comparisons tests. m) Scatter dot plot of average Ca^2+^ transient amplitude per neuron. *n* is the same as for panel (**l**). Kruskal–Wallis test, *p* < 0.0001; WT vs KO, ****p < 0.0001; WT vs KO;Het, *p* = 0.6188; KO vs. KO;Het, ****p < 0.0001; Dunn’s multiple comparisons tests. ns = non-significant. **n** Scatter dot plot of the average Ca^2+^ transient duration per neuron. *n* is the same as for panel (**l**). Kruskal–Wallis test, *p* < 0.0001; WT vs KO, ****p < 0.0001; WT vs KO;Het, ****p < 0.0001; KO vs. KO;Het, ****p < 0.0001; Dunn’s multiple comparisons tests. **o** Scatter dot plot of the average Ca^2+^ transient area under the curve (AUC) per neuron. *n* is the same as for panel (**l**). Kruskal–Wallis test, *p* < 0.0001; WT vs KO, ****p < 0.0001; WT vs KO;Het, **p = 0.0073; KO vs. KO;Het, ****p < 0.0001; Dunn’s multiple comparisons tests. For panels (**l**–**o**), black lines indicate mean ± SEM. Statistical tests were two-sided and *P* values were adjusted for multiple comparisons. See also Supplementary Figs. 7, 8, 10, and 11. Source data are provided as a Source Data file.

*Tsc1*^*fl/fl*^;*Emx1-Cre*^*+*^ mice exhibited spontaneous behavioral seizures that were observable after the first two weeks of life. Given the challenges of performing in vivo EEG analysis in very young animals, and reports that cultured neurons with TSC-associated mutations show network hyperactivity^58,59^, we used calcium imaging of hippocampal cultures from *Tsc1*^*fl/fl*^;*Rptor;*^*wtl/fl*^*Emx1-Cre*^*+*^ mice to test whether Raptor reduction could prevent epileptiform activity. We delivered an AAV encoding the fluorescent calcium indicator jRGECO1a^60^ on DIV 2 and imaged calcium dynamics on DIV 14 (Fig. 7a–j, Supplementary Fig. 7a–h, and Supplementary Movies 1–3). Analysis of individual Ca^2+^ transients revealed that Tsc1-cKO neurons had more frequent events, which were, on average, larger in amplitude and longer in duration than in control neurons (Fig. 7k–n). The presence of larger amplitude and longer duration Ca^2+^ transients in Tsc1 KO neurons suggests increased burst firing activity^60^. We calculated the area under the curve (AUC) for each Ca^2+^ transient and found that the average AUC was significantly larger in Tsc1-cKO neurons (Fig. 7o), although there was no difference in decay kinetics (Supplementary Fig. 7i, j). In cultures from Tsc1-cKO;Raptor-cHet mice, Raptor downregulation reduced the frequency, amplitude, duration, and AUC of Ca^2+^ transients compared to Tsc1 loss alone (Fig. 7k–o). Together these results show that neuronal hyperactivity caused by Tsc1 loss can be ameliorated by partial loss of Raptor.

To investigate how loss of Tsc1 affects network activity patterns, we analyzed network events, which were defined by synchronous activity of more than 20% of neurons in the field of view (see Fig. 7h–j). We found that a significantly larger proportion of neurons participated in each network event in Tsc1-cKO cultures compared to controls and that these events occurred with greater frequency (Supplementary Fig. 7k, l). We measured the percentage of time each culture exhibited coordinated network activity during the recording period and found that this was increased in Tsc1-cKO cultures compared to controls (Supplementary Fig. 7m). While Raptor downregulation did not significantly reduce the frequency or duration of network activity, it was sufficient to reduce the proportion of neurons participating in network events (Supplementary Fig. 7k–m). Frequency distribution histograms of Ca^2+^ transient parameters for each genotype are shown in Supplementary Fig. 8. Collectively, these data show that deletion of *Tsc1* from cultured hippocampal neurons increases neuronal activity and network synchrony and that heterozygous loss of *Rptor* can significantly reduce, but not completely prevent, this epileptiform activity.

### Heterozygous loss of *Tsc1* does not induce network hyperexcitability

Whether partial loss of Tsc1/2 complex function can drive neuronal hyperactivity remains an open question. Several studies have reported that mice with germline heterozygous deletion of *Tsc1* or *Tsc2* do not exhibit spontaneous seizures^61,62^. However, other studies have shown patterns of epileptiform EEG activity in heterozygous mice^63,64^. In addition, studies using cultured human neurons derived from TSC patient cells, which are heterozygous for the *TSC1* or *TSC2* mutation, have reported hyperactivity in some cases^65–67^. To investigate this in our model, we generated *Tsc1*^*wt/fl*^;*Emx1-Cre*^*+*^ (Tsc1-cHet) mice and found that they had normal body weight and survival (Supplementary Fig. 9a–c), with no spontaneous behavioral seizures observed. We prepared hippocampal cultures from these mice and found that in contrast to homozygous *Tsc1* deletion, heterozygous deletion did not induce somatic hypertrophy, increase p-S6, or reduce p-Akt levels (Supplementary Fig. 9d–j).

We transduced Tsc1-cHet cultures with AAV-jRGECO1a and examined whether loss of one copy of *Tsc1* altered Ca^2+^ activity dynamics (Supplementary Figs. 10, 11 and Supplementary Movies 4 and 5). Tsc1-cHet neurons showed no difference in the frequency of Ca^2+^ transients per culture and had a small decrease in the frequency of events per neuron (Supplementary Fig. 10g, h). The average Ca^2+^ transient amplitude per neuron was slightly larger in Tsc1-cHet cultures compared to WT; however, the duration and AUC were smaller than in WT neurons, with no change in decay time (Supplementary Fig. 10i–k and Supplementary Fig. 11c, d). Analysis of network events showed that the frequency and total duration of network events were similar between control and Tsc1-cHet cultures, although a smaller proportion of Tsc1-cHet cells participated in network events (Supplementary Fig. 10l–n). Frequency distribution histograms of Ca^2+^ transient parameters for Tsc1-WT and Tsc1-cHet cultures are shown in Supplementary Fig. 11e–l. Together these data indicate that while loss of one copy of *Tsc1* causes some alterations in Ca^2+^ dynamics in mouse hippocampal neurons, it is not sufficient to induce neuronal or network hyperactivity.

### Postnatal Raptor reduction using AAV-shRptor

The results described above show that ~50% reduction of Raptor protein concurrent with *Tsc1* deletion can prevent mTORC1 hyperactivity and improve multiple developmental and functional phenotypes resulting from Tsc1 loss. To determine whether Raptor reduction could be used therapeutically, we used a viral approach to reduce Raptor expression postnatally in the forebrain of Tsc1-cKO mice. Our biochemical experiments indicated that ~80% loss of Raptor may be most effective at normalizing mTORC1 and mTORC2 signaling in neurons with *Tsc1* deletion (see Fig. 3). To achieve this, we generated AAVs encoding an shRNA targeting *Rptor* (shRptor)^68^ or a scrambled control (shControl) under a ubiquitously expressed promoter. We transduced hippocampal cultures from *Tsc1*^*wt/wt or fl/fl*^;*Emx1-Cre*^*+*^ mice with these viruses and found that shRptor reduced Raptor protein levels by ~90% in WT neurons and 65–70% in Tsc1-cKO neurons (Supplementary Fig. 12a–h). The lesser reduction of Raptor in Tsc1-cKO cultures compared to WT was likely due to upregulation of Raptor protein in these cells (Supplementary Fig. 12d). In Tsc1-cKO cultures, shRptor partially reduced p-S6 levels, relieved p-Akt suppression, and completely rescued neuronal hypertrophy (Supplementary Fig. 12f, h–j). In this experiment, in which *Tsc1* was deleted embryonically, we observed a significant increase in 4E-BP1 phosphorylation in Tsc1-cKO neurons treated with shControl (Supplementary Fig. 12g). Similar to rapamycin treatment^68^, 4E-BP1 phosphorylation was resistant to shRptor (Supplementary Fig. 12g).

**Fig. 8:**
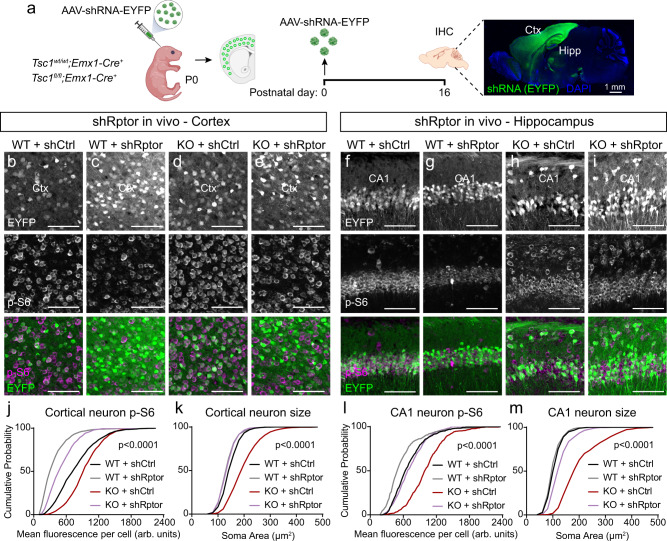
Postnatal Raptor reduction improves cellular phenotypes in Tsc1-cKO mice. **a** Schematic of the experiment, created with BioRender.com. Example sagittal brain image of an injected mouse showing EYFP expression in green and DAPI in blue. This experiment was replicated twice. Ctx cortex, Hipp hippocampus. Scale bar = 1 mm. **b**–**e** Representative images of the somatosensory cortex of *Tsc1*^*wt/wt*^;*Emx1-Cre*^*+*^ (WT, **b**, **c**) and *Tsc1*^*fl/fl*^;*Emx1-Cre*^*+*^ (KO, **d**, **e**) mice injected with either shRptor-EYFP or shCtrl-EYFP virus showing EYFP (top panels) and p-S6 240/244 immunostaining (middle panels). Bottom panels show merged images. Scale bars = 100 μm. **f**–**i** Representative images of the CA1 region of WT (**f**, **g**) and KO (**h**, **i**) mice injected with shRptor-EYFP or shCtrl-EYFP virus showing EYFP (top panels) and p-S6 240/244 immunostaining (middle panels). Bottom panels show merged images. Scale bars = 100 μm. **j** Cumulative distributions of p-S6 levels in cortical EYFP+ neurons for the indicated genotypes. *n* = 1086 WT+shCtrl, 1095 WT+shRptor, 1095 Tsc1-KO+shCtrl, and 1087 Tsc1-KO+shRptor neurons from 6 mice per group. Kruskal–Wallis test, *p* < 0.0001; WT+shCtrl vs WT+shRptor, *p* < 0.0001; WT+shCtrl vs Tsc1-KO+shCtrl, *p* < 0.0001; WT+shCtrl vs Tsc1-KO+shRptor, *p* < 0.0001; Tsc1-KO+shCtrl vs Tsc1-KO+shRptor, *p* < 0.0001; Dunn’s multiple comparison tests.**k** Cumulative distributions of EYFP+ cortical neuron soma area for the indicated genotypes. *n* is the same as in panel (**j**). Kruskal–Wallis test, *p* < 0.0001; WT+shCtrl vs WT+shRptor, *p* < 0.0001; WT+shCtrl vs Tsc1-KO+shCtrl, *p* < 0.0001; WT+shCtrl vs Tsc1-KO+shRptor, *p* < 0.0001; Tsc1-KO+shCtrl vs Tsc1-KO+shRptor, *p* < 0.0001; Dunn’s multiple comparison tests. **l** Cumulative distributions of p-S6 levels in CA1 EYFP+ neurons for the indicated genotypes. *n* = 514 WT+shCtrl, 507 WT+shRptor, 512 Tsc1-KO+shCtrl, and 497 Tsc1-KO +shRptor neurons from 6 mice per group. Kruskal–Wallis test, *p* < 0.0001; WT+shCtrl vs WT+shRptor, *p* < 0.0001; WT+shCtrl vs Tsc1-KO+shCtrl, *p* < 0.0001; WT+shCtrl vs Tsc1-KO+shRptor, *p* = 0.4315; Tsc1-KO+shCtrl vs Tsc1-KO+shRptor, *p* < 0.0001; Dunn’s multiple comparison tests. **m** Cumulative distributions of EYFP+ CA1 neuron soma area for the indicated genotypes. *n* is the same as for panel (**l**). Kruskal–Wallis test, *p* < 0.0001; WT+shCtrl vs WT+shRptor, *p* = 0.2318; WT+shCtrl vs Tsc1-KO+shCtrl, *p* < 0.0001; WT+shCtrl vs Tsc1-KO+shRptor, *p* < 0.0001; Tsc1-KO+shCtrl vs Tsc1-KO+shRptor, *p* < 0.0001; Dunn’s multiple comparison tests. Statistical tests were two-sided and *P* values were adjusted for multiple comparisons. See also Supplementary Figs. 12, 13. Source data are provided as a Source Data file.

To examine the efficacy of postnatal Raptor downregulation as a therapeutic strategy, we injected AAV9-shRptor-EYFP or shControl into the cortex and hippocampus of P0 *Tsc1*^*fl/fl*^;*Emx1-Cre*^*+*^ (Tsc1-cKO) and *Tsc1*^*wt/wt*^;*Emx1-Cre*^*+*^ (WT) mice (Fig. 8a). We examined brain sections from P16 mice and found that shRptor consistently reduced the p-S6 levels and soma size of Tsc1-cKO neurons within the cortex and hippocampus (Fig. 8b–m and Supplementary Fig. 13). We noted that the degree of reduction compared to shControl-treated WT neurons varied by brain region, ranging from an intermediate phenotype in the DG, normalization to WT levels in CA1, and reduction below WT levels in the somatosensory cortex (Fig. 8b–m and Supplementary Fig. 13). In WT mice, shRptor strongly downregulated neuronal p-S6 levels in the cortex and CA1, but not in the DG, and modestly reduced soma size within all regions (Fig. 8b–m and Supplementary Fig. 13). Thus, postnatal downregulation of Raptor improved cellular phenotypes in mice with prenatal *Tsc1* deletion.

**Fig. 9:**
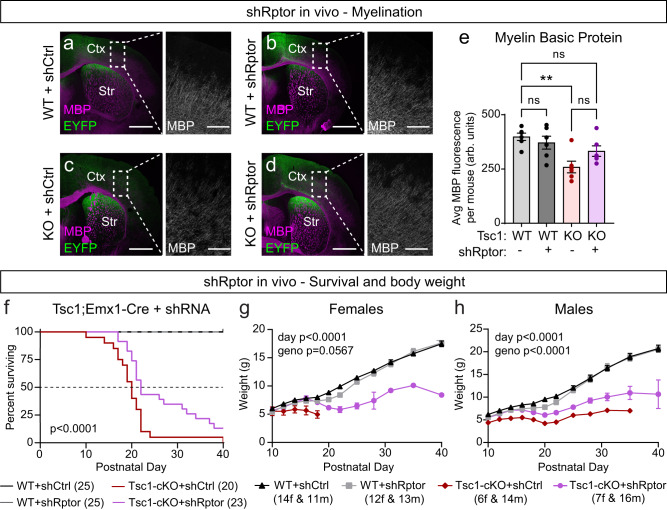
Postnatal Raptor reduction improves myelination and extends survival of Tsc1-cKO mice. **a**–**d** Representative images of somatosensory cortex from mice of the indicated genotypes and treatments, showing EYFP (green) and myelin basic protein (MBP, magenta) immunostaining. Scale bars = 1 mm. Ctx = cortex, Str = striatum. Right panels show zoomed-in images of MBP immunostaining within the boxed cortical regions; scale bars = 250 μm. **e** Mean ± SEM bulk MBP fluorescence intensity in the boxed cortical regions shown in panels (**a**–**d**) for the indicated genotypes. Dots represent individual mice. *n* = 6 animals per condition. Kruskal–Wallis, *p* = 0.0114; WT+shCtrl vs WT+shRptor, *p* > 0.9999; WT+shCtrl vs Tsc1-KO+shCtrl, **p = 0.0077; WT+shCtrl vs Tsc1-KO+shRptor, *p* = 0.4833; Tsc1-KO+shCtrl vs Tsc1-KO+shRptor, *p* = 0.4833; Dunn’s multiple comparison tests. Tests were two-sided and *P* values were adjusted for multiple comparisons. ns = non-significant. **f** Survival analysis of *Tsc1*^*wt/wt*^;*Emx1-Cre*^*+*^ (WT) and *Tsc1*^*fl/fl*^;*Emx1-Cre*^*+*^ (KO) mice injected with shRptor or shControl. The starting number of mice for each genotype is indicated in parentheses. Log-rank Mantel–Cox test *P* value is shown. **g**, **h** Mean ± SEM body weight in grams measured from postnatal day 10–40 for mice of the indicated genotype, treatment and sex. The starting number of mice is indicated in parentheses. f = females, m = males; Mixed-effects model (REML) with Geisser-Greenhouse correction statistics: Females (**g**), day *p* <0.0001, *F*(2.310, 70.56) = 380.3; geno *p* = 0.0567, *F*(3, 35) = 2.759. Males (**h**) day *p* < 0.0001, *F*(1.638, 62.53) = 299.7; geno *p* <0.0001, *F*(3, 50) = 13.58. Multiple comparisons testing was not performed. See also Supplementary Tables 1, 2. Source data are provided as a Source Data file.

Decreased myelination in Tsc1-cKO mouse models can arise directly from changes in oligodendrocytes^69,70^ or can occur via a non-cell-autonomous mechanism due to altered neuronal signaling^57,71^. In *Tsc1*^*fl/fl*^;*Syn-Cre*^*+*^ mice, in which *Tsc1* is lost selectively from neurons, impaired myelination arises due to increased secretion of connective tissue growth factor from Tsc1-cKO neurons, which negatively regulates oligodendrocyte development^71^. In *Emx1-Cre* mice, Cre is expressed in excitatory neurons but also in some glial cells including oligodendrocytes^53,55^. We tested whether shRptor injection into the forebrain of Tsc1-cKO mice could rescue decreased myelination. We found that shRptor boosted bulk cortical MBP fluorescence in Tsc1-cKO mice to near WT levels (Fig. 9a–e). It was previously shown that *Rptor* deletion from oligodendrocytes reduces myelination^69,72^. Therefore, we hypothesize that the increased MBP expression observed here was likely a result of Raptor reduction in Tsc1-cKO neurons, which led to a non-cell-autonomous increase in cortical myelination.

To determine whether postnatal reduction of Raptor could improve the physical development and lifespan of Tsc1-cKO mice, we injected shRptor into the cortex and hippocampus of P0 pups and assessed survival over the first 40 postnatal days. We found that shRptor significantly extended the survival of Tsc1-cKO mice compared to shControl (Fig. 9f and Supplementary Table 1). We also observed an increase in the body weight of Tsc1-cKO mice injected with shRptor, suggesting improved development (Fig. 9g, h and Supplementary Table 2). Together, these results show that postnatal reduction of Raptor can improve and partially reverse the deleterious effects of loss of Tsc1 in the forebrain.

## Discussion

The goal of this study was to test whether genetic downregulation of mTORC1 or mTORC2 activity could improve brain phenotypes in mouse models of TSC. We found that mTORC1 suppression via Raptor reduction was able to rebalance the activity of both mTOR complexes in the context of Tsc1 loss. While complete embryonic Raptor loss caused severe defects in forebrain development, 50% reduction of Raptor was sufficient to ameliorate several TSC-related phenotypes including signaling abnormalities, cellular alterations, network hyperactivity, and changes in brain architecture. Notably, mTORC2 suppression via loss of Rictor did not confer significant benefit in TSC mouse models, likely because mTORC2 activity is already reduced in these mice. To extend the potential therapeutic relevance of our findings, we showed that postnatal downregulation of Raptor rescued Tsc1-cKO cellular phenotypes to a significant degree and extended survival. Together, these data suggest that Raptor downregulation could be a potential therapeutic approach for the neurological presentations of TSC.

### The contribution of mTORC1 vs. mTORC2 to TSC-related brain phenotypes

The manifestations of TSC have traditionally been thought to arise from mTORC1 hyperactivity^2,73^. However, there are several lines of evidence suggesting that mTORC2 may also be involved. First, rapamycin and its derivatives, which are used to treat TSC, not only strongly inhibit the P70S6K/S6 arm of mTORC1 signaling but also suppress mTORC2 activity in neurons^32^ (see Fig. 1). Second, a study in mice lacking *Pten*, an upstream negative regulator of mTORC1, showed that disease-related phenotypes could be prevented by genetic inhibition of mTORC2 but not mTORC1^42^. However, these findings contrasted with a prior study showing that pharmacologic P70S6K inhibition or *Rptor* deletion could improve brain phenotypes in *Pten*^*+/−*^ mice^74^. Third, several different mouse models of TSC exhibit a defect in hippocampal mGluR-dependent LTD^44–47^. It was shown that mTORC2 is the most relevant complex that regulates this form of synaptic plasticity, which is not affected by deletion of *Rptor*^43^. Taken together, this leaves open the possibility that mTORC2 may be a relevant therapeutic target for TSC.

Here we deleted *Rptor* or *Rictor* in mouse models of TSC to determine whether suppression of mTORC1 or mTORC2 could improve disease-related phenotypes. We found that while reduction of Raptor levels ameliorated multiple TSC-related phenotypes, loss of Rictor did not significantly improve mTOR signaling abnormalities, neuronal hypertrophy, physical development, or premature mortality of Tsc1-cKO mice. This indicates that while reductions in Pten or the Tsc1/2 complex both increase mTORC1 signaling, the differences in how loss of these proteins affect other parts of the PI3K/Akt/mTOR signaling network need to be considered when designing a therapeutic strategy. In the case of *Pten* deletion, Akt, mTORC1 and mTORC2 signaling are all upregulated since Pten normally suppresses PI3K-dependent activation of Akt upstream of mTORC1 and mTORC2^42,75–78^. Disruption of the Tsc1/2 complex also leads to increased mTORC1 signaling, which is due to loss of its GAP activity towards Rheb, a direct activator of mTORC1^79^. However, in contrast to Pten, loss of Tsc1/2 leads to decreased mTORC2-dependent phosphorylation of Akt^9,54,69,80–82^. Given that Raptor downregulation suppressed mTORC1 and boosted mTORC2 activity in Tsc1-cKO neurons, it would be interesting to investigate whether mTORC2 activation could confer benefit in the context of Tsc1/2 loss. Future studies could also explore the effects of Raptor loss on other mTORC1 and mTORC2 targets beyond the canonical read-outs investigated here.

Multiple lines of evidence suggest that there is significant crosstalk between the two mTOR complexes. We found that while chronic rapamycin treatment suppressed both mTORC1 and mTORC2 signaling, *Rptor* deletion increased Akt phosphorylation at the mTORC2 site, in both WT and Tsc1-cKO neurons, suggesting enhanced mTORC2 activity. Similar increases in p-Akt S473 have been reported in other cell types with *Rptor* deletion or knock-down^38,69,83^. In addition, it was shown that concomitant deletion of *Rptor* and *Pten* from forebrain neurons led to a stronger upregulation of p-Akt than observed with loss of *Pten* alone^42^. There are several possible mechanisms whereby this crosstalk between mTORC1 and mTORC2 could occur. First, in conditions where mTORC1 signaling is suppressed (e.g. with *Rptor* deletion), there may be reduced negative feedback to upstream regulators of Akt, which would lead to increased Akt phosphorylation^41,81,84^. Second, it has been shown in other systems that P70S6K, a direct target of mTORC1, can phosphorylate two mTORC2 components, Rictor and mSin1, and inhibit mTORC2-dependent Akt phosphorylation^85–87^. In this case, Raptor reduction and reduced mTORC1 activity would lead to decreased Rictor phosphorylation, thereby boosting mTORC2-dependent phosphorylation of Akt. Third, in the case of Raptor reduction, it is possible that limited Raptor protein availability leads to decreased formation of mTORC1, which in turn may free up more mTOR complex proteins to form mTORC2, leading to its enhanced activity. Further work is needed to clarify the mechanistic interactions between mTORC1 and mTORC2 signaling in neurons. Regardless of the mechanism, our data show that partial reduction of Raptor alone, in the absence of direct manipulations to mTORC2, can rebalance the signaling of both mTOR complexes in Tsc1-cKO neurons by constraining mTORC1 signaling and releasing inhibition of mTORC2.

### The consequences of heterozygous versus homozygous loss of *Tsc1*

The mouse model of TSC used here has embryonic deletion of *Tsc1* from most forebrain excitatory neurons and some glial cells. This model recapitulates several disease-relevant phenotypes including spontaneous seizures, abnormal brain anatomy, neuronal hypertrophy, hypomyelination and astrogliosis^54,55^. However, the phenotypes are generally more severe than in individuals with TSC as*Tsc1*^*fl/fl*^;*Emx1-Cre*^*+*^ mice also have reduced body weight, fail to thrive, and exhibit premature mortality in the first few weeks of life. It is important to note that individuals with TSC harbor germline heterozygous mutations in either the *TSC1* or *TSC2* genes, although somatic mosaicism can also occur^88–90^. One prevailing model is that during embryonic brain development, somatic second-hit mutations cause loss of heterozygosity and mTORC1 hyperactivity, leading to the formation of cortical malformations, which can become seizure foci^73,91^. In support of this model, we found that loss of one copy of *Tsc1* from excitatory forebrain neurons was not sufficient to induce seizures, consistent with previous reports^61,92^. To investigate this further, we generated primary hippocampal cultures from Tsc1-cHet mice and found that they did not exhibit mTORC1 activation or neuronal or network hyperactivity. Previous reports have demonstrated that heterozygous loss of *Tsc1* or *Tsc2* from other neuron types can induce synaptic and behavioral changes^46,61,62,92,93^. Therefore, further work is needed to clarify which TSC-related phenotypes may be driven by haploinsufficiency and which require complete disruption of TSC1/2 complex activity.

### Importance of the developmental timing of mTOR perturbations

Balanced mTOR signaling is crucial for proper brain development^54,56,94–96^. In line with this, we found that embryonic mTORC1 suppression via homozygous deletion of *Rptor* from forebrain excitatory neurons led to stunted body and brain development. In particular, Raptor-cKO mice had a severely underdeveloped cortex and failed to form a discernable hippocampal structure, which occurred independent of *Tsc1* genotype. These results are consistent with a prior study that disrupted Raptor expression broadly in neuronal progenitors (using *Rptor*^*fl/fl*^*;Nestin-Cre*^*+*^ mice), which led to microcephaly and perinatal mortality of Raptor-cKO mice^95^. Despite significant deficits in forebrain development, here we found that *Rptor*^*fl/fl*^*;Emx1-Cre*^*+*^ mice had remarkably normal survival, with the majority of Raptor-cKO mice surviving into adulthood (>P150). Postnatal disruption of Raptor has also been investigated using *Rptor*^*fl/fl*^*;CamkIIa-Cre*^*+*^ mice. In this model, Raptor-cKO mice have fully developed hippocampal and cortical structures, however, these brain regions are smaller than in WT littermates^97^. Together these studies highlight the importance of mTORC1 signaling for neural development and show that the developmental timing and number or type of cells impacted by mTORC1 alterations defines the severity of the phenotypes.

### Therapeutic considerations

One of the goals of this study was to test whether genetic suppression of mTORC1 could have beneficial outcomes in mouse models of TSC and avoid side effects associated with rapalog treatment, which strongly inhibits both mTORC1 and mTORC2 signaling. While mTORC2/Akt suppression might be beneficial for anti-tumor drugs, suppression of Akt in the brain is associated with neuronal cell death^98^ and has been implicated in synaptic alterations underlying bipolar disorder^99,100^. Here we found that it was possible to titrate Raptor protein levels to achieve near complete normalization of mTOR signaling in Tsc1-cKO neurons, as measured by p-S6 and p-Akt. Importantly, reduction of Raptor did not strongly suppress mTORC2, as chronic rapamycin does, and in fact boosted mTORC2-dependent phosphorylation of Akt and normalized it in the context of Tsc1 loss. However, Raptor inhibition did not equally affect all mTORC1 pathway targets. Similar to rapamycin treatment the P70S6K/S6 branch of mTORC1 signaling was preferentially suppressed by Raptor loss with less impact on 4E-BP1 phosphorylation. Despite this, reduction of Raptor was sufficient to improve neuronal hypertrophy, network excitability, brain architecture, myelination, and survival in Tsc1-cKO mice. Heterozygous deletion of *Rptor* has also been shown to improve functional and behavioral phenotypes in mice with loss of *Tsc1* from dopamine neurons, suggesting that this approach could have utility across different neuron types^101^. Compared to systemic administration of small molecule drugs, a gene-based therapy has the advantage of potentially being targetable to specific tissues or cell types, which would avoid on-target, off-tissue side-effects that are a limitation to the chronic use of rapalogs^15^.

The Raptor manipulations tested here were consistently effective across multiple functional and anatomical read-outs both in vitro and in vivo. However, they did not provide complete prevention or rescue. In the genetic prevention experiments, this may be because we could maximally achieve 50% loss of Raptor with heterozygous deletion, whereas our biochemical data suggest that ~80% loss would be most beneficial. With Raptor shRNA, we were able to achieve 60–70% Raptor downregulation in Tsc1-cKO neurons, which was sufficient to improve cellular phenotypes, body weight, and survival of Tsc1-cKO mice. However, Tsc1-cKO mice treated with shRaptor still exhibited spontaneous behavioral seizures. Stronger Raptor suppression may show even more benefit. In addition, it would be worthwhile testing Raptor reduction in a model with milder or later onset phenotypes to allow more time for therapeutic intervention. Importantly, postnatal reduction of Raptor should avoid the severe developmental impairments caused by embryonic mTORC1 suppression.

In summary, our study highlights Raptor as a relevant therapeutic target for TSC-related brain phenotypes in mouse models, with potential advantages over current pharmacologic approaches.

## Methods

Experimental procedures were approved under a Biological Use Authorization (BUA #351) granted by the University of California, Berkeley campus Committee on Laboratory & Environmental Biosafety (CLEB). All animal procedures and husbandry were carried out in accordance with protocols approved by the University of California, Berkeley Institutional Animal Care and Use Committee (IACUC, protocol #AUP-2016-04-8684-2).

### Mice

Both male and female animals were used for all experiments. The ages are indicated in the methods for each experiment. Mice were housed with same sex littermates in groups of 4–5 animals per cage and kept on a regular 12 hr light/dark cycle (lights on at 7am), with ad libitum access to standard chow and water. Room temperature was set to ~22 °C and humidity was not externally controlled. Mice used for shRNA experiments were housed on an inverse 12 h light/dark cycle with ad libitum access to standard chow and water. Mouse genotypes were confirmed by PCR using genomic DNA obtained from tail samples. Supplementary Table 4 lists the transgenic mouse lines used and genotyping primers. To generate forebrain-specific deletion of *Tsc1*, floxed mice were bred to Emx1-Cre+ males or females and mice heterozygous for Cre were used for experiments. Two tail samples from the *Tsc1;Rictor;Emx1-Cre* and *Tsc1;Rptor;Emx1-Cre* lines were sent to Taconic for Single Nucleotide Polymorphism (SNP) genetic background testing. The mice had a mixed genetic background with the highest percentage strains being C57BL/6J, C57BL/6NTac, and 129S6/SvEvTac (Table 1).

**Table 1:** Single Nucleotide Polymorphism (SNP) genetic background testing

Mouse line	Sex	% C57BL/6NTac	% C57BL/6J	% 129S6/SvEvTac
*Tsc1;Rictor;Emx1-Cre*	M	66.18%	64.26%	61.77%
*Tsc1;Rictor;Emx1-Cre*	F	68.42%	66.65%	61.86%
*Tsc1;Raptor;Emx1-Cre*	M	77.91%	78.34%	58.42%
*Tsc1;Raptor;Emx1-Cre*	M	79.51%	80.00%	57.28%

### Primary hippocampal cultures

Dissociated hippocampal cultures were prepared from postnatal day 0-1 (P0-1) mice using standard protocols. Briefly, hippocampi from 2-3 pups (floxed mouse lines, Figs. 1–3 and Supplementary Figs. 1–4) or from single pups (Emx1-Cre mouse lines, Fig. 7 and Supplementary Figs. 7–12) were dissected on ice. The tissue was dissociated using 34.4 μg/ml papain in dissociation media (HBSS Ca^2+^, Mg^2+^ free, 1 mM sodium pyruvate, 0.1% D-glucose, 10 mM HEPES buffer) and incubated for 3 min at 37^o^C. Tissue digestion was stopped by incubation in trypsin inhibitor (1 mg/ml) in dissociation media at 37^o^C for 4 min. After trypsin inhibition, dissociation media was carefully removed and the tissue was gently manually triturated in 5 ml plating media (MEM, 10% FBS, 0.45% D-Glucose, 1 mM sodium pyruvate, 1 mM L-glutamine). Cell density was counted using a TC10 Automated cell counter (Bio-Rad) and ~(2–2.25) × 10^5^ neurons were plated for each experiment. For western blotting and Ca^2+^ imaging experiments, neurons were plated onto 24-well plates pre-coated with Poly-D-Lysine (PDL) (Corning, Cat # 08774271). For immunocytochemistry, neurons were plated onto 12 mm glass coverslips pre-coated overnight at room temperature (RT) with 0.5 mg/ml PDL in 0.1 M borate buffer (pH 8.5). On the plating day, the coverslips were rinsed 4 times with sterile water and then coated with 20 μg/ml laminin (GIBCO, 23017015) in 1x PBS for ~1.5 h at 37^o^ C. Subsequently, coverslips were rinsed 3 times with sterile water, and 400 μl of plating media were added prior to adding the dissociated neurons. For all cultures, plating media was removed after 3 h and 900 μl maintenance media (Neurobasal media (Fisher Scientific # 21103-049) with 2 mM glutamine, pen/strep, and B-27 supplement (Invitrogen # 17504-044) were added per well. After 4 days in vitro (DIV 4), 1 μM Cytosine β-D-arabinofuranoside (Sigma-Aldrich # C6645) was added to prevent glial proliferation. Cultures were maintained in maintenance media for 14 - 18 days with partial media changes every 4 days.

### Adeno-associated virus (AAV) transduction of primary cultures

For hippocampal culture experiments, AAVs were added on DIV 2, except for the shRNA experiments in which AAVs were added on DIV 1. Amounts of AAVs were chosen after titration experiments for each virus to accomplish either maximum or sparse transduction efficiency while maintaining low toxicity levels. In primary cultures from *Tsc1*^*fl/fl*^, *Tsc1*^*fl/fl*^;*Rptor*^*fl/fl*^, *Tsc1*^*fl/fl*^;*Rptor*^*wt/fl*^ and *Tsc1*^*fl/fl*^;*Rictor*^*fl/fl*^ mice, which were used for western blotting experiments, we aimed for >95% transduction efficiency using AAV1 human Synapsin 1 (*SYN1*, hSyn) promoter-driven Cre-GFP or GFP to generate mutant and control cultures, respectively. For immunocytochemistry experiments, we aimed for sparse transduction to resolve individual neurons using AAV1-CBA-mCherry-Cre (nuclear localized), AAV9-CAG-FLEX-tdTomato, and AAV5-hSyn-GFP. To determine the percentage of Cre-expressing neurons in primary hippocampal cultures from Emx1-Cre+ pups we used AAV1-CAG-FLEX-GFP at a high titer (see Supplementary Fig. 7a) and found that 82.9% of the cultured hippocampal neurons expressed the GFP Cre reporter. For calcium imaging experiments, we transduced neurons with AAV1-hSyn-jRGECO1a aiming for >95% transduction efficiency. For shRNA experiments, we transduced neurons with AAV9-hU6-shRNA-EYFP on DIV 1 and achieved 85–90% transduction. See Supplementary Table 5 for the list of viruses, source, titer and number of viral genomes (vg) used.

### Protein extraction and western blot analysis

Hippocampal cultures were harvested on DIV 14–18. Neurobasal media was aspirated from one well at a time and wells were quickly rinsed with ice cold 1x PBS with Ca^2+^/Mg^2+^ and then 75 μl of lysis buffer were added (lysis buffer: 2 mM EDTA (Sigma: E5134), 2 mM EGTA (Sigma: E3889), 1% Triton-X (Sigma: T8787), and 0.5% SDS (Sigma: 71736) in 1× PBS with Halt phosphatase inhibitor cocktail (ThermoFisher: PI78420) and Complete mini EDTA-free protease inhibitor cocktail (Roche: 4693159001)). Wells were thoroughly scraped, and lysates were collected and sonicated for 5 sec. Total protein was determined by BCA assay (ThermoFisher: PI23227) and 10 μg of protein in 1X Laemmli sample buffer (Bio-Rad:161-0747) were loaded onto 4–15% Criterion TGX gels (Bio-Rad: 5671084). Proteins were transferred overnight at low voltage to PVDF membranes (Bio-Rad: 1620177), blocked in 5% milk in 1x TBS-Tween for one hour at RT, and incubated with primary antibodies diluted in 5% milk in 1x TBS-Tween overnight at 4 °C. The following day, membranes were washed 3 × 10 min in 1x TBS-Tween and incubated with HRP-conjugated secondary antibodies (1:5000) for one hour at RT, washed 6 × 10 min in 1x TBS-Tween, incubated with chemiluminescence substrate (Perkin-Elmer: NEL105001EA) and developed on GE Amersham Hyperfilm ECL (VWR: 95017-661). Membranes were stripped by two 7 min incubations in stripping buffer (6 M guanidine hydrochloride (Sigma: G3272) with 1:150 β-mercaptoethanol) with shaking followed by four two min washes in 1x TBS with 0.05% NP-40 to re-blot on subsequent days.

Bands were quantified by densitometry using ImageJ 1.52p software (NIH). We note that while this method provides accurate measurements for single bands that increase or decrease in intensity it cannot easily resolve changes that pertain to size shifts. For example, for 4E-BP1 in Fig. 1b we observed changes in the size of bands between WT, Tsc1-cKO and Tsc1-cKO + rapamycin conditions but not in their total intensity. For all experiments, phospho-proteins were normalized to their respective total proteins. Histone-3 was used as a loading control for every experiment. Antibody vendors, catalog numbers, and dilutions are listed in Supplementary Table 6 and in the Reporting Summary. All antibodies were used in accordance to manufacturer guidelines and were validated by the manufacturer for use in mouse samples for the specific assays used in this study.

### Immunocytochemistry

On DIV 14, Neurobasal media was carefully aspirated from all wells and coverslips were briefly rinsed with ice cold 1x PBS with Ca^2+^/Mg^2+^ followed by fixation in freshly made 4% PFA (Electron Microscopy Sciences: 15713) in 1x PBS for 10 min. PFA was then removed and the coverslips were washed for 5 min with 1x PBS three times. Coverslips were blocked for one hour at RT in buffer containing 5% normal goat serum (NGS) (ThermoFisher: PCN5000) and 0.3% Triton-X in 1x PBS and incubated in primary antibodies in antibody dilution buffer (1% BSA and 0.3% Triton-X in 1x PBS) overnight at 4 °C. The following day, coverslips were washed three times for 5 min in 1x PBS, incubated with secondary antibodies in antibody dilution buffer (1:500) for one hour at RT and washed three times for 5 min in 1x PBS. Coverslips were mounted onto slides with ProLong Gold Antifade mountant with or without DAPI (ThermoFisher: P36934 or P36935) and allowed to set for one day before imaging. Antibody vendors, catalog numbers, and dilutions are listed in Supplementary Table 6.

For soma size quantification, neuronal cultures from the floxed mouse lines were imaged on an FV1000 Olympus confocal microscope with a 20x objective (using FluoView 1000 software). Neuronal cultures from the Emx1-Cre lines were imaged with a Hamamatsu Orca-er digital camera with a 10x objective and Micro-Manager 1.4 software. Soma area was quantified by manually tracing neuronal cell bodies using ImageJ 1.52p software.

### Rapamycin treatment in vitro

Primary hippocampal cultures were treated chronically for 4 days with rapamycin from DIV 10–14. A stock solution of 0.5 mM rapamycin (LC Laboratories: R-5000) was prepared in ethanol and stored at −20 °C. Rapamycin stock was diluted in Neurobasal media 1:100 prior to use and then added to a final concentration of 50 nM. Rapamycin was first added on DIV 10 and in the final media change on DIV 12.

### Survival and body weight monitoring

Breeder cages from the *Tsc1;Rptor;Emx1-Cre* and *Tsc1;Rictor;Emx1-Cre* lines were monitored daily with minimal disturbance. The birth date of each litter was noted, and the number of pups was recorded. At P11, pups were genotyped, and their body weight was measured. Weight was measured every two days from P11-P21 and every 5 days from P21-P40. Each pup that was found dead in the cage was immediately removed and re-genotyped for confirmation. Mice were handled minimally and with care to reduce stressors that could promote seizures.

For the shRNA intracranial injection experiments, breeder cages from the *Tsc1;Emx1-Cre* line were monitored daily with minimal disturbance. On the birth date (P0), each litter received intracranial injections (see methods below) and the number of pups was recorded. Pups found dead before P5 were excluded from the study. At P10, pups were genotyped and their body weight was measured. Starting at P14, wet food was provided at the bottom of the cage, which was changed on a daily basis. Weight was measured every two days from P10-P22, every three days from P22-31 and then on P35 and P40. Each pup that was found dead in the cage was immediately removed and re-genotyped for confirmation. Mice were handled minimally and with care to reduce stressors that could promote seizures.

### Perfusion and immunohistochemistry

P14-P15 mice were deeply anesthetized by isoflurane and transcardially perfused with ice-cold 1x PBS, followed by 4% PFA solution (Electron Microscopy Sciences: 15713) in 1x PBS using a peristaltic pump (Instech). The brains were removed and post-fixed by immersion in 4% PFA in 1x PBS overnight at 4 °C. Brains were then suspended in 30% sucrose in 0.1 M PB solution at 4 °C until processed. Brains were sectioned coronally at 30 μm on a freezing microtome (American Optical AO 860).

Free-floating brain sections were batch processed to include matched control and experimental samples. With gentle shaking, sections were washed 3 × 15 min in 1x PBS, followed by 1 h incubation at RT with BlockAid blocking solution (Life Tech: B10710). Primary antibodies were diluted in 1x PBS-T (1x PBS + 0.25% Triton-X-100) and applied for 48 h at 4 °C. Sections were washed with cold 1x PBS 3 × 15 min and incubated for 1h at RT with secondary antibodies diluted 1:500 in 1x PBS-T. Sections were then washed in cold 1x PBS 5 × 15 min, mounted on SuperFrost slides (VWR: 48311-703), and coverslipped with Vectashield hard-set mounting media with DAPI (Vector Labs: H-1500). See Supplementary Table 6 for a list of antibodies and dilutions used for immunohistochemistry.

### Confocal microscopy and image analysis

Images of brain sections were taken on either a Zeiss LSM 710 AxioObserver (using ZEN 2010 blue edition software) with 10x or 20x objectives, or an FV3000 Olympus confocal microscope with 4x or 20x objectives (using FluoView 3000 software). For all quantitative comparison experiments, the same microscope and acquisition settings were used for each image and samples were processed in batches to include matched control and experimental samples. All images were processed using ImageJ 1.52p software.

To quantify p-S6 levels and soma area, regions of interest (ROIs) were manually drawn around neuronal bodies on max-projected Z-stack images. The location and the size of the brain region selected for analysis was consistent across genotypes for each experiment. For the cortex, a region within the somatosensory area spanning all layers was selected and ~200 neurons per section were traced. For the CA1, ~70 neurons per section were traced and analyzed. For the dentate gyrus, ~50 neurons per section from a sub-region of the suprapyramidal blade were analyzed per animal. To quantify ectopic cells above CA1, we selected a 249 × 83 μm ROI between the top of CA1 pyramidal layer and white matter and quantified the number of p-S6+ cells.

For myelin basic protein (MBP) analysis, the average bulk fluorescence intensity was calculated from a ~468 x 468 μm sized ROI from a region spanning the retrosplenial area and secondary motor area drawn on max-projected Z-stack images. To examine GFAP expression in the cortex, a line was drawn across the cortical plate spanning from layer I to the border with the white matter. Mean GFAP fluorescence along the line was plotted using plot profile function in ImageJ 1.52p. To examine GFAP expression in CA1, bulk average fluorescence intensity was calculated from a sub-region of CA1 approximately 232 x 81 μm.

For animals that received intracranial injections of AAV9-shRNA-EYFP bilaterally, neurons from both hemispheres were analyzed and pooled to account for potential differences in the injection efficiency. To quantify p-S6 levels, ~185 EYFP+ neurons were manually outlined within the primary somatosensory area including all cortical layers. For the hippocampus, ~85 neurons in CA1 and ~65 neurons in DG from a middle region (along the anterior/posterior axis) of the hippocampus were analyzed per animal. To quantify MBP, two ~621.5 × 621.5 μm ROIs (one per hemisphere) in the primary somatosensory cortex were drawn and bulk MBP fluorescence intensity was quantified and averaged per mouse. All ROIs were drawn on max-projected Z-stack images using ImageJ 1.52p.

### Calcium Imaging

Primary hippocampal cultures from P0-1 *Tsc1;Rptor;Emx1-Cre* mice of different genotypes were plated onto 24 well plates pre-coated with PDL (Corning, Cat # 08774271). On DIV 2, cultures were transduced with AAV1-jRGECO1a (Supplementary Table 5) and maintained for 12 days in Neurobasal media. On DIV 14, neurons were imaged on an AxioObserver.A1 (Zeiss) inverted microscope using a ×10 Zeiss A-Plan objective (Numerical Aperture: 0.25) with wide field fluorescence illumination (X-Cite series 120Q, Lumen Dynamics). Images were taken at 8.91 Hz with a Hamamatsu Orca-er digital camera and Micro-Manager 1.4 software. All imaging conditions including excitation light intensity, camera sensor gain, and exposure time were identical for all calcium imaging experiments. A single field of view (FOV) was imaged from at least 2-3 individual wells per culture (prepped from 1 pup) and approximately 40 neurons were randomly selected and analyzed. Before proceeding to the analysis, we verified that the neurons selected were active at least once during the recording period. At least 3 mice per genotype were examined from at least 3 different litters.

### Calcium imaging analysis

Data analysis was performed using ImageJ 1.53c and custom programs written in Matlab 2020a. The code is available at https://github.com/FranklinHolme/spontaneous-activity, 10.5281/zenodo.6614378.

#### Pre-processing

Circular ROIs corresponding to neuronal somata were drawn manually in ImageJ on mean intensity projection images of the recorded FOV. Forty ROIs were drawn per FOV, beginning in the upper left quadrant of the image, and extending outward as necessary (to the right, bottom left, and bottom right quadrants, respectively), and were imported into Matlab for further analysis. Movies were motion corrected using the normcorre function^102^, then normalized with respect to baseline, taken to be the minimum intensity projection of the FOV, to generate a ΔF/F movie. It was necessary to use the minimum projection as Tsc1-cKO neurons exhibited high Ca^2+^ activity and thus contained few baseline frames. Ca^2+^ traces were extracted as ΔF/F by computing the mean fluorescence within each ROI at each movie frame.

#### Single event analysis

Individual Ca^2+^ transients were detected by first filtering the ΔF/F traces with a four-frame moving mean, then using Matlab’s findpeaks function to identify peaks in the ΔF/F trace with amplitude >0.5%. Event amplitude was defined as the difference between the event’s peak ΔF/F and the minimum ΔF/F in the preceding inter-event-interval (see Supplementary Fig. 7f, g). Events were excluded from amplitude, duration, and AUC analysis if 1) they occurred <2 s after a previous event or 2) their amplitude was <50% of their absolute ΔF/F, indicating already elevated baseline Ca^2+^. Prior to measurement of event duration and area under the curve (AUC), Matlab’s msbackadj function was used to shift the 1st percentile of the ΔF/F trace within 15 s time windows to zero to reduce the reliance of event AUC on preceding bouts of Ca^2+^ activity. Event initiation and termination were identified by finding the 0.5% ΔF/F threshold (see Supplementary Fig. 7g) crossing preceding and following the event peak. Event termination was alternatively identified when the Ca^2+^ decay following the event peak ΔF/F was interrupted by a ΔF/F increase >1%, indicating the initiation of another event. Events without a clear initiation or termination were excluded from further analysis. AUC was defined as the area under the ΔF/F trace during the event (see Supplementary Fig. 7g) and measured using trapezoidal numerical integration implemented by the trapz function in Matlab. Inter-event-intervals (IEI) were defined as the duration between Ca^2+^ transient peaks.

#### Network event analysis

Network Ca^2+^ events were defined as time intervals over which more than 20% of neuronal ROIs in the imaged area were simultaneously active (active time intervals for a single neuron were defined as full width at half maximum around peaks with prominence ≥ 0.5% ΔF/F; see gray highlighted zones in Fig. 7e–g and Supplementary Fig. 10c, d). We did not use a standard deviation-based threshold, as it would have selectively reduced event detection in Tsc1-cKO cultures, due to their persistent Ca^2+^ activity. Events with a duration <2.5 s were excluded from further analysis. Cell participation in events was defined as the percentage of neurons that were active at any time during the event.

### shRNA constructs

The AAV-Tet3-shRNA plasmid (Addgene plasmid # 85740) was used as a backbone to generate the AAV9-hU6-shRptor-EYFP construct. The restriction enzymes BamHI and XbaI were used to excise the Tet-3 shRNA sequence. The oligonucleotide sequence: 5’-GATCCGCCTCATCGTCAAGTCCTTCAAGAAGCTTGTTGAAGGACTTGACGATGAGGCTTTTTTT-3′ that contains the *Rptor* shRNA sequence^68^ flanked by restriction sites for BamHI and XbaI was subcloned into the plasmid backbone. AAV-hU6-shControl-EYFP (Addgene plasmid # 85741) that contains the 5′-GTTCAGATGTGCGGCGAGT-3′ shRNA sequence was used as a control. For large-scale isolation and purification of the plasmids, DH5α NEB competent cells (New England Biolabs #C2987H) were transformed and Endofree Megaprep (Qiagen # 12381) was performed to generate plasmids for high titer viral packaging. The constructs were subsequently sent to the CLOVER Center at Caltech for packaging into AAV9 virus.

### Intracranial neonatal mouse injections

Neonatal mice (P0) were cryo-anesthetized by placing on ice for ~2–3 min. When the animal was fully anesthetized, confirmed by lack of movement, it was gently placed in a head mold. Each pup received a total of 500 nl of 4x diluted AAV9 (AAV9-hU6-shRptor-EYFP or AAV9-hU6-shControl-EYFP, see Supplementary Table 5 for titer information) spread across 4 injections (2 per hemisphere). Two 150 nl injections were made into the cortex and two 100 nl injections were made into the dorsal hippocampus. Cortical injections were made halfway between bregma and lambda approximately 0.6 mm from the sagittal suture and 0.5–0.6 mm ventral to the surface of the skull. Hippocampal injections were made approximately 0.5 mm anterior to lambda with the injection sites ~0.5 mm from the sagittal suture and 1 mm ventral to the surface of the skull.

### Statistics and reproducibility

Statistical analyses and graphing were performed using GraphPad Prism software (versions 7–9). All datasets were first analyzed using the D’Agostino and Pearson normality test, and then parametric or non-parametric two-sided statistical tests were employed accordingly to determine significance. Normally distributed datasets were analyzed using Welch’s *t* tests when comparing two groups or a one-way ANOVA with Holm-Sidak’s multiple comparison tests when comparing three or more groups. Datasets that did not pass the normality test were analyzed using a Mann–Whitney test when comparing two groups or the Kruskal–Wallis test with Dunn’s multiple comparisons tests when comparing three or more groups. Cumulative distributions were analyzed using Kolmogorov–Smirnov tests (when comparing two groups) or the Kruskal–Wallis test with Dunn’s multiple comparisons tests (when comparing three or more groups). Survival curves were analyzed using the Log-rank (Mantel–Cox) test. Regression models were analyzed either with a two-sided Pearson correlation (linear regression) or Spearman correlation (non-linear regression). GFAP fluorescence across cortical layers was analyzed with a two-way ANOVA with Geisser-Greenhouse correction. Significance was set as *p < 0.05, **p < 0.01, ***p < 0.001, and ****p < 0.0001. *P* values were corrected for multiple comparisons. All experiments were completed one time unless otherwise stated in the figure legend.

### Reporting summary

Further information on research design is available in the Nature Research Reporting Summary linked to this article.

## Supplementary information

Supplementary Information

Description of Additional Supplementary Files

Supplementary Data 1

Supplementary Movie 1

Supplementary Movie 2

Supplementary Movie 3

Supplementary Movie 4

Supplementary Movie 5

Reporting Summary

## Data Availability

The data generated in this study are provided in the Source Data file. Additional data generated during this study are available from the corresponding author on reasonable request. Data will be shared within three months of the request. Source data are provided with this paper.

## Source data

Source Data

## Code availability

Code for the calcium imaging analysis is available at https://github.com/FranklinHolme/spontaneous-activity. 10.5281/zenodo.6614378.
