# Supplementary material for: Raptor downregulation rescues neuronal phenotypes in mouse models of Tuberous Sclerosis Complex
Source: Nat Commun. 2022 Aug 9;13:4665. doi: 10.1038/s41467-022-31961-6 (PMC9363483; doi:10.1038/s41467-022-31961-6)
Supplement: Supplementary file 1 — Supplementary Information [file 41467_2022_31961_MOESM1_ESM.pdf]

Supplementary Information

Raptor downregulation rescues neuronal phenotypes in mouse models of Tuberous Sclerosis Complex

Vasiliki Karalis, Franklin Caval-Holme, and Helen S. Bateup

Contents:

13 Supplementary Figures

6 Supplementary Tables

Supplementary References

Supplementary Figure 1

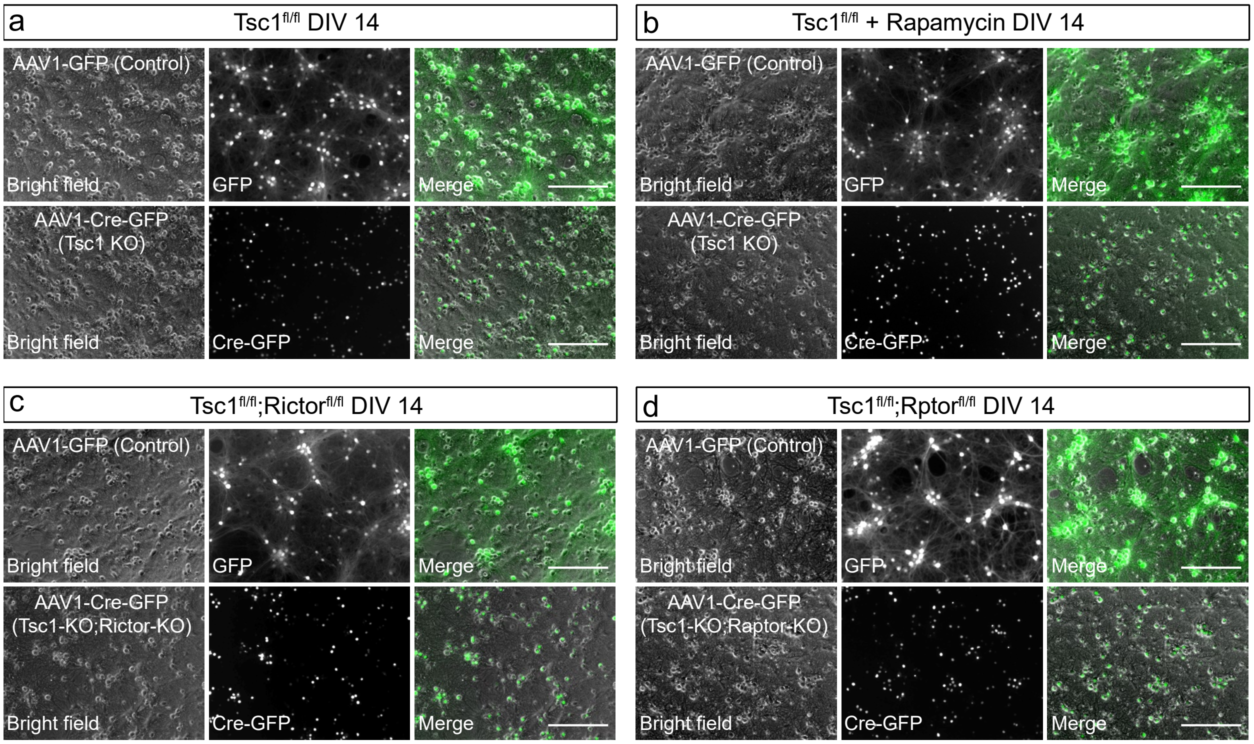

Supplementary Figure 1. Example images of primary hippocampal cultures (related to Figures 1-3).

Representative bright-field and fluorescence images of primary hippocampal cultures from P0-P1 pups imaged on DIV 14. The cultures were transduced on DIV 2 with AAV-GFP (cytosolic expression) or AAV-Cre-GFP (nuclear localized).

a) *Tsc1$^{fl/fl}$* cultures + GFP (Control, top panels) or Cre-GFP (Tsc1 KO, bottom panels)

b) *Tsc1$^{fl/fl}$* cultures + GFP (Control, top panels) or Cre-GFP (Tsc1 KO, bottom panels) treated with rapamycin (50 nM) from DIV 10-14.

c) *Tsc1$^{fl/fl}$;Rictor$^{fl/fl}$* cultures + GFP (Control, top panels) or Cre-GFP (Tsc1-KO;Rictor-KO, bottom panels).

d) *Tsc1$^{fl/fl}$;Rptor$^{fl/fl}$* cultures + GFP (Control, top panels) or Cre-GFP (Tsc1-KO;Raptor-KO, bottom panels)

These experiments were replicated 5 times for panels a and b and three times for panels c and d. All scale bars=250 µm.

Supplementary Figure 2

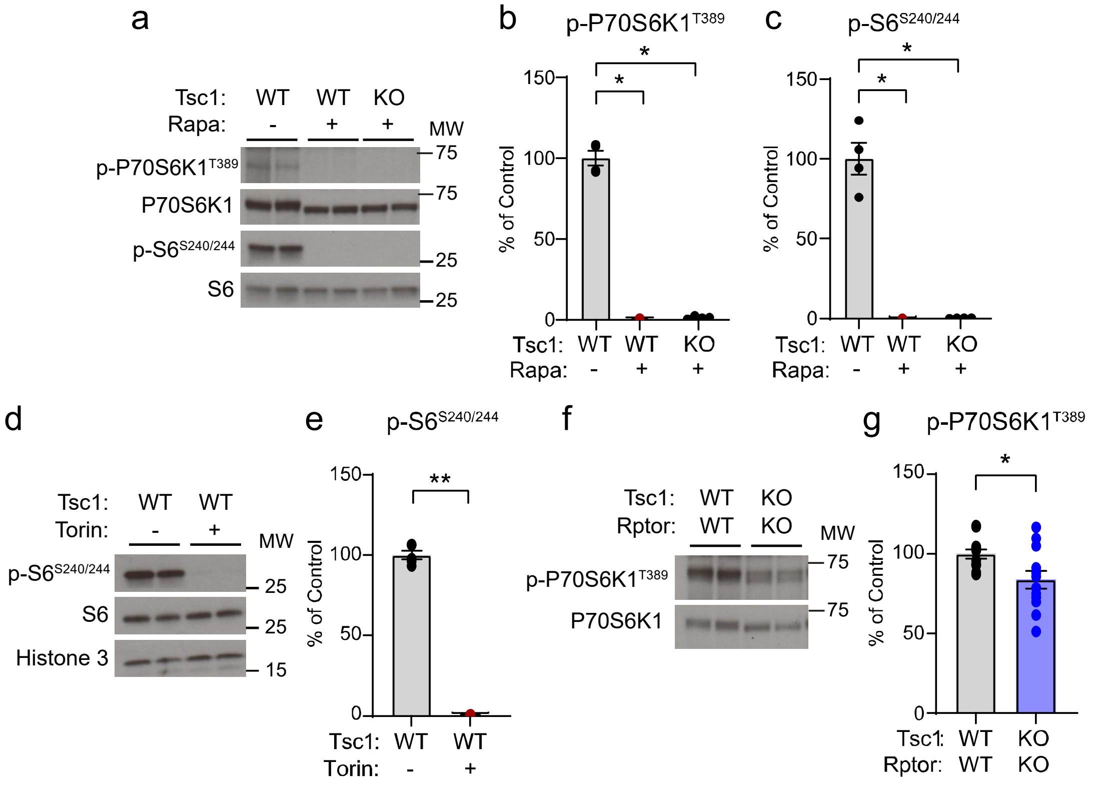

Supplementary Figure 2. Treatment with mTOR inhibitors blocks P70S6K1 and S6 phosphorylation in cultured hippocampal neurons (related to Figures 1 and 2).

a) Representative western blots (WB) from *Tsc1$^{fl/fl}$* hippocampal cultures treated with AAV-GFP (WT) or AAV-Cre-GFP (KO) at DIV 2 and with (+) or without (-) 50 nM rapamycin starting on DIV 10. Cultures were harvested on DIV 14. Two independent samples per genotype are shown; this experiment was replicated two times. MW indicates molecular weight (in kD).

b,c) Bar graphs display WB quantification (mean +/- SEM) for the indicated phospho-proteins, normalized to their respective total proteins, and expressed as a percentage of Control (WT without Rapa) levels. n=4 culture wells from 2 independent cultures per genotype; 1 mouse per culture. p-P70S6K1-T389 (b), Kruskal-Wallis test *p=0.0132; WT vs WT+Rapa, *p=0.0285; WT vs Tsc1-KO+Rapa, *p=0.0482; Dunn's multiple comparison tests. pS6-S240/244 (c), Kruskal-Wallis test *p=0.0145; WT vs WT+Rapa, *p=0.0372; WT vs Tsc1-KO+Rapa, *p=0.0372; Dunn's multiple comparison tests. Statistical tests were two-sided and P values were corrected for multiple comparisons.

d) Representative WB from WT hippocampal cultures treated with (+) or without (-) 50 nM of the mTOR kinase inhibitor Torin for 6-hours and harvested on DIV 14. Two independent samples per genotype are shown; this experiment was replicated 2 times.
e) Bar graphs display WB quantification (mean +/- SEM) for p-S6-S240/244, normalized to total S6 and expressed as a percentage of Control (WT without Torin). n=5 WT (-) Torin and 6 WT (+) Torin culture wells from 2 independent cultures; 1 mouse per culture. Two-sided Mann-Whitney test, **p=0.0043.
f) Representative WB from *Tsc1*$^{fl/fl}$*;Rptor*$^{fl/fl}$ hippocampal cultures treated with AAV-GFP (WT;WT) or AAV-Cre-GFP (KO;KO) at DIV 2 and harvested on DIV 14. Two independent samples per genotype are shown; this experiment was replicated four times.
g) Bar graphs display WB quantification (mean +/- SEM) for p-P70S6K1-Thr389 normalized to total P70S6K1 and expressed as a percentage of Control (WT/WT) levels. n=12 culture wells from 4 independent cultures per genotype; 2 mice per culture. Two-sided Welch's t-test *p=0.0219.
Source data are provided in the Source Data file.

Supplementary Figure 3

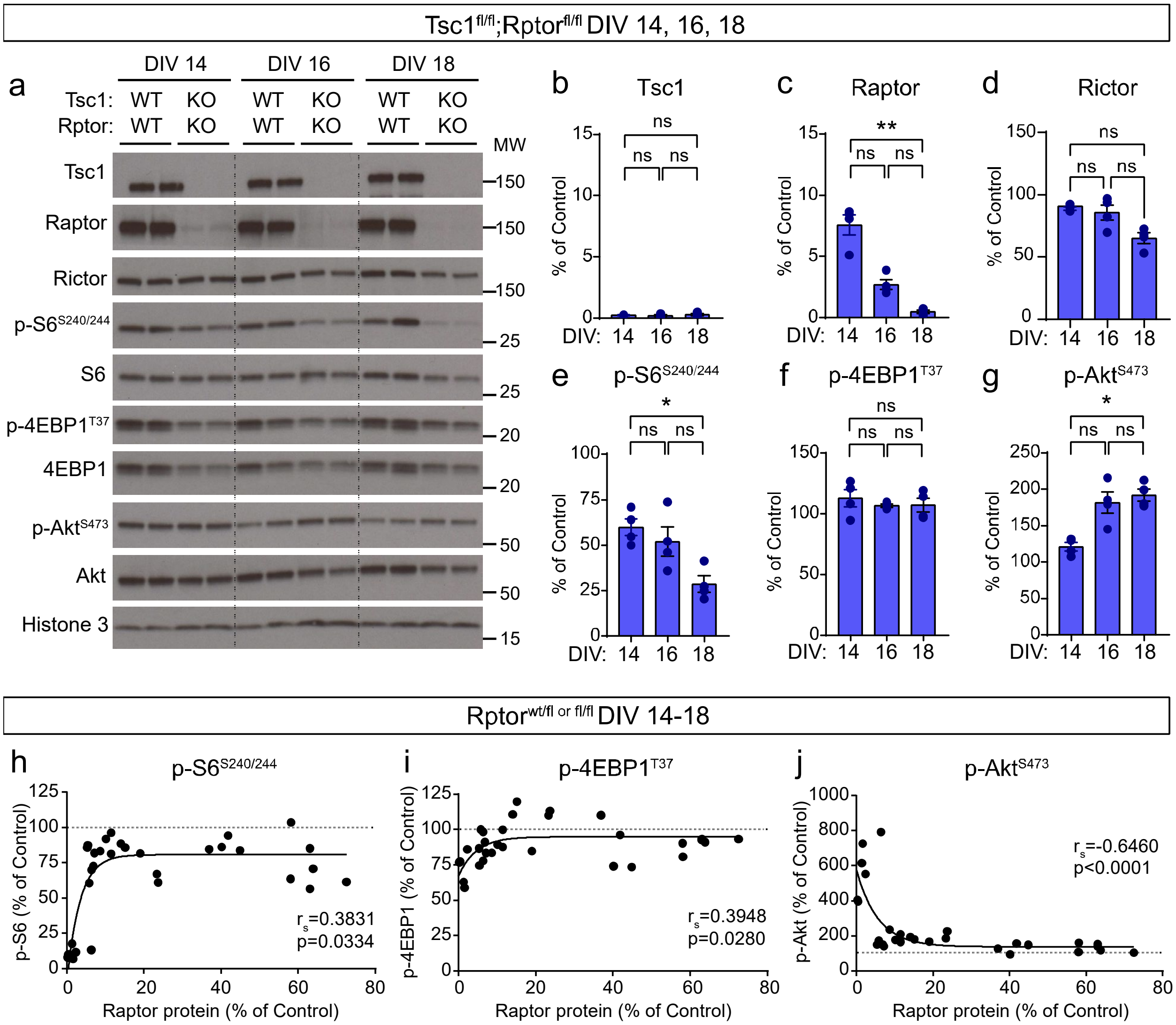

Supplementary Figure 3. Raptor reduction modulates both mTORC1 and mTORC2 signaling (related to Figures 2 and 3).

a) Representative western blots (WB) of lysates from *Tsc1$^{fl/fl}$;Rptor$^{fl/fl}$* hippocampal cultures treated with GFP (WT;WT) or Cre-GFP (KO;KO) on DIV 2 and collected at different time points (DIV 14, 16, and 18). MW indicates molecular weight (in kD). Two samples per genotype are shown. This experiment was replicated two times.

b-g) Bar graphs display WB quantification (mean +/- SEM) for the indicated proteins. Phospho-proteins were normalized to their respective total proteins. The data have been normalized to control values (WT;WT samples) for each DIV. Dots represent data from individual culture wells. For all genotypes n=4 culture wells from 2 independent cultures; 2 mice per culture.

b) Tsc1, Kruskal-Wallis, p=0.5101; D14 vs D16, p>0.9999; D16 vs D18, p=0.7179; D14 vs D18, p>0.9999; Dunn's multiple comparisons tests. ns=non-significant.

c) Raptor, Kruskal-Wallis, p=0.0002; D14 vs D16, p=0.3500; D16 vs D18, p=0.3500; D14 vs D18, **p=0.0051; Dunn's multiple comparisons tests.

d) Rictor, Kruskal-Wallis, p=0.0263; D14 vs D16, p>0.9999; D16 vs D18, p=0.1184; D14 vs D18, p=0.0558 Dunn's multiple comparisons tests.

e) p-S6 Ser240/244, Kruskal-Wallis, p=0.0194; D14 vs D16, p>0.9999; D16 vs D18, p=0.1873; D14 vs D18, *p=0.0324; Dunn's multiple comparisons tests.

f) p-4EBP1 T37, Kruskal-Wallis, p=0.7463; D14 vs D16, p>0.9999; D16 vs D18, p>0.9999; D14 vs D18, p>0.9999; Dunn's multiple comparisons tests.

g) p-Akt Ser473, Kruskal-Wallis, p=0.0132; D14 vs D16, p=0.0723; D16 vs D18, p>0.9999; D14 vs D18, *p=0.0427; Dunn's multiple comparisons tests.

For panels b-g, statistical tests were two-sided and P values were corrected for multiple comparisons.

h-j) Correlation of Raptor protein levels to p-S6 Ser240/244 (h), p-4E-BP1 T37 (i), or p-Akt Ser473 (j) levels within each culture. Phospho-proteins were normalized to their respective total protein levels and expressed as a percentage of control. Samples were pooled across hippocampal cultures from *Rptor*$^{wt/fl\ or\ fl/fl}$ mice treated with AAV-GFP or AAV-Cre-GFP and harvested on different days (DIV 14-18) to generate a range of Raptor protein levels. Solid lines depict non-linear regression. Dashed lines represent control levels. Dots represent individual culture wells, n=31 culture wells. For panel h, r= 0.3831, p=0.0334, two-sided Spearman correlation. For panel i, r=0.3948, p=0.0280, two-sided Spearman correlation. For panel j, r= -0.6460, p<0.0001, two-sided Spearman correlation.

Source data are provided as a Source Data file.

Supplementary Figure 4

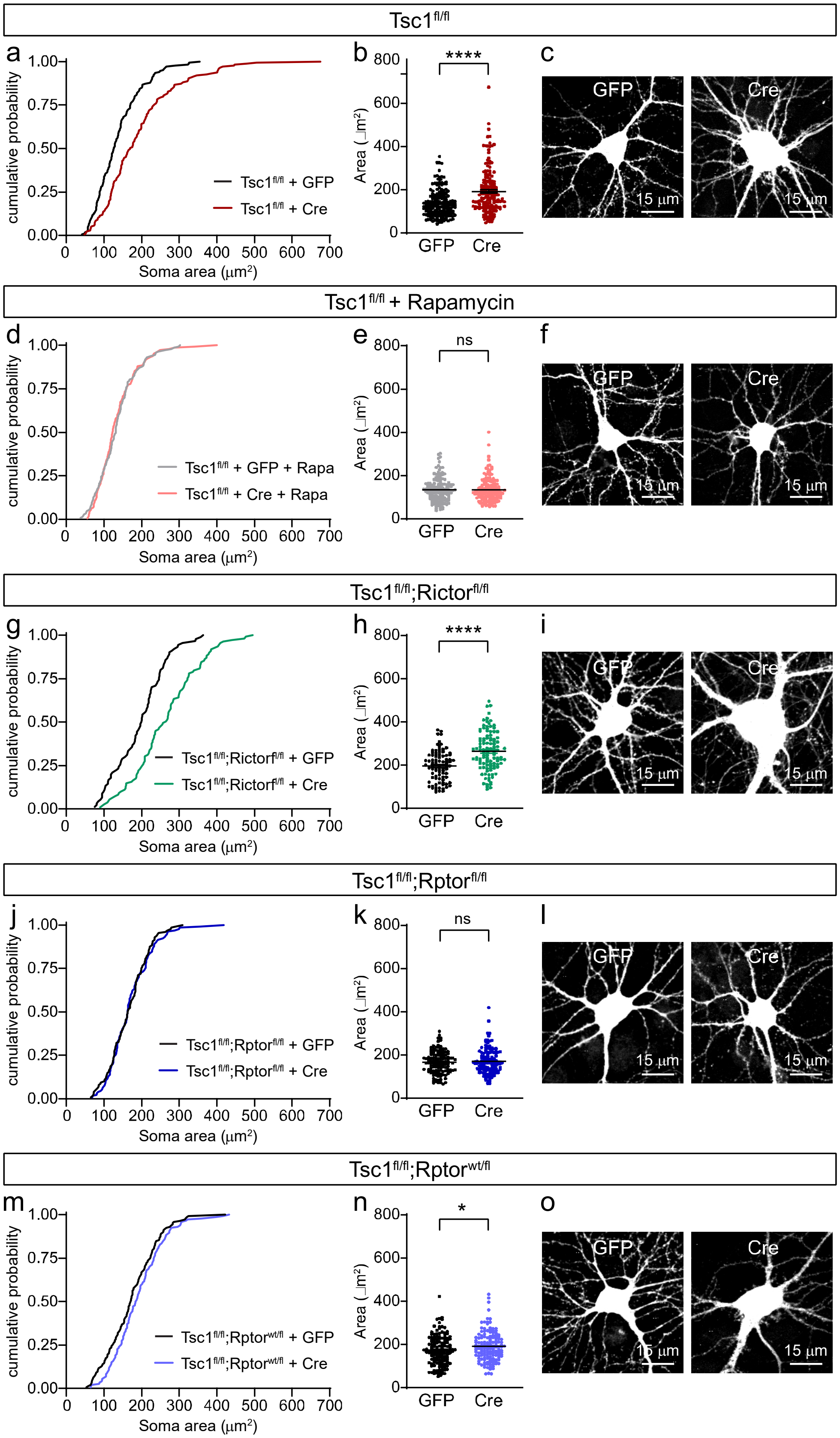

Supplementary Figure 4. Hypertrophy of Tsc1-cKO neurons is prevented by genetic reduction of Raptor but not Rictor (related to Figure 3).

a) Cumulative distributions of soma area for cultured *Tsc1*$^{fl/fl}$ neurons treated with AAV-GFP (Control) or AAV-Cre-mCherry and AAV-Flex-tdTomato (Tsc1-KO). n=182 GFP+ and 175 Cre+ neurons.

b) Scatter dot plot of the data in panel a. Black lines indicated mean +/- SEM. Mann-Whitney, ****p<0.0001. n=182 GFP+ and 175 Cre+ neurons.

c) Example fluorescence images of *Tsc1*$^{fl/fl}$ neurons expressing GFP (Control) or Flex-tdTomato (Tsc1-KO).

d) Cumulative distributions of soma area for cultured *Tsc1*$^{fl/fl}$ neurons treated with AAV-GFP (Control) or AAV-Cre-mCherry and AAV-Flex-tdTomato (Tsc1-KO). Cultures were treated with 50 nM rapamycin from DIV 10-14. n=147 GFP+ and 150 Cre+ neurons.

e) Scatter dot plot of the data in panel d. Black lines indicated mean +/- SEM. Mann-Whitney, p=0.7508. n=147 GFP+ and 150 Cre+ neurons.

f) Example fluorescence images of *Tsc1*$^{fl/fl}$ neurons treated with rapamycin expressing GFP (Control) or Flex-tdTomato (Tsc1-KO).

g) Cumulative distributions of soma area for cultured *Tsc1*$^{fl/fl}$*;Rictor*$^{fl/fl}$ neurons treated with AAV-GFP (Control) or AAV-Cre-mCherry and AAV-Flex-tdTomato (Tsc1-KO;Rictor-KO). n=90 GFP+ and 109 Cre+ neurons.

h) Scatter dot plot of the data in panel g. Black lines indicated mean +/- SEM. Welch's t-test, ****p<0.0001. n=90 GFP+ and 109 Cre+ neurons.

i) Example fluorescence images of *Tsc1*$^{fl/fl}$*;Rictor*$^{fl/fl}$ neurons expressing GFP (Control) or Flex-tdTomato (Tsc1-KO;Rictor-KO).

j) Cumulative distributions of soma area for cultured *Tsc1*$^{fl/fl}$*;Rptor*$^{fl/fl}$ neurons treated with AAV-GFP (Control) or AAV-Cre-mCherry and AAV-Flex-tdTomato (Tsc1-KO;Raptor-KO). n=128 GFP+ and 130 Cre+ neurons.

k) Scatter dot plot of the data in panel j. Black lines indicated mean +/- SEM. Mann-Whitney, p=0.8062. n=128 GFP+ and 130 Cre+ neurons.

l) Example fluorescence images of *Tsc1*$^{fl/fl}$*;Rptor*$^{fl/fl}$ neurons expressing GFP (Control) or Flex-tdTomato (Tsc1-KO;Raptor-KO).

m) Cumulative distributions of soma area for cultured *Tsc1*$^{fl/fl}$*;Rptor*$^{wt/fl}$ neurons treated with AAV-GFP (Control) or AAV-Cre-mCherry and AAV-Flex-tdTomato (Tsc1-KO;Raptor-Het). n=144 GFP+ and 146 Cre+ neurons.

n) Scatter dot plot of the data in panel m. Black lines indicated mean +/- SEM. Mann-Whitney, *p=0.0452. n=144 GFP+ and 146 Cre+ neurons.

o) Example fluorescence images of *Tsc1*$^{fl/fl}$*;Rptor*$^{wt/fl}$ neurons expressing GFP (Control) or Flex-tdTomato (Tsc1-KO;Raptor-Het).

For all panels, cultures were harvested on DIV 14. Neurons were imaged from 4-5 culture wells from 3 independent culture preps, 2 pups per prep. ns=non-significant. Scale bars=15 μm.

All statistical tests were two-sided.

Source data are provided as a Source Data file.

Supplementary Figure 5

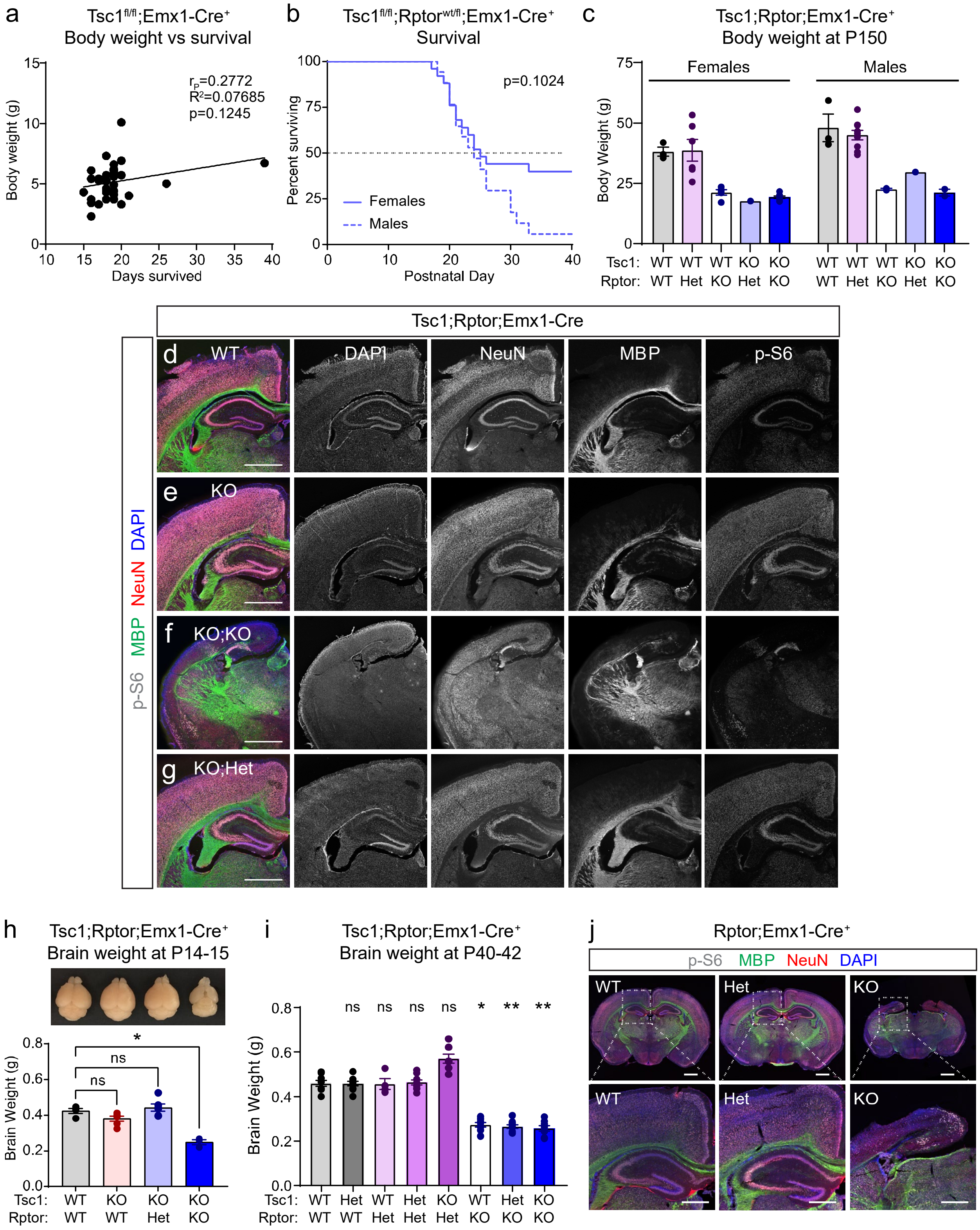

Supplementary Figure 5. Raptor reduction impacts survival, body weight, and forebrain development (related to Figures 4 and 5).

a) Scatterplot displaying the number of days *Tsc1*$^{fl/fl}$*;Emx1-Cre*$^{+}$ (Tsc1-KO) mice survived versus their final recorded body weight. Dots represent individual mice, n=32 mice. Tsc1-KO mice were pooled from the *Tsc1;Rptor;Emx1-Cre* and *Tsc1;Rictor;Emx1-Cre* lines. r= 0.2772, p=0.1245, two-sided Pearson correlation.

b) Survival analysis of *Tsc1*$^{fl/fl}$*;Rptor*$^{wt/fl}$*;Emx1-Cre*$^{+}$ mice by sex. n=17 male and 25 female mice. Log-rank (Mantel-Cox) test, p=0.1024. Dashed black line indicates 50% of the population surviving.

c) Mean +/- SEM body weight of *Tsc1;Rptor;Emx1-Cre* mice of the indicated sex and genotype on postnatal day 150. Dots represent individual mice. n=3 male and 3 female Tsc1-WT;Raptor-WT mice; 9 male and 6 female Tsc1-WT;Raptor-Het mice; 2 male and 5 female Tsc1-WT;Raptor-KO mice; 1 male and 1 female Tsc1-KO;Raptor-Het mouse; and 2 male and 7 female Tsc1-KO;Raptor-KO mice.

d-g) Zoomed-in regions from the panels in Fig. 5a-d. WT=Tsc1-WT;Rptor-WT, KO=Tsc1-KO;Rptor-WT, KO;KO=Tsc1-KO;Rptor-KO, KO;Het=Tsc1-KO;Rptor-Het. Immunostaining for p-S6 Ser240/244 is in grey, myelin basic protein (MBP) is in green, NeuN is in red and DAPI-labeled nuclei are in blue. Scale bars=1 mm. This experiment was replicated 8 times per genotype.

h) Top, representative whole brain images from mice of the genotypes indicated under the respective bar graph. Bottom, mean +/- SEM brain weight from P14-15 mice of the indicated genotypes. Dots represent individual mice. n=4 Tsc1-WT;Raptor-WT mice, 6 Tsc1-KO;Raptor-WT mice, 6 Tsc1-KO;Raptor-Het mice, and 3 Tsc1-KO;Raptor-KO mice. Kruskal-Wallis, p=0.0027; WT;WT vs KO;WT, p=0.5730; WT;WT vs KO;Het, p>0.9999; WT;WT vs KO;KO, *p=0.0371; Dunn's multiple comparison tests. ns=non-significant.

i) Mean +/- SEM brain weight of P40-42 *Tsc1;Rptor;Emx1-Cre* mice of the indicated genotypes. Dots represent individual mice. n=8 Tsc1-WT;Raptor-WT, 10 Tsc1-Het;Raptor-WT, 4 Tsc1-WT;Raptor-Het, 10 Tsc1-Het;Raptor-Het, 7 Tsc1-KO;Raptor-Het, 8 Tsc1-WT;Raptor-KO, 11 Tsc1-Het;Raptor-KO, and 8 Tsc1-KO;Raptor-KO mice. Kruskal-Wallis, p<0.0001. Dunn's multiple comparisons tests to WT: Het;WT p>0.9999, WT;Het p>0.9999, Het;Het p>0.9999, KO;Het p=0.4700, WT;KO *p=0.0258, Het;KO **p=0.0057, KO;KO **p=0.0067.

For panels h and i statistical tests were two-sided and P values were adjusted for multiple comparisons.

j) Representative images of coronal brain sections showing p-S6 Ser240/244 (gray), myelin basic protein (MBP, green), and NeuN (red) immunostaining in P14 *Rptor*$^{wt/wt}$*;Emx1-Cre*$^{+}$ (WT), *Rptor*$^{wt/fl}$*;Emx1-Cre*$^{+}$ (Het) and *Rptor*$^{fl/fl}$*;Emx1-Cre*$^{+}$ (KO) mice (all *Tsc1*$^{wt/wt}$). DAPI staining is in blue. Scale bars=1 mm. Bottom panels show zoomed-in images of the hippocampal regions indicated by the dashed boxes. Scale bars=500 μm. This experiment was replicated three times per genotype.
Source data are provided as a Source Data file.

Supplementary Figure 6

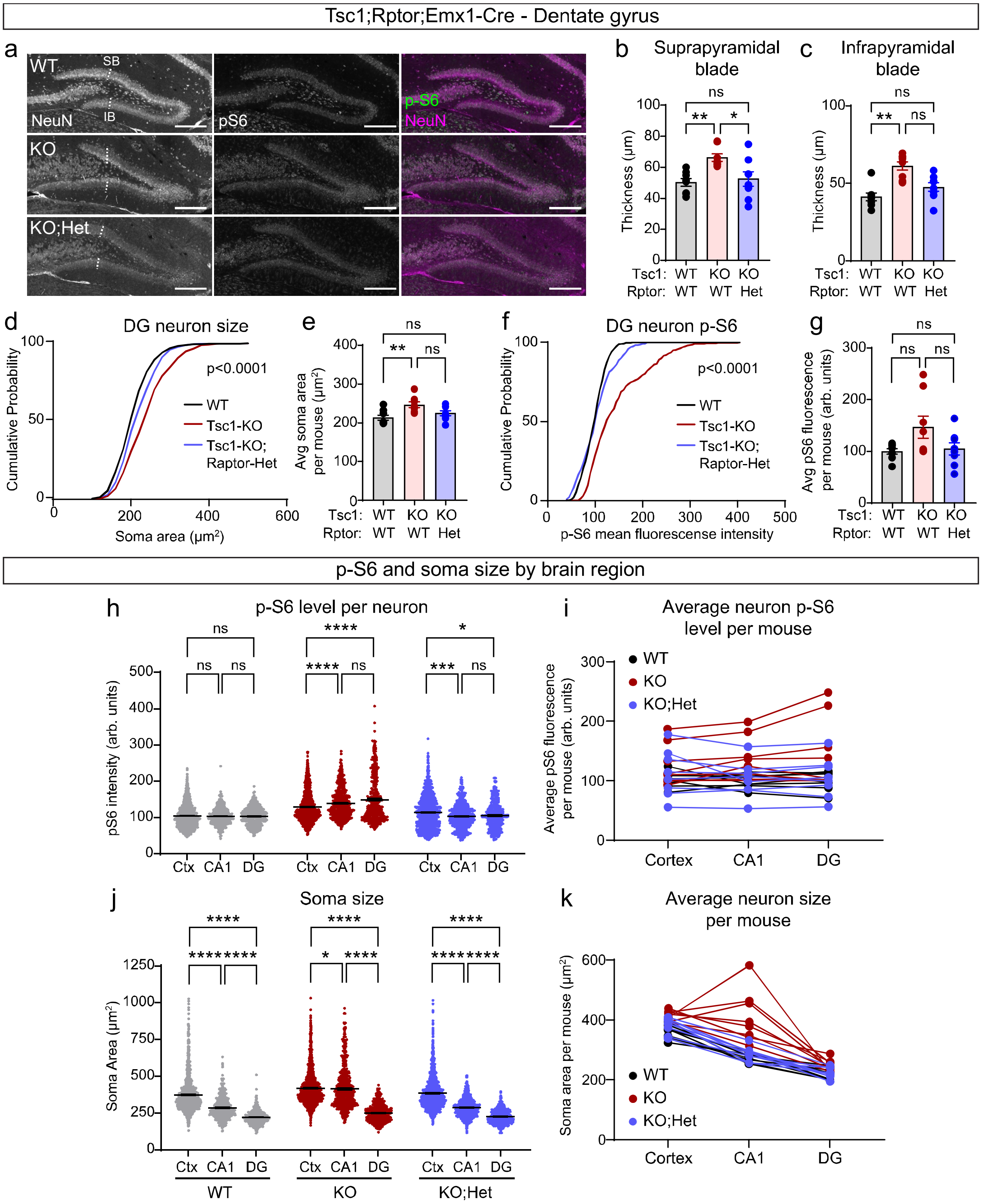

Supplementary Figure 6. p-S6 and soma size exhibit differences by brain region and genotype (related to Figures 5 and 6).

a) Representative images from the dentate gyrus (DG) showing NeuN (left panels) and p-S6 Ser240/244 (middle panels) immunostaining in *Tsc1*$^{wt/wt}$*;Rptor*$^{wt/wt}$*;Emx1-Cre*$^{+}$ (WT), *Tsc1*$^{fl/fl}$*;Rptor*$^{wt/wt}$*;Emx1-Cre*$^{+}$ (KO), and *Tsc1*$^{fl/fl}$*;Rptor*$^{wt/fl}$*;Emx1-Cre*$^{+}$ (KO;Het) mice. Merged images on the right show NeuN in magenta and p-S6 in green. SB=Suprapyramidal blade. IB=Infrapyramidal blade. Lines denote the measurement of IB and SB thickness. Scale bars=250 µm.

b) Mean +/- SEM thickness of the suprapyramidal blade for the indicated genotypes. Dots represent values from individual mice, n=8 mice per genotype. One-way ANOVA, p=0.0055, F(2, 21) = 6.745; WT vs KO, **p=0.0081; WT vs KO;Het, p=0.9473; KO vs KO;Het, *p=0.0253; Sidak's multiple comparisons tests. ns=non-significant.

c) Mean +/- SEM thickness of the infrapyramidal blade for the indicated genotypes. Dots represent values from individual mice, n=8 mice per genotype. Kruskal-Wallis, p=0.0019; WT vs KO, **p=0.0014; WT vs KO;Het, p=0.6093; KO vs KO;Het, p=0.0778; Sidak's multiple comparisons tests.

d) Cumulative distributions of dentate gyrus (DG) neuron soma area for the indicated genotypes. n=403 WT, 401 Tsc1-KO, and 400 Tsc1-KO;Raptor-Het neurons from 8 mice per genotype. Kruskal-Wallis, p<0.0001; WT vs KO, p<0.0001; WT vs KO;Het, p=0.0022; KO vs KO;Het, p<0.0001; Dunn's multiple comparisons tests.

e) Mean +/- SEM DG neuron soma area per mouse for the indicated genotypes. Dots represent values from individual mice, n=8 mice per genotype. One-way ANOVA, p=0.0074, F(2, 21) = 6.257; WT vs KO, **p=0.0067; WT vs KO;Het, p=0.5700; KO vs KO;Het, p=0.0951; Sidak's multiple comparisons tests.

f) Cumulative distributions of DG p-S6 levels per neuron for the indicated genotypes. n is the same as for panel d. Kruskal-Wallis, p<0.0001; WT vs KO, p<0.0001; WT vs KO;Het, p=0.4109; KO vs KO;Het, p<0.0001; Dunn's multiple comparisons tests.

g) Mean +/- SEM DG neuron p-S6 levels per mouse for the indicated genotypes. Dots represent values from individual mice, n=8 mice per genotype. One-way ANOVA, p=0.0591, F (2, 21) = 3.246; WT vs KO, p=0.0883; WT vs KO;Het, p=0.9923; KO vs KO;Het, p=0.1456; Sidak's multiple comparisons tests.

h) Scatter dot plots of p-S6 levels per neuron for the cortex (Ctx), CA1 and DG regions for WT (grey dots), KO (red dots) and KO;Het (blue dots) mice. Black lines indicate mean +/- SEM. n=1601 WT Ctx, 568 WT CA1, and 403 WT DG neurons. n=1605 KO Ctx, 568 KO CA1, and

401 KO DG neurons. n=1602 KO;Het Ctx, 561 KO;Het CA1, and 400 KO;Het DG neurons from 8 mice per genotype. WT p-S6 levels: Kruskal-Wallis test, p=0.0669; Ctx vs CA1, p=0.1806; Ctx vs DG, p=0.2338; CA1 vs DG, p>0.9999; Dunn's multiple comparison tests. KO p-S6 levels: Kruskal-Wallis test, p<0.0001; Ctx vs CA1, ****p<0.0001; Ctx vs DG, ****p<0.0001; CA1 vs DG, p>0.9999; Dunn's multiple comparison tests. KO;Het p-S6 levels: Kruskal-Wallis test, p=0.0001; Ctx vs CA1, ***p=0.0002, Ctx vs DG, *p=0.0485, CA1 vs DG, p>0.9999, Dunn's multiple comparison tests.

i) Average p-S6 levels per mouse for neurons in the cortex, CA1 and DG regions for WT (black dots), KO (red dots) and KO;Het (blue dots) mice. Lines connect data points from the same mouse. n=8 mice per genotype.

j) Scatter dot plots of soma area for neurons in the Ctx, CA1 and DG regions for WT (grey dots), KO (red dots) and KO;Het (blue dots) mice. n is the same as in panel h. WT soma area: Kruskal-Wallis test, p<0.0001; Ctx vs CA1, ****p<0.0001; Ctx vs DG, ****p<0.0001, CA1 vs DG, ****p<0.0001; Dunn's multiple comparison tests. KO soma area: Kruskal-Wallis test, p<0.0001; Ctx vs CA1, *p=0.0299, Ctx vs DG, ****p<0.0001; CA1 vs DG, ****p<0.0001, Dunn's multiple comparison tests. KO;Het soma area: Kruskal-Wallis test, p<0.0001; Ctx vs CA1, ****p<0.0001; Ctx vs DG, ****p<0.0001, CA1 vs DG, ****p<0.0001, Dunn's multiple comparison tests.

k) Average soma size per mouse for neurons in the cortex, CA1 and DG regions for WT (black dots), KO (red dots) and KO;Het (blue dots) mice. Lines connect data points from the same mouse. n=8 mice per genotype.

Statistical tests were two-sided and P values were adjusted for multiple comparisons.

Source data are provided as a Source Data file.

Supplementary Figure 7

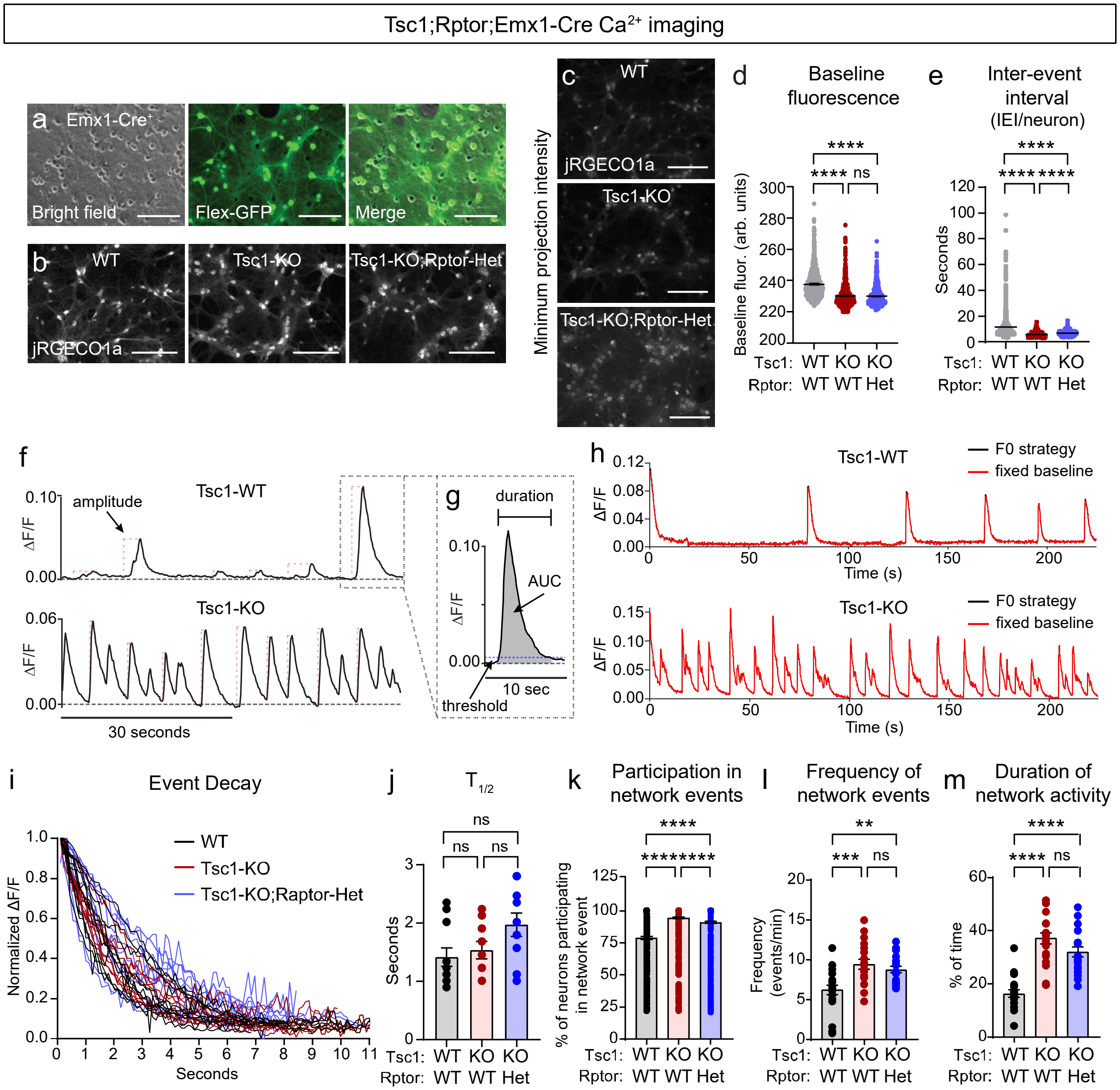

Supplementary Figure 7. Additional calcium imaging analysis for *Tsc1;Rptor;Emx1-Cre* hippocampal cultures (related to Figure 7).

a) Representative images of a primary hippocampal culture from a *Tsc1*$^{wt/wt}$*;Emx1-Cre*$^{+}$ mouse transduced with a Cre-dependent GFP expressing virus (AAV-Flex-GFP). Bright field (left panel), GFP fluorescence (middle panel) and merged (right panel) images are shown. Scale bars=150 μm. This experiment was replicated three times.

b) Representative images of cultures from *Tsc1$^{wt/wt}$;Rptor$^{wt/wt}$;Emx1-Cre^{+}* (WT), *Tsc1$^{fl/fl}$;Rptor$^{wt/wt}$;Emx1-Cre^{+}* (KO), and *Tsc1$^{fl/fl}$;Rptor$^{wt/fl}$;Emx1-Cre^{+}* (Tsc1-KO;Rptor-Het) mice expressing jRGeco1a. Scale bars=250 µm. This experiment was replicated 20 times per genotype.

c) Representative minimum intensity projection images from WT, Tsc1-KO, and Tsc1-KO;Rptor-Het cultures expressing jRGeco1a. Scale bars=250 µm. This experiment was replicated 20 times per genotype.

d) Scatter dot plot of the jRGeco1a baseline fluorescence intensity in neurons from the indicated genotypes. Black lines indicate mean +/- SEM. n=800 neurons from 20 culture wells per genotype from 6 WT and 7 KO and KO;Het independent culture preps, 1 pup per prep. Kruskal-Wallis, p<0.0001; WT vs KO, ****p<0.0001; WT vs KO;Het, ****p<0.0001; KO vs KO;Het, p>0.9999. ns=non-significant.

e) Scatter dot plot of the average inter-evet interval (IEI) per neuron for the indicated genotypes. Black lines indicate the mean +/- SEM. n=800 neurons from 20 culture wells per genotype from 6 WT and 7 KO and KO;Het independent culture preps, 1 pup per prep. Kruskal-Wallis, p<0.0001; WT vs KO, ****p<0.0001; WT vs KO;Het, ****p<0.0001; KO vs KO;Het, ****p<0.0001; Dunn's multiple comparison tests.

f) Example Ca^{2+} imaging analysis showing individual Ca^{2+} transients from a Tsc1-WT (top) and Tsc1-KO (bottom) neuron. Vertical red dashed lines indicate amplitude measurements. See Methods for additional details.

g) Example analysis of a single Ca^{2+} transient showing the duration, area under the curve (AUC, grey shaded region) and threshold measurements. Crossing of the threshold defines event initiation and termination.

h) Comparison of two methods for calculating ΔF/F. The top panel shows example traces from a Tsc1-WT neuron and the bottom panel shows examples from a Tsc1-KO neuron. The black traces use the standard approach of calculating ΔF/F by normalizing to each cell's measured F0. Since we observed some differences in baseline fluorescence between the genotypes, we also tested a strategy in which we calculated ΔF/F by normalizing to a fixed baseline (red traces). The traces are largely overlapping demonstrating that the ΔF/F calculation is similar with both strategies. The F0 method was used for all Ca^{2+} imaging analysis as described in the Methods.

i) Representative Ca^{2+} transients fitted with a double exponential decay function from the indicated genotypes. The first transient was analyzed from n=11 WT, 10 Tsc1-KO, and 10 Tsc1-KO;Rptor-Het neurons randomly selected from independent movies.

j) Mean +/- SEM time to decay to ½ of their starting value ($T_{1/2}$) for the representative transients in panel i. n=11 WT, 10 Tsc1-KO, and 10 Tsc1-KO;Rptor-Het transients from independent neurons. One-way ANOVA, p=0.0683, F (2, 28) = 2.958; WT vs Tsc1-KO, p=0.9457; WT vs Tsc1-KO;Rptor-Het, p= 0.0793; Tsc1-KO vs Tsc1-KO;Rptor-Het, p=0.2324; Sidak's multiple comparisons tests.

k) Mean +/- SEM percentage of neurons in a field of view that participated in each network event for the indicated genotypes. Each dot represents a network event. n=469 WT, 707 Tsc1-KO, and 656 Tsc1-KO;Rptor-Het network events from 20 individual culture wells per genotype. Culture wells were from 6-7 independent preps, 1 pup per prep. Kruskal-Wallis, p<0.0001; WT vs KO, ****p<0.0001; WT vs KO;Het, ****p<0.0001; KO vs KO;Het, ****p<0.0001; Dunn's multiple comparisons tests.

l) Mean +/- SEM frequency of network events per culture for the indicated genotypes. Each dot represents a single culture well. n=20 individual culture wells per genotype, from 6-7 independent preps, 1 pup per prep. One-way ANOVA, p=0.0002, F (2, 57) = 9.773; WT vs KO, ***p=0.0003; WT vs KO;Het, **p=0.0058; KO vs KO;Het, p=0.7602; Sidak's multiple comparisons tests.

m) Mean +/- SEM duration of network activity, expressed as the percentage of the recording time during which network events occurred for the indicated genotypes. Each dot represents a single culture well. n=20 individual culture wells per genotype, from 6-7 independent preps, 1 pup per prep. Kruskal-Wallis, p<0.0001; WT vs KO, ****p<0.0001; WT vs KO;Het, ****p<0.0001; KO vs KO;Het, p=0.5496; Dunn's multiple comparisons tests.

Statistical tests were two-sided and P values were adjusted for multiple comparisons.

Source data are provided as a Source Data file.

Supplementary Figure 8

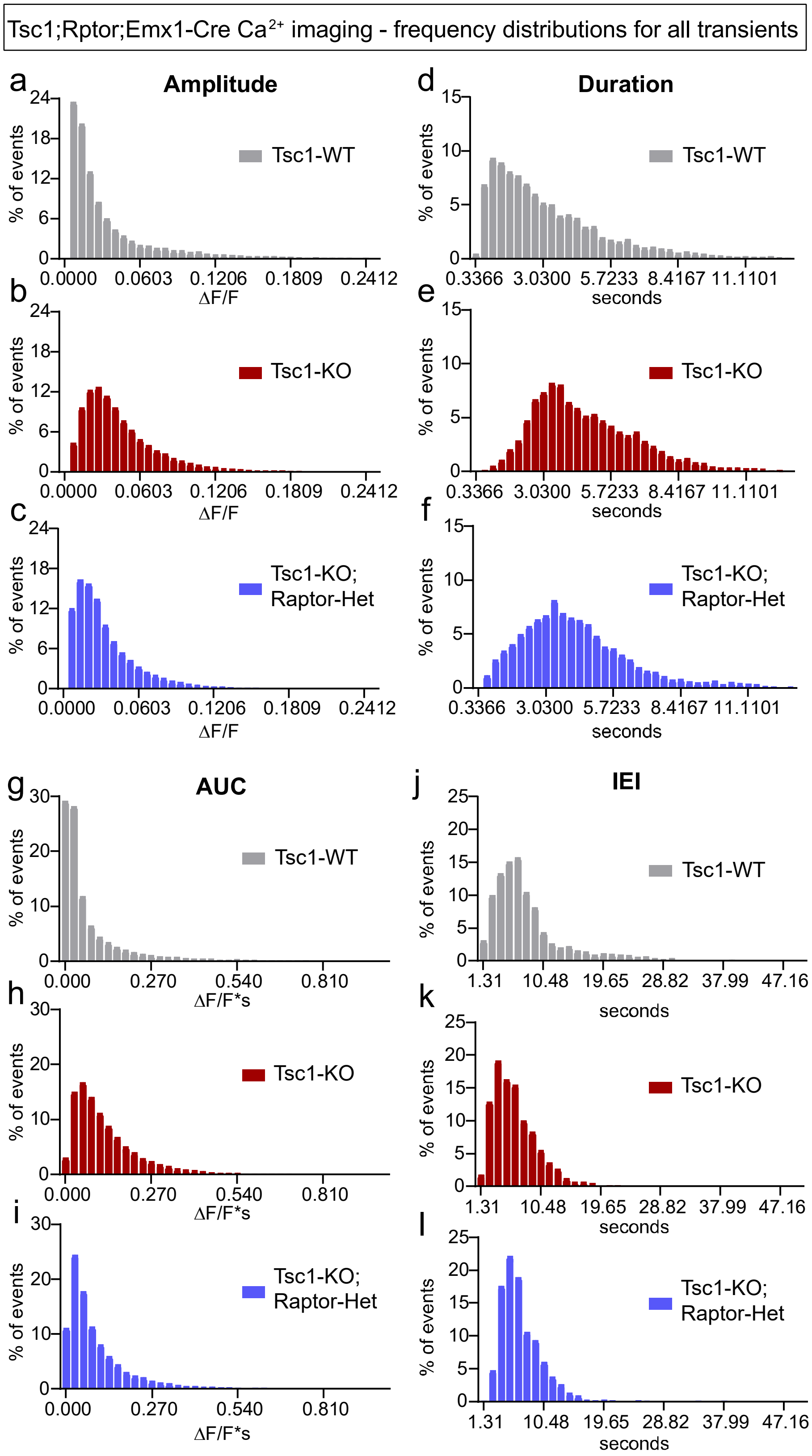

Supplementary Figure. 8. Frequency distribution histograms for *Tsc1;Rptor;Emx1-Cre* calcium imaging analysis (related to Figure 7).

a-c) Frequency distribution histograms of Ca^{2+} transient amplitudes for events detected in WT (a), Tsc1-KO (b), and Tsc1-KO;Raptor-Het (c) neurons. n=19,109 WT, 26,574 KO, and 24,649 KO;Het transients. Only transients with amplitudes <0.25 ∆F/F were included in this analysis.

d-f) Frequency distribution histograms of Ca^{2+} transient duration for events detected in WT (d), Tsc1-KO (e), and Tsc1-KO;Raptor-Het (f) neurons. n=18,998 WT, 26,487 KO, and 24,531 KO;Het transients. Only transients with durations <13 seconds were included in this analysis.

g-i) Frequency distribution histograms of Ca^{2+} transient area under the curve (AUC) for events detected in WT (g), Tsc1-KO (h), and Tsc1-KO;Raptor-Het (i) neurons. n=18,490 WT, 25,796 KO, and 24,230 KO;Het transients. Only transients with AUC <1.0 ∆F/F*s were included in this analysis.

j-l) Frequency distribution histograms of Ca^{2+} transient inter-event interval (IEI) for events detected in WT (j), Tsc1-KO (k), and Tsc1-KO;Raptor-Het (l) neurons. n=18,297 WT, 25,809 KO, and 23,840 KO;Het transients. Only transients with IEI <50 seconds were included in this analysis.

Source data are provided as a Source Data file.

Supplementary Figure 9

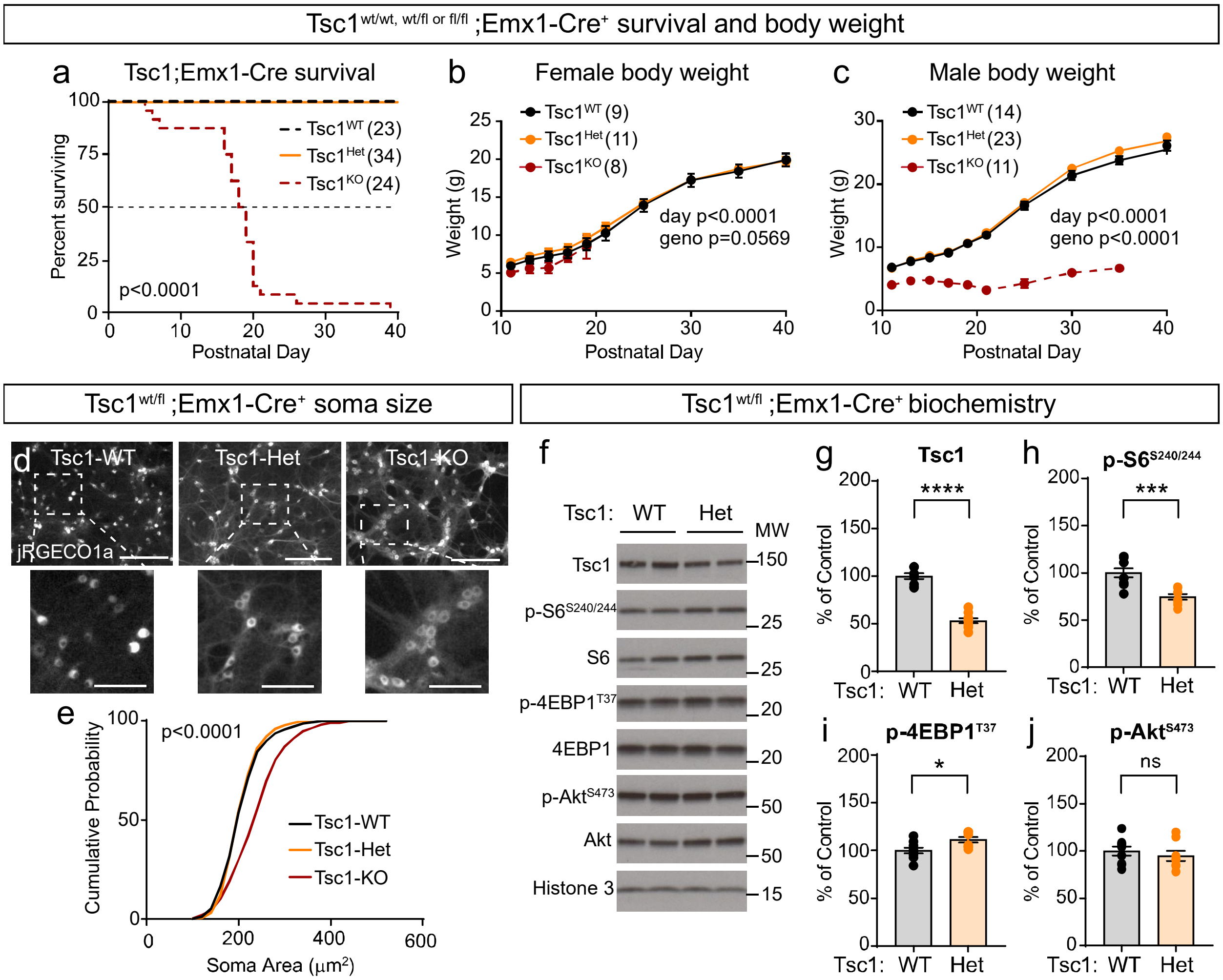

Supplementary Figure 9. Heterozygous deletion of *Tsc1* does not affect survival, body weight, or soma size (related to Figures 1, 3 and 4).

a) Survival analysis of $Tsc1^{wt/wt}$;$Rptor^{wt/wt}$;*Emx1*-Cre^{+} ($Tsc1^{WT}$), $Tsc1^{wt/fl}$;$Rptor^{wt/wt}$;*Emx1*-Cre^{+} ($Tsc1^{Het}$), and $Tsc1^{fl/fl}$;$Rptor^{wt/wt}$;*Emx1*-Cre^{+} ($Tsc1^{KO}$) mice. Data for $Tsc1^{WT}$ and $Tsc1^{KO}$ mice are re-plotted from Fig. 4c for reference (dashed lines). The number of mice for each genotype is indicated in parentheses. Dashed line indicates 50% of the population surviving. P value from Log-rank Mantel-Cox tests is shown.

b,c) Mean +/- SEM body weight in grams measured from postnatal day 11 to 40 for female (b) and male (c) mice of the indicated genotypes. Data for $Tsc1^{WT}$ and $Tsc1^{KO}$ mice are re-plotted from Fig. 4e,f for reference (dashed lines). The number of mice for each genotype is indicated in parentheses. Mixed-effects model (REML) with Geisser-Greenhouse correction statistics:

Females (b), day p<0.0001, F (1.199, 25.84) = 1056; geno p=0.0569, F (2, 25) = 3.221. Males (c), day p<0.0001, F (1.792, 71.09) = 1113; geno p<0.0001, F (2, 44) = 29.07. Multiple comparisons testing was not performed.

d) Representative images of *Tsc1*$^{wt/wt}$*;Emx1*-Cre^{+} (WT), *Tsc1*$^{wt/fl}$*;Emx1*-Cre^{+} (Tsc1-Het) and *Tsc1*$^{fl/fl}$*;Emx1*-Cre^{+} (Tsc1-KO) primary hippocampal cultures expressing jRGeco1a on DIV 14. This experiment was replicated 5 times. Scale bars for top panels=250 µm. Scale bars for zoomed-in images (bottom panels)=100 µm.

e) Cumulative distributions of soma area for WT, Tsc1-Het and Tsc1-KO cultured hippocampal neurons measured from jRGECO1a fluorescence. n=454 WT, 451 Tsc1-Het and 453 Tsc1-KO neurons from 5 independent culture preps, 1 pup per prep. Kruskal-Wallis test, p<0.0001; WT vs Het, p>0.9999; WT vs KO, ****p<0.0001; Het vs KO, ****p<0.0001; Dunn's multiple comparison tests.

f) Representative western blots of lysates collected from *Tsc1*$^{wt/wt}$*;Emx1*-Cre^{+} (WT) and *Tsc1*$^{wt/fl}$*;Emx1*-Cre^{+} (Het) primary hippocampal cultures. MW indicates molecular weight. Two independent samples per genotype are shown. This experiment was replicated three times.

g-j) Bar graphs display western blot quantification (mean +/- SEM) for Tsc1 (g) Welch's test, ****p<0.0001; p-S6 Ser240/244 (h) Welch's test, ***p= 0.0005; p-4EBP1 T37 (i) Mann-Whitney test, *p= 0.0152; and p-Akt Ser473 (j) Welch's test, p= 0.4734. Phospho-proteins were normalized to their respective total proteins and expressed as a percent of WT. Dots represent data from individual culture wells. n=9 WT and 8 Tsc1-Het culture wells from 3 independent culture preps, 1 pup per prep.

Statistical tests were two-sided and P values were adjusted for multiple comparisons.

Source data are provided as a Source Data file.

Supplementary Figure 10

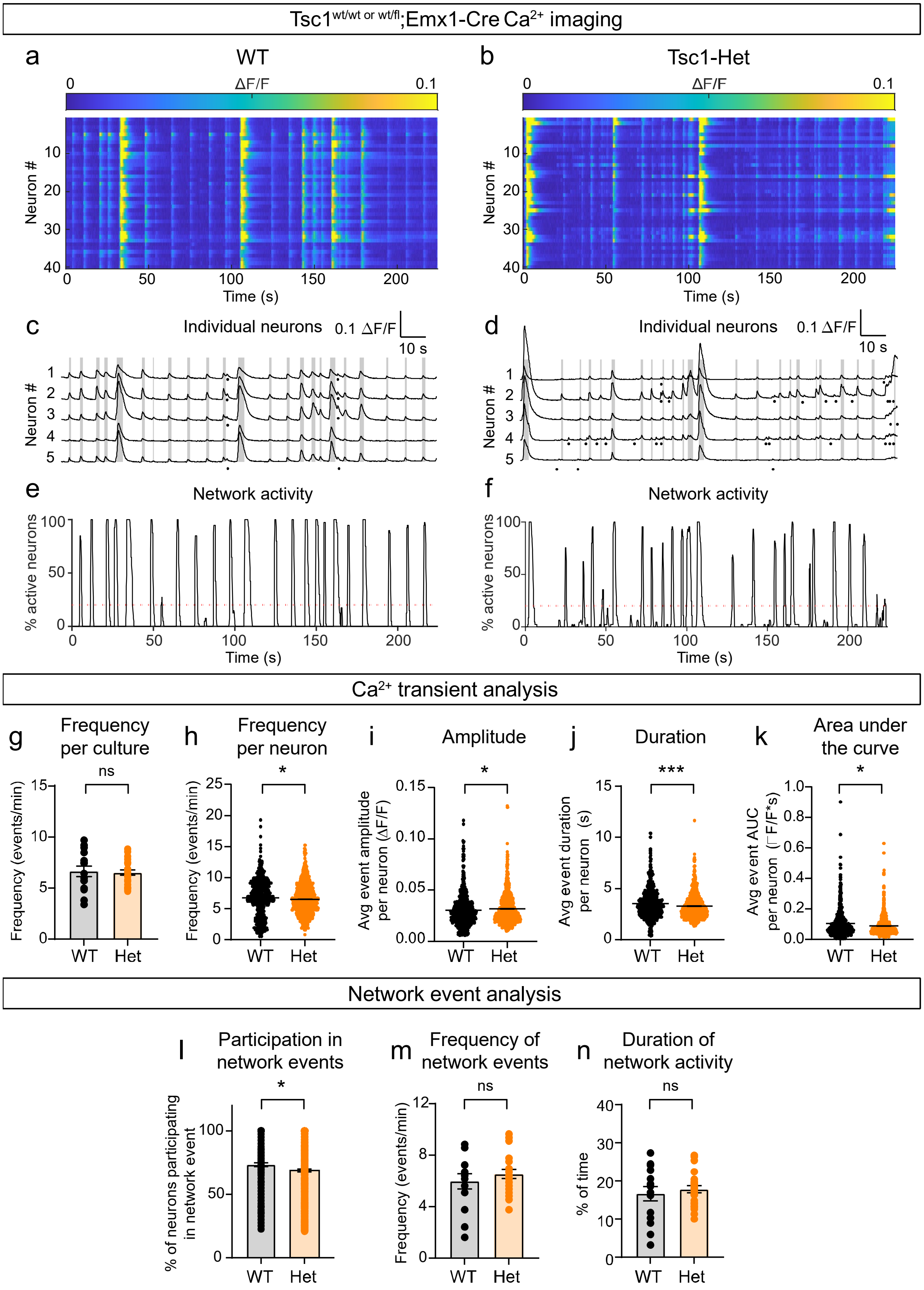

Supplementary Figure 10. Loss of one copy of *Tsc1* does not induce neuronal or network hyperexcitability in hippocampal cultures (related to Figure 7).

a,b) Representative heatmaps of ΔF/F for 40 neurons imaged in a field of view from *Tsc1*$^{wt/wt}$;*Emx1*-Cre^{+} (WT) and *Tsc1*$^{wt/fl}$;*Emx1*-Cre^{+} (Tsc1-Het) cultures.

c,d) Ca^{2+} transients from 5 representative neurons imaged in a field of view from WT (c) and Tsc1-Het (d) cultures. Grey lines indicate network events with more than 20% of neurons in the field of view active at the same time. Black dots represent spontaneous Ca^{2+} transients that were not part of network events.

e,f) Graphs show the percentage of neurons in the field of view that were active at a given time for WT (e) and Tsc1-Het (f) cultures. One representative culture is shown per genotype. Red dashed lines at 20% indicate the threshold for a network event.

g) Mean +/- SEM Ca^{2+} transient frequency per culture. Dots represent values from individual cultures. For WT: n=15 individual culture wells from 5 independent culture preps, 1 pup per prep. For Tsc1-Het: n=24 individual culture wells from 9 independent culture preps, 1 pup per prep. Mann-Whitney test, p=0.7002; ns=non-significant.

h) Scatter dot plot of the Ca^{2+} transient frequency per neuron for the indicated genotypes. Black lines indicate mean +/- SEM. For WT: n=600 neurons from 15 individual culture wells from 5 independent culture preps, 1 pup per prep. For Tsc1-Het: n=960 neurons from 24 individual culture wells from 9 independent culture preps, 1 pup per prep. Mann-Whitney test, *p=0.0136.

i) Scatter dot plot of the average Ca^{2+} transient amplitude per neuron for the indicated genotypes. n is the same as for panel h. Mann-Whitney test, *p=0.0287.

j) Scatter dot plot of the average Ca^{2+} transient duration per neuron for the indicated genotypes. n is the same as for panel h. Mann-Whitney test, ***p=0.0003.

k) Scatter dot plot of the average Ca^{2+} transient area under the curve (AUC) per neuron for the indicated genotypes. n is the same as for panel h. Mann-Whitney test, *p=0.0384.

l) Mean +/- SEM percentage of neurons in a field of view that participated in each network event for the indicated genotypes. Each dot represents a network event. For WT: n=334 network events from 15 individual culture wells from 5 independent culture preps, 1 pup per prep. For Tsc1-Het: n=585 network events from 24 individual culture wells from 9 independent culture preps, 1 pup per prep. Mann-Whitney test, *p=0.0361.

m) Mean +/- SEM frequency of network events per culture for the indicated genotypes. Each dot represents a single culture well. For WT: n=15 individual culture wells from 5 independent culture preps. For Tsc1-Het: n=24 individual culture wells from 9 independent culture preps; 1 pup per prep. Welch's test, p=0.4185.

n) Mean +/- SEM duration of network activity, expressed as the percentage of the recording time during which network events occurred for the indicated genotypes. Each dot represents a single culture well. n is the same as for panel m. Welch's test, p=0.6057.

Statistical tests were two-sided.

Source data are provided as a Source Data file.

Supplementary Figure 11

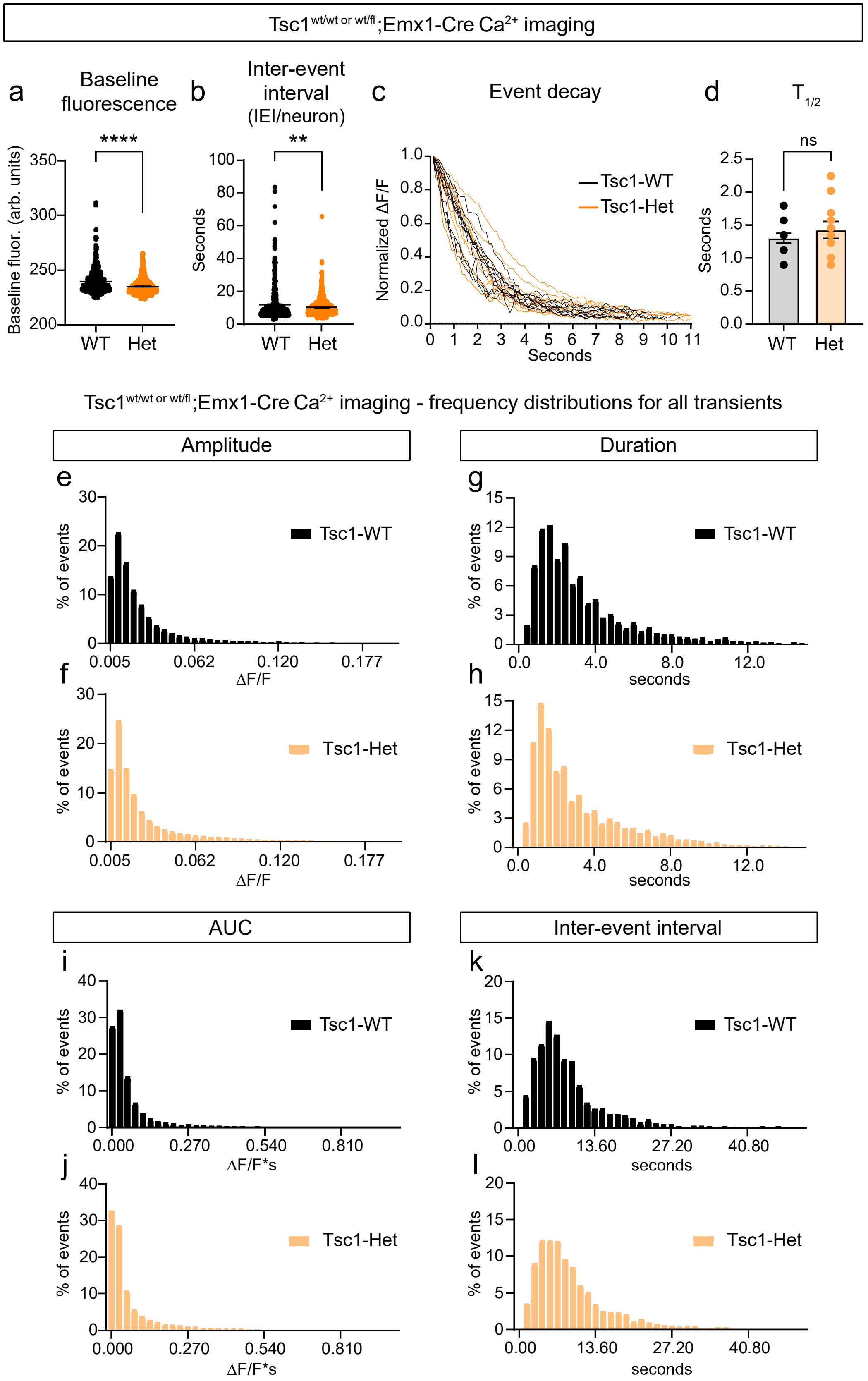

Supplementary Figure 11. Additional calcium imaging analysis for *Tsc1$^{wt/wt\ or\ wt/fl}$;Emx1-Cre* hippocampal cultures (related to Figure 7).

a) Scatter dot plot of the jRGeco1a baseline fluorescence intensity in neurons from the indicated genotypes. Black lines indicate mean +/- SEM. For WT: n=600 neurons from 15 individual culture wells from 5 independent culture preps, 1 pup per prep. For Tsc1-Het: n=960 neurons from 24 individual culture wells from 9 independent culture preps, 1 pup per prep. Mann-Whitney test, ****p<0.0001.

b) Scatter dot plot of the average inter-evet interval (IEI) per neuron for the indicated genotypes. Black lines indicate the mean +/- SEM. n is the same as for panel a. Mann-Whitney test, **p=0.0076

c) Representative Ca^{2+} transients from Tsc1-WT (black) or Tsc1-Het (orange) neurons fitted with a double exponential decay function. The first transient from 11 randomly selected neurons per genotype (from independent movies) are displayed.

d) Mean +/- SEM time to decay to half of their starting value ($T_{1/2}$) for the representative transients in panel c. n=11 transients from independent neurons per genotype. Dots represent values for individual transients. Mann-Whitney test, p=0.6599. ns=non-significant.

e,f) Frequency distribution histograms of Ca^{2+} transient amplitudes for events detected in WT (e) and Tsc1-Het (f) neurons. n=13,026 WT and 20,235 Tsc1-Het transients. Only transients with amplitudes <0.20 ΔF/F were included in this analysis.

g,h) Frequency distribution histograms of Ca^{2+} transient duration for events detected in WT (g) and Tsc1-Het (h) neurons. n=13,003 WT and 20,267 Tsc1-Het transients. Only transients with durations <15 seconds were included in this analysis.

i,j) Frequency distribution histograms of Ca^{2+} transient area under the curve (AUC) for events detected in WT (i) and Tsc1-Het (j) neurons. n=12,578 WT and 19,686 Tsc1-Het transients. Only transients with AUC <1.0 ΔF/F*s were included in this analysis.

k,l) Frequency distribution histograms of Ca^{2+} transient inter-event interval (IEI) for events detected in WT (k) and Tsc1-Het (l) neurons. n=12,446 WT and 20,126 Tsc1-Het. Only transients with IEI <50 seconds were included in this analysis.

Statistical tests were two-sided.

Source data are provided as a Source Data file.

Supplementary Figure 12

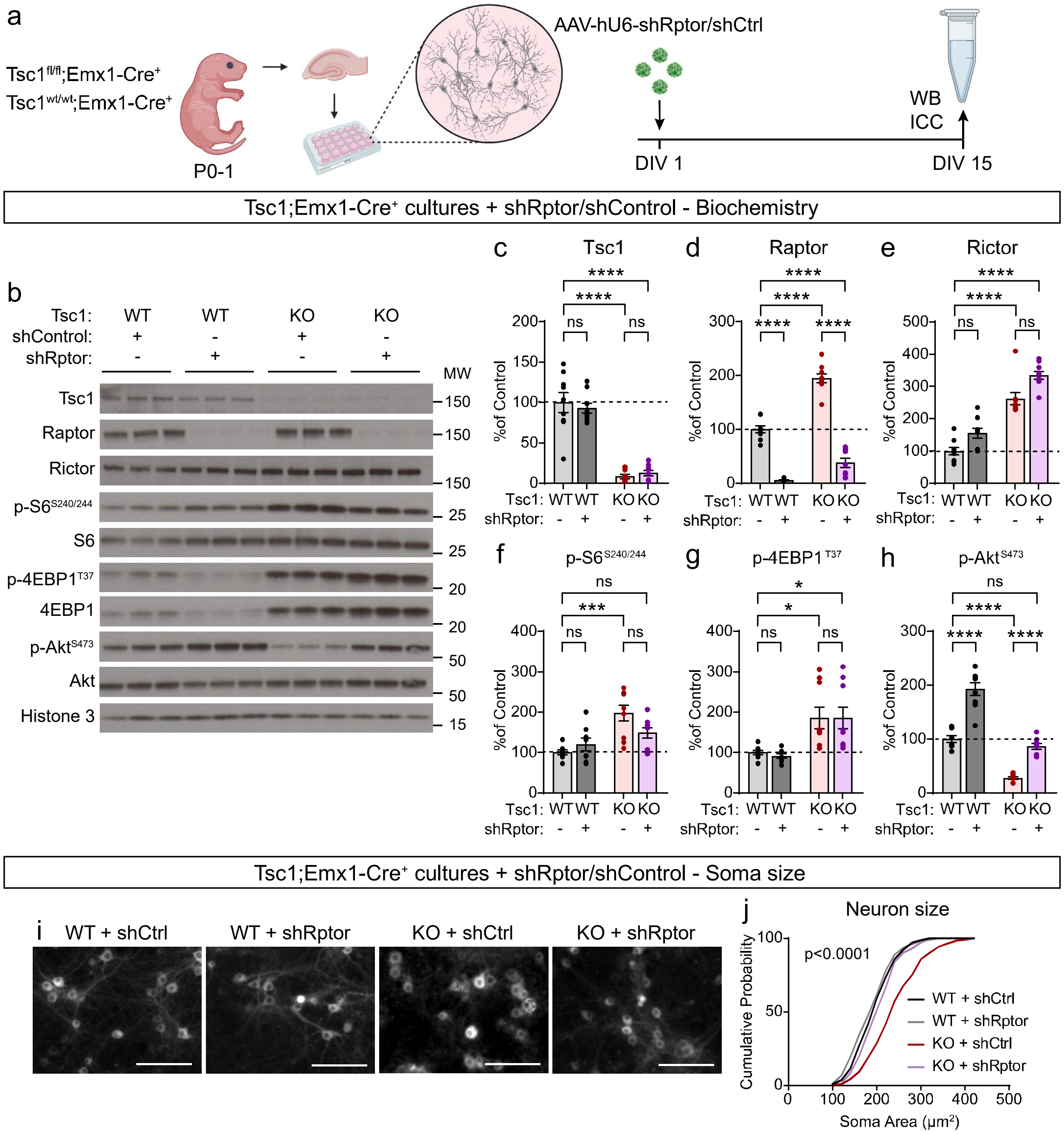

Supplementary Figure 12. shRptor rebalances mTOR signaling and rescues neuronal hypertrophy in cultured Tsc1-KO neurons (related to Figure 8).

a) Schematic of the experiment. Primary hippocampal cultures were prepared from P0-1 *Tsc1$^{wt/wt}$;Emx1-Cre^{+}* (WT) and *Tsc1$^{fl/fl}$;Emx1-Cre^{+}* (Tsc1-KO) mice and transduced with AAV-shControl-EYFP (shControl) or AAV-shRptor-EYFP (shRptor) on DIV 1. Cells were collected for

analysis by western blot (WB) or immunocytochemistry (ICC) on DIV 15. Created with BioRender.com

b) Representative WB of lysates collected from WT and Tsc1-KO primary hippocampal cultures treated with shControl or shRptor. MW indicates molecular weight. Three samples per genotype and treatment are shown. This experiment was replicated three times.

c-h) Bar graphs display WB quantification (mean +/- SEM) for the indicated proteins, expressed as a percentage of control (WT + shControl) levels. Phospho-proteins were normalized to their respective total proteins. Dots represent data from individual culture wells. n=8 culture wells for p-S6 and 4-EBP1 and 9 culture wells for all other proteins, from 3 independent culture preps, 1 pup per prep.

c) Tsc1, One-way ANOVA, p<0.0001, F (3, 32) = 48.58; WT+shControl vs WT+shRptor, p= 0.9360; WT+shControl vs KO+shControl, ****p<0.0001; WT+shControl vs KO+shRptor, ****p<0.0001; KO+shControl vs KO+shRptor, p=0.9918; Sidak's multiple comparison tests. ns=non-significant.

d) Raptor, One-way ANOVA, p<0.0001, F (3, 32) = 153.6; WT+shControl vs WT+shRptor, ****p<0.0001; WT+shControl vs KO+shControl, ****p<0.0001; WT+shControl vs KO+shRptor, ****p<0.0001; KO+shControl vs KO+shRptor, ****p<0.0001; Sidak's multiple comparison tests.

e) Rictor, Kruskal-Wallis, p<0.0001; WT+shControl vs WT+shRptor, p=0.8411; WT+shControl vs KO+shControl, **p=0.0023; WT+shControl vs KO+shRptor, ****p<0.0001; KO+shControl vs KO+shRptor, p=0.5592; Dunn's multiple comparison tests.

f) p-S6 Ser240/244, One-way ANOVA, p=0.0004, F (3, 30) = 8.234; WT+shControl vs WT+shRptor, p=0.8388; WT+shControl vs KO+shControl, ***p=0.0003; WT+shControl vs KO+shRptor, p=0.1045; KO+shControl vs KO+shRptor, p=0.0894; Sidak's multiple comparison tests.

g) p-4EBP1 T37, One-way ANOVA, p=0.0015, F (3, 30) = 6.582; WT+shControl vs WT+shRptor, p=0.9973; WT+shControl vs KO+shControl, *p=0.0224; WT+shControl vs KO+shRptor,* p=0.0220; KO+shControl vs KO+shRptor, p>0.9999; Sidak's multiple comparison tests.

h) p-Akt Ser473, One-way ANOVA, p<0.0001, F (3, 32) = 92.30; WT+shControl vs WT+shRptor, ****p<0.0001; WT+shControl vs KO+shControl, ****p<0.0001; WT+shControl vs KO+shRptor, p=0.5561; KO+shControl vs KO+shRptor, ****p<0.0001; Sidak's multiple comparison tests.

i) Representative images of DIV 15 WT and Tsc1-KO primary hippocampal cultures transduced with shControl or shRptor together with jRGeco1a to visualize cell bodies. Scale bars=100 μm.

j) Cumulative distributions of soma area for WT and Tsc1-KO cultured hippocampal neurons

treated with shControl or shRptor. n=246 WT+shControl, 249 WT+shRptor, 245 Tsc1-KO+shControl and 246 Tsc1-KO+shRptor neurons from 8 culture wells from 4 independent culture preps, 1 pup per prep. Kruskal-Wallis, p<0.0001; WT+shControl vs WT+shRptor, p= 0.4789; WT+shControl vs KO+shControl, p<0.0001; WT+shControl vs KO+shRptor, p=0.2352; KO+shControl vs KO+shRptor, p<0.0001; Dunn's multiple comparison tests.
Statistical tests were two-sided and P values were adjusted for multiple comparisons.
Source data are provided as a Source Data file.

Supplementary Figure 13

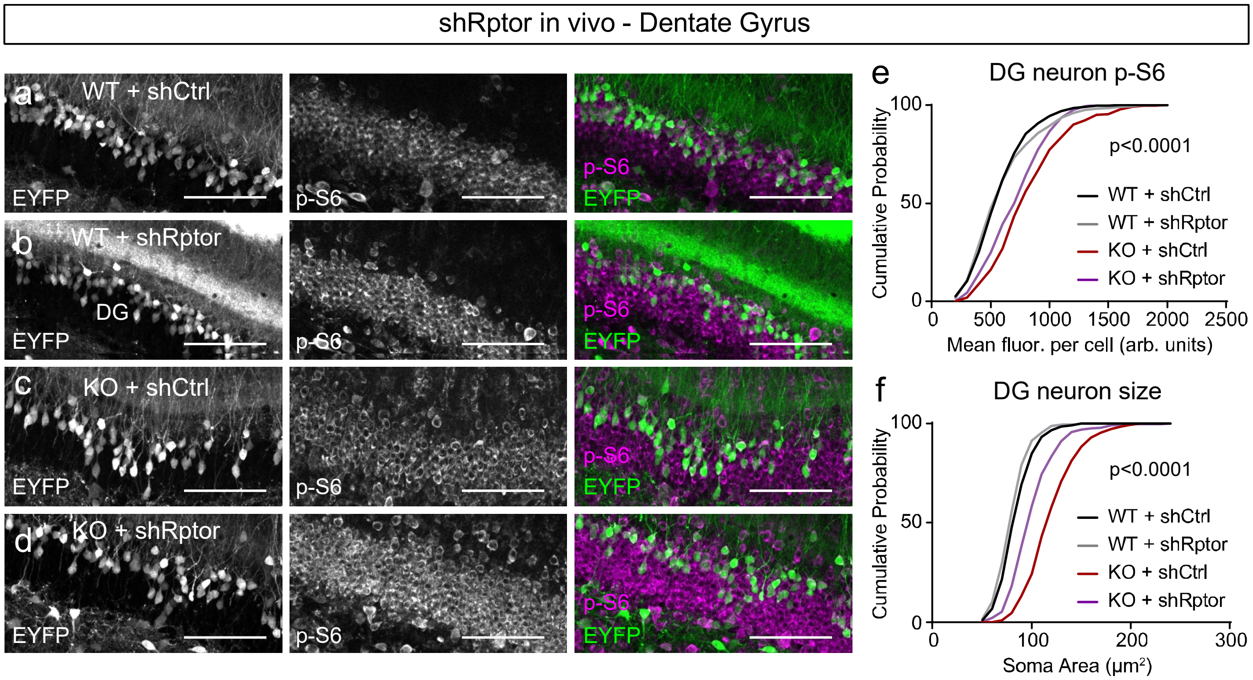

Supplementary Figure 13. shRptor improves cellular phenotypes in the dentate gyrus of Tsc1-cKO mice (related to Figure 8).

a-d) Representative images of the dentate gyrus (DG) of *Tsc1*$^{wt/wt}$*;Emx1-Cre*$^{+}$ (WT, a-b) and *Tsc1*$^{fl/fl}$*;Emx1-Cre*$^{+}$ (KO, c-d) mice injected with AAV-shRptor-EYFP or AAV-shCtrl-EYFP virus showing EYFP fluorescence (left panels) and p-S6 240/244 immunostaining (middle panels). Right panels show merged images. Scale bars=100 μm.

e) Cumulative distributions of p-S6 levels in DG EYFP+ neurons for the indicated genotypes. n=396 WT+shCtrl, 396 WT+shRptor, 382 Tsc1-KO+shCtrl and 350 Tsc1-KO+shRptor neurons from 6 mice per group. Kruskal-Wallis, p<0.0001; WT+shCtrl vs WT+shRptor, p>0.9999; WT+shCtrl vs Tsc1-KO+shCtrl, p<0.0001; WT+shCtrl vs Tsc1-KO+shRptor, p<0.0001; Tsc1-KO+shCtrl vs Tsc1-KO+shRptor, p=0.0026; Dunn's multiple comparison tests.

f) Cumulative distributions of EYFP+ DG neuron soma area for the indicated genotypes. n is the same as in panel e. Kruskal-Wallis, p<0.0001; WT+shCtrl vs WT+shRptor, p=0.0241; WT+shCtrl vs Tsc1-KO+shCtrl, p<0.0001; WT+shCtrl vs Tsc1-KO+shRptor, p<0.0001; Tsc1-KO+shCtrl vs Tsc1-KO+shRptor, p<0.0001; Dunn's multiple comparison tests.

Statistical tests were two-sided and P values were adjusted for multiple comparisons.

Source data are provided as a Source Data file.

Supplementary Table 1. Survival analysis of *Tsc1;Rptor;Emx1-Cre*, *Tsc1;Rictor;Emx1-Cre* and *Tsc1;Emx1-Cre* + AAV9-shRNA mice.

Mouse line	Genotype	Sex	Day of first mortality (P0-P40)[1]	Median survival in days (P0-P40)[1]	Age of oldest surviving animal (days)[2]
Tsc1;Rptor;Emx1-Cre	*Tsc1*$^{wt/wt}$;*Rptor*$^{wt/wt}$;*Emx1-Cre*$^{+}$	M	n/a	n/a	>150
Tsc1;Rptor;Emx1-Cre	*Tsc1*$^{wt/wt}$;*Rptor*$^{wt/wt}$;*Emx1-Cre*$^{wt/+}$	F	n/a	n/a	>150
Tsc1;Rptor;Emx1-Cre	*Tsc1*$^{wt/wt}$;*Rptor*$^{wt/fl}$;*Emx1-Cre*$^{+}$	M	n/a	n/a	>150
Tsc1;Rptor;Emx1-Cre	*Tsc1*$^{wt/wt}$;*Rptor*$^{wt/fl}$;*Emx1-Cre*$^{+}$	F	n/a	n/a	>150
Tsc1;Rptor;Emx1-Cre	*Tsc1*$^{wt/wt}$;*Rptor*$^{fl/fl}$;*Emx1-Cre*$^{wt/+}$	M	n/a	n/a	>150
Tsc1;Rptor;Emx1-Cre	*Tsc1*$^{wt/wt}$;*Rptor*$^{fl/fl}$;*Emx1-Cre*$^{+}$	F	n/a	n/a	>150
Tsc1;Rptor;Emx1-Cre	*Tsc1*$^{wt/fl}$;*Rptor*$^{wt/wt}$;*Emx1-Cre*$^{+}$	M	n/a	n/a	>150
Tsc1;Rptor;Emx1-Cre	*Tsc1*$^{wt/fl}$;*Rptor*$^{wt/wt}$;*Emx1-Cre*$^{+}$	F	n/a	n/a	>150
Tsc1;Rptor;Emx1-Cre	*Tsc1*$^{wt/fl}$;*Rptor*$^{wt/fl}$;*Emx1-Cre*$^{+}$	M	n/a	n/a	>150
Tsc1;Rptor;Emx1-Cre	*Tsc1*$^{wt/fl}$;*Rptor*$^{wt/fl}$;*Emx1-Cre*$^{+}$	F	n/a	n/a	>150
Tsc1;Rptor;Emx1-Cre	*Tsc1*$^{wt/fl}$;*Rptor*$^{fl/fl}$;*Emx1-Cre*$^{+}$	M	n/a	n/a	>150
Tsc1;Rptor;Emx1-Cre	*Tsc1*$^{wt/fl}$;*Rptor*$^{fl/fl}$;*Emx1-Cre*$^{+}$	F	n/a	n/a	>150
Tsc1;Rptor;Emx1-Cre	*Tsc1*$^{fl/fl}$;*Rptor*$^{wt/wt}$;*Emx1-Cre*$^{+}$	M[3]	16	19	39
Tsc1;Rptor;Emx1-Cre	*Tsc1*$^{fl/fl}$;*Rptor*$^{wt/wt}$;*Emx1-Cre*$^{+}$	F[3]	16	18.5	20
Tsc1;Rptor;Emx1-Cre	*Tsc1*$^{fl/fl}$;*Rptor*$^{wt/fl}$;*Emx1-Cre*$^{+}$	M	18	24	>150
Tsc1;Rptor;Emx1-Cre	*Tsc1*$^{fl/fl}$;*Rptor*$^{wt/fl}$;*Emx1-Cre*$^{+}$	F	17	25	>150
Tsc1;Rptor;Emx1-Cre	*Tsc1*$^{fl/fl}$;*Rptor*$^{fl/fl}$;*Emx1-Cre*$^{+}$	M	n/a	n/a	>150
Tsc1;Rptor;Emx1-Cre	*Tsc1*$^{fl/fl}$;*Rptor*$^{fl/fl}$;*Emx1-Cre*$^{+}$	F	30	n/a[4]	>150

Tsc1;Rictor;Emx1-Cre	*$Tsc1^{wt/wt}$;$Rictor^{wt/wt}$;Emx1-Cre^{+}*	M	n/a	n/a	-
Tsc1;Rictor;Emx1-Cre	*$Tsc1^{wt/wt}$;$Rictor^{wt/wt}$;Emx1-Cre^{+}*	F	n/a	n/a	-
Tsc1;Rictor;Emx1-Cre	*$Tsc1^{wt/wt}$;$Rictor^{wt/fl}$;Emx1-Cre^{+}*	M	n/a	n/a	-
Tsc1;Rictor;Emx1-Cre	*$Tsc1^{wt/wt}$;$Rictor^{wt/fl}$;Emx1-Cre^{+}*	F	n/a	n/a	-
Tsc1;Rictor;Emx1-Cre	*$Tsc1^{wt/wt}$;$Rictor^{fl/fl}$;Emx1-Cre^{+}*	M	n/a	n/a	-
Tsc1;Rictor;Emx1-Cre	*$Tsc1^{wt/wt}$;$Rictor^{fl/fl}$;Emx1-Cre^{+}*	F	n/a	n/a	-
Tsc1;Rictor;Emx1-Cre	*$Tsc1^{wt/fl}$;$Rictor^{wt/wt}$;Emx1-Cre^{+}*	M	n/a	n/a	-
Tsc1;Rictor;Emx1-Cre	*$Tsc1^{wt/fl}$;$Rictor^{wt/wt}$;Emx1-Cre^{+}*	F	n/a	n/a	-
Tsc1;Rictor;Emx1-Cre	*$Tsc1^{wt/fl}$;$Rictor^{wt/fl}$;Emx1-Cre^{+}*	M	n/a	n/a	-
Tsc1;Rictor;Emx1-Cre	*$Tsc1^{wt/fl}$;$Rictor^{wt/fl}$;Emx1-Cre^{+}*	F	n/a	n/a	-
Tsc1;Rictor;Emx1-Cre	*$Tsc1^{wt/fl}$;$Rictor^{fl/fl}$;Emx1-Cre^{+}*	M	n/a	n/a	-
Tsc1;Rictor;Emx1-Cre	*$Tsc1^{wt/fl}$;$Rictor^{fl/fl}$;Emx1-Cre^{+}*	F	n/a	n/a	-
Tsc1;Rictor;Emx1-Cre	*$Tsc1^{fl/fl}$;$Rictor^{wt/wt}$;Emx1-Cre^{+}*	M	15	19	19
Tsc1;Rictor;Emx1-Cre	*$Tsc1^{fl/fl}$;$Rictor^{wt/wt}$;Emx1-Cre^{+}*	F	17	18	18
Tsc1;Rictor;Emx1-Cre	*$Tsc1^{fl/fl}$;$Rictor^{wt/fl}$;Emx1-Cre^{+}*	M	14	18	21
Tsc1;Rictor;Emx1-Cre	*$Tsc1^{fl/fl}$;$Rictor^{wt/fl}$;Emx1-Cre^{+}*	F	14	17	21
Tsc1;Rictor;Emx1-Cre	*$Tsc1^{fl/fl}$;$Rictor^{fl/fl}$;Emx1-Cre^{+}*	M	18	19.5	22
Tsc1;Rictor;Emx1-Cre	*$Tsc1^{fl/fl}$;$Rictor^{fl/fl}$;Emx1-Cre^{+}*	F	18	20	24
Tsc1;Emx1-Cre[5]	*$Tsc1^{wt/wt}$;Emx1-Cre^{+}* + AAV9-shControl	M	n/a	n/a	>150
Tsc1;Emx1-Cre[5]	*$Tsc1^{wt/wt}$;Emx1-Cre^{+}* + AAV9-shControl	F	n/a	n/a	>150
Tsc1;Emx1-Cre[5]	*$Tsc1^{fl/fl}$;Emx1-Cre^{+}* + AAV9-shControl	M	14	21	40

Tsc1;Emx1-Cre[5]	*Tsc1*$^{fl/fl}$*;Emx1-Cre*$^{+}$ + AAV9-shControl	F	10	17	20
Tsc1;Emx1-Cre[5]	*Tsc1*$^{wt/wt}$*;Emx1-Cre*$^{+}$ + AAV9-shRaptor	M	n/a	n/a	>150
Tsc1;Emx1-Cre[5]	*Tsc1*$^{wt/wt}$*;Emx1-Cre*$^{+}$ + AAV9-shRaptor	F	n/a	n/a	>150
Tsc1;Emx1-Cre[5]	*Tsc1*$^{fl/fl}$*;Emx1-Cre*$^{+}$ + AAV9-shRaptor	M	17	21.5	48
Tsc1;Emx1-Cre[5]	*Tsc1*$^{fl/fl}$*;Emx1-Cre*$^{+}$ + AAV9-shRaptor	F	20	22	51

Footnotes:

[1] n/a = No animals died within the first 40 postnatal days

[2] - = Animals were not monitored past the first 40 postnatal days

[3] Three *Tsc1*$^{fl/fl}$*;Rptor*$^{wt/wt}$*;Emx1-Cre*$^{+}$ mice were found dead before P10 and sex could not be determined.

[4] One *Tsc1*$^{fl/fl}$*;Rptor*$^{fl/fl}$*;Emx1-Cre*$^{+}$ female died at P30.

[5] Survival for this line was monitored after P10 and wet food was provided for the pups on the floor of the cage starting at P14.

Source data are provided as a Source Data file.

Supplementary Table 2. Body weight of *Tsc1;Rptor;Emx1-Cre*,*Tsc1;Rictor;Emx1-Cre* and *Tsc1;Emx1-Cre* + AAV9-shRNA mice.

Mouse line	Genotype	Sex	Mean +/- SEM body weight at P15 (n)	Mean +/- SEM body weight at P150 (n)[1,2]
Tsc1;Rptor;Emx1-Cre	*Tsc1^wt/wt^;Rptor^wt/wt^;Emx1-Cre^+^*	M	8.42 +/- 0.31 (14)	48.00 +/- 5.69 (3)
Tsc1;Rptor;Emx1-Cre	*Tsc1^wt/wt^;Rptor^wt/wt^;Emx1-Cre^+^*	F	7.56 +/- 0.57 (9)	38.17 +/- 1.92 (3)
Tsc1;Rptor;Emx1-Cre	*Tsc1^wt/wt^;Rptor^wt/fl^;Emx1-Cre^+^*	M	8.38 +/- 0.32 (14)	45.06 +/- 1.99 (9)
Tsc1;Rptor;Emx1-Cre	*Tsc1^wt/wt^;Rptor^wt/fl^;Emx1-Cre^+^*	F	7.90 +/- 0.40 (13)	38.73 +/- 4.52 (6)
Tsc1;Rptor;Emx1-Cre	*Tsc1^wt/wt^;Rptor^fl/fl^;Emx1-Cre^+^*	M	6.26 +/- 0.36 (7)	22.50 +/- 0.40 (2)
Tsc1;Rptor;Emx1-Cre	*Tsc1^wt/wt^;Rptor^fl/fl^;Emx1-Cre^+^*	F	6.28 +/- 0.48 (10)	21.18 +/- 1.12 (5)
Tsc1;Rptor;Emx1-Cre	*Tsc1^wt/fl^;Rptor^wt/wt^;Emx1-Cre^+^*	M	8.64 +/- 0.30 (23)	46.60 +/- 1.50 (6)
Tsc1;Rptor;Emx1-Cre	*Tsc1^wt/fl^;Rptor^wt/wt^;Emx1-Cre^+^*	F	7.78 +/- 0.51 (11)	44.20 +/- 1.10 (2)
Tsc1;Rptor;Emx1-Cre	*Tsc1^wt/fl^;Rptor^wt/fl^;Emx1-Cre^+^*	M	8.03 +/- 0.29 (38)	43.05 +/- 1.52 (16)
Tsc1;Rptor;Emx1-Cre	*Tsc1^wt/fl^;Rptor^wt/fl^;Emx1-Cre^+^*	F	8.44 +/- 0.23 (39)	36.47 +/- 3.01 (11)
Tsc1;Rptor;Emx1-Cre	*Tsc1^wt/fl^;Rptor^fl/fl^;Emx1-Cre^+^*	M	6.72 +/- 0.42 (17)	24.50 +/- 1.17 (3)
Tsc1;Rptor;Emx1-Cre	*Tsc1^wt/fl^;Rptor^fl/fl^;Emx1-Cre^+^*	F	5.64 +/- 0.88 (9)	20.05 +/- 1.45 (2)
Tsc1;Rptor;Emx1-Cre	*Tsc1^fl/fl^;Rptor^wt/wt^;Emx1-Cre^+^*	M	4.81 +/- 0.34 (11)	n/a
Tsc1;Rptor;Emx1-Cre	*Tsc1^fl/fl^;Rptor^wt/wt^;Emx1-Cre^+^*	F	5.55 +/- 0.63 (8)	n/a
Tsc1;Rptor;Emx1-Cre	*Tsc1^fl/fl^;Rptor^wt/fl^;Emx1-Cre^+^*	M	7.00 +/- 0.50 (16)	29.60 (1)
Tsc1;Rptor;Emx1-Cre	*Tsc1^fl/fl^;Rptor^wt/fl^;Emx1-Cre^+^*	F	6.65 +/- 0.29 (23)	17.60 (1)
Tsc1;Rptor;Emx1-Cre	*Tsc1^fl/fl^;Rptor^fl/fl^;Emx1-Cre^+^*	M	5.68 +/- 0.55 (8)	21.15 +/- 1.45 (2)
Tsc1;Rptor;Emx1-Cre	*Tsc1^fl/fl^;Rptor^fl/fl^;Emx1-Cre^+^*	F	6.80 +/- 0.32 (9)	19.29 +/- 0.51 (7)

Tsc1;Rictor;Emx1-Cre	*$Tsc1^{wt/wt}$;$Rictor^{wt/wt}$;Emx1-Cre^{+}*	M	9.42 +/- 0.24 (12)	-
Tsc1;Rictor;Emx1-Cre	*$Tsc1^{wt/wt}$;$Rictor^{wt/wt}$;Emx1-Cre^{+}*	F	8.78 +/- 0.28 (12)	-
Tsc1;Rictor;Emx1-Cre	*$Tsc1^{wt/wt}$;$Rictor^{wt/fl}$;Emx1-Cre^{+}*	M	9.63 +/- 0.22 (6)	-
Tsc1;Rictor;Emx1-Cre	*$Tsc1^{wt/wt}$;$Rictor^{wt/fl}$;Emx1-Cre^{+}*	F	9.45 +/- 0.81 (6)	-
Tsc1;Rictor;Emx1-Cre	*$Tsc1^{wt/wt}$;$Rictor^{fl/fl}$;Emx1-Cre^{+}*	M	7.55 +/- 0.45 (6)	-
Tsc1;Rictor;Emx1-Cre	*$Tsc1^{wt/wt}$;$Rictor^{fl/fl}$;Emx1-Cre^{+}*	F	9.07 +/- 0.69 (3)	-
Tsc1;Rictor;Emx1-Cre	*$Tsc1^{wt/fl}$;$Rictor^{wt/wt}$;Emx1-Cre^{+}*	M	9.38 +/- 0.42 (19)	-
Tsc1;Rictor;Emx1-Cre	*$Tsc1^{wt/fl}$;$Rictor^{wt/wt}$;Emx1-Cre^{+}*	F	8.76 +/- 0.47 (14)	-
Tsc1;Rictor;Emx1-Cre	*$Tsc1^{wt/fl}$;$Rictor^{wt/fl}$;Emx1-Cre^{+}*	M	9.54 +/- 0.27 (27)	-
Tsc1;Rictor;Emx1-Cre	*$Tsc1^{wt/fl}$;$Rictor^{wt/fl}$;Emx1-Cre^{+}*	F	9.48 +/- 0.35 (30)	-
Tsc1;Rictor;Emx1-Cre	*$Tsc1^{wt/fl}$;$Rictor^{fl/fl}$;Emx1-Cre^{+}*	M	8.56 +/- 0.33 (24)	-
Tsc1;Rictor;Emx1-Cre	*$Tsc1^{wt/fl}$;$Rictor^{fl/fl}$;Emx1-Cre^{+}*	F	8.07 +/-0.32 (16)	-
Tsc1;Rictor;Emx1-Cre	*$Tsc1^{fl/fl}$;$Rictor^{wt/wt}$;Emx1-Cre^{+}*	M	5.31 +/- 0.54 (8)	n/a
Tsc1;Rictor;Emx1-Cre	*$Tsc1^{fl/fl}$;$Rictor^{wt/wt}$;Emx1-Cre^{+}*	F	5.60 +/- 0.39 (4)	n/a
Tsc1;Rictor;Emx1-Cre	*$Tsc1^{fl/fl}$;$Rictor^{wt/fl}$;Emx1-Cre^{+}*	M	5.82 +/- 0.59 (13)	n/a
Tsc1;Rictor;Emx1-Cre	*$Tsc1^{fl/fl}$;$Rictor^{wt/fl}$;Emx1-Cre^{+}*	F	4.81 +/- 0.38 (10)	n/a
Tsc1;Rictor;Emx1-Cre	*$Tsc1^{fl/fl}$;$Rictor^{fl/fl}$;Emx1-Cre^{+}*	M	5.91 +/- 0.34 (10)	n/a
Tsc1;Rictor;Emx1-Cre	*$Tsc1^{fl/fl}$;$Rictor^{fl/fl}$;Emx1-Cre^{+}*	F	4.76 +/- 0.34 (5)	n/a
Tsc1;Emx1-Cre[3]	*$Tsc1^{wt/wt}$;Emx1-Cre^{+}* + AAV9-shControl	M	8.31 +/- 0.38 (11)	-
Tsc1;Emx1-Cre	*$Tsc1^{wt/wt}$;Emx1-Cre^{+}* + AAV9-shControl	F	7.80 +/- 0.33 (14)	-
Tsc1;Emx1-Cre	*$Tsc1^{fl/fl}$;Emx1-Cre^{+}* + AAV9-shControl	M	5.51 +/- 0.25 (13)	n/a
Tsc1;Emx1-Cre	*$Tsc1^{fl/fl}$;Emx1-Cre^{+}* + AAV9-shControl	F	5.68 +/- 0.79 (4)	n/a

Tsc1;Emx1-Cre	*Tsc1*$^{wt/wt}$*;Emx1-Cre*$^{+}$ + AAV9-shRaptor	M	7.45 +/- 0.31 (13)	-
Tsc1;Emx1-Cre	*Tsc1*$^{wt/wt}$*;Emx1-Cre*$^{+}$ + AAV9-shRaptor	F	7.34 +/- 0.32 (12)	-
Tsc1;Emx1-Cre	*Tsc1*$^{fl/fl}$*;Emx1-Cre*$^{+}$ + AAV9-shRaptor	M	7.17 +/- 0.26 (15)	-
Tsc1;Emx1-Cre	*Tsc1*$^{fl/fl}$*;Emx1-Cre*$^{+}$ + AAV9-shRaptor	F	7.8 +/- 0.46 (7)	-

Footnotes:

[1] n/a = No animals survived beyond postnatal day 40

[2] - = Animals were not monitored past the first 40 postnatal days

[3] For the shRNA experiment, we report mean +/- SEM weight for P16 and not P15. Wet food was provided to these mice on the floor of the cage every day starting on P14.

Source data are provided as a Source Data file.

Supplementary Table 3. Summary of brain anatomy phenotypes in *Tsc1;Rptor;Emx1-Cre* mice by genotype and sex.

Genotype	$Tsc1^{wt/wt};Rptor^{wt/wt};Emx1\text{-}Cre^{+}$		$Tsc1^{fl/fl};Rptor^{wt/wt};Emx1\text{-}Cre^{+}$		$Tsc1^{fl/fl};Rptor^{wt/fl};Emx1\text{-}Cre^{+}$	
**Sex**	**Females (n=4)**	**Males (n=4)**	**Females (n=4)**	**Males (n=4)**	**Females (n=4)**	**Males (n=4)**
	**Mean +/- SEM**		**Mean +/- SEM**		**Mean +/- SEM**	
Cortical thickness (µm)	963.50 +/- 37.62	990.10 +/- 54.72	1176.00 +/- 48.51 ***p=0.0181**[1]	1262.00 +/- 24.62 ***p=0.0181**	1085.00 +/- 14.12 ns, p=0.5094	1105.00 +/- 46.99 ns, p>0.9999
	WT Females vs males ns, p=0.6857[2]		KO Females vs males ns, p=0.2000		KO;Het Females vs males ns, p>0.9999	
CA1 thickness (µm)	56.85 +/- 3.16	56.03 +/- 4.83	83.58 +/- 7.54 ***p=0.0427**	95.01 +/- 5.33 ***p=0.0243**	80.26 +/- 13.22 ns, p=0.2866	69.82 +/- 7.62 ns, p>0.9999
	WT Females vs males ns, p=0.8857		KO Females vs males ns, p=0.2000		KO;Het Females vs males ns, p=0.8857	
DG suprapyramidal blade thickness (µm)	48.20 +/- 2.97	52.09 +/- 4.23	66.25 +/- 3.58 ***p=0.0324**	66.21 +/- 3.57 ns, p=0.1873	52.68 +/- 6.39 ns, p=0.9804	52.29 +/- 7.81 ns, p>0.9999
	WT Females vs males ns, p=0.6857		KO Females vs males ns, p=0.8857		KO;Het Females vs males ns, p=0.8857	
DG infrapyramidal blade thickness (µm)	38.52 +/- 2.19	44.01 +/- 4.47	55.92 +/- 3.88 ***p=0.02243**	66.53 +/- 1.22 ns, p=0.0558	51.90 +/- 2.33 ns, p=0.1184	43.33 +/- 4.45 ns, p>0.9999
	WT Females vs males ns, p=0.4857		KO Females vs males ns, p=0.2000		KO;Het Females vs males ns, p=0.2000	
GFAP intensity across cortical layers (arb.units)	213.30 +/- 24.21	190.80 +/- 18.07	288.60 +/- 28.22 ns, p=0.0997	292.10 +/- 38.61 ***p=0.0285**	300.70 +/- 27.35 ns, p=0.062	236.30 +/- 28.02 ns, p=0.5615
	WT Females vs males ns, p>0.9999		KO Females vs males ns, p=0.8857		KO;Het Females vs males ns, p=0.3429	
GFAP intensity in CA1 (arb.units)	278.90 +/- 53.94	263.10 +/- 23.43	363.40 +/- 32.79 ns, p=0.5094	457.00 +/- 51.27 ***p=0.0243**	339.40 +/- 14.30 ns, p=0.9804	312.20 +/- 28.31 ns, p=0.7179
	WT Females vs males ns, p=0.6857		KO Females vs males ns, p=0.2000		KO;Het Females vs males ns, p=0.6857	
MBP intensity (arb.units)	13340.00 +/- 670.00	13365.00 +/- 988.50	7561.00 +/- 418.40 ****p=0.0098**	7143.00 +/- 319.60 ****p=0.0071**	10059.00 +/- 933.40 ns, p=0.4240	9946.00 +/-790.20

						ns,<br>p=0.5094
	WT Females vs males<br>ns, p=0.8857		KO Females vs males<br>ns, p=0.6857		KO;Het Females vs males<br>ns, p>0.9999	
# of ectopic neurons above CA1	4.25 +/-<br>0.25	3.75 +/-<br>0.85	33.00 +/-<br>6.49<br>*p=0.0230	45.50 +/-<br>4.83<br>**p=0.0051	23.00 +/-<br>9.00<br>ns, p=0.1143	26.00 +/-<br>1.47<br>ns, p=0.35
	WT Females vs males<br>ns, p=0.6286		KO Females vs males<br>ns, p=0.2286		KO;Het Females vs males<br>ns, p=0.4857	
Cortical neurons soma area (µm)	362.30 +/-<br>4.18	361.50 +/-<br>3.90	403.80 +/-<br>4.14<br>****p<0.0001	420.90 +/-<br>3.93<br>****p<0.0001	383.00 +/-<br>3.93<br>****p<0.0001	380.50 +/-<br>3.72<br>****p<0.000<br>1
	WT Females vs males<br>ns, p=0.8042		KO Females vs males<br>****p<0.0001		KO;Het Females vs males<br>ns, p=0.6823	
Cortical neurons p-S6 intensity (arb.units)	107.30 +/-<br>1.19	92.71 +/-<br>0.99	131.40 +/-<br>1.53<br>****p<0.0001	119.80 +/-<br>1.27<br>****p<0.0001	126.80+/-<br>1.57<br>****p<0.0001	97.96 +/-<br>1.37<br>*p=0.0326
	WT Females vs males<br>****p<0.0001		KO Females vs males<br>****p<0.0001		KO;Het Females vs males<br>****p<0.0001	
CA1 neurons soma area (µm)	272.70 +/-<br>4.16	277.70 +/-<br>4.55	409.50 +/-<br>8.24<br>****p<0.0001	409.30 +/-<br>8.32<br>****p<0.0001	285.60 +/-<br>3.99<br>ns, p=0.0857	286.30 +/-<br>3.45<br>ns,<br>p=0.1217
	WT Females vs males<br>ns, p=0.5256		KO Females vs males<br>ns, p=0.8329		KO;Het Females vs males<br>ns, p=0.4096	
CA1 neurons p-S6 intensity (arb.units)	98.38 +/-<br>1.47	101.60 +/-<br>1.30	149.90 +/-<br>2.27<br>****p<0.0001	124.20 +/-<br>2.32<br>****p<0.0001	118.50 +/-<br>1.86<br>****p<0.0001	85.92 +/-<br>1.53<br>****p<0.000<br>1
	WT Females vs males<br>*p=0.0372		KO Females vs males<br>****p<0.0001		KO;Het Females vs males<br>****p<0.0001	
DG neurons soma area (µm)	209.00 +/-<br>3.27	217.60 +/-<br>3.34	233.30 +/-<br>3.56<br>****p<0.0001	259.80 +/-<br>4.57<br>****p<0.0001	232.50 +/-<br>3.24<br>****p<0.0001	216.80 +/-<br>3.39<br>ns,<br>p>0.9999
	WT Females vs males<br>*p=0.0496		KO Females vs males<br>***p=0.0001		KO;Het Females vs males<br>***p=0.0004	
DG neurons p-S6 intensity (arb.units)	96.49 +/-<br>1.72	103.50 +/-<br>1.61	162.20 +/-<br>4.29<br>****p<0.0001	132.00 +/-<br>4.17<br>*p=0.0203	126.40 +/-<br>2.27<br>****p<0.0001	83.65 +/-<br>1.67<br>****p<0.000<br>1
	WT Females vs males<br>**p=0.0044		KO Females vs males<br>****p<0.0001		KO;Het Females vs males<br>****p<0.0001	

Footnotes:

[1] P values displayed in the Mean +/- SEM boxes denote Kruskal-Wallis two-sided Dunn's multiple comparisons tests of the indicated genotype to WT mice of the same sex. P values were corrected for multiple comparisons.

[2] P values displayed below the Mean +/- SEM boxes denote comparisons between female and male mice within each genotype (Mann-Whitney tests, two-sided).

Source data are provided as a Source Data file.

Supplementary Table 4. Mouse strains and genotyping primers.

Mouse Line	Genotyping Primers	Source	Reference
Emx1-Cre	WT F: AAG GTG TGG TTC CAG AAT CG	JAX strain # 005628	Gorski, J.A., et al, J Neurosci (2002)[1]
	WT R: CTC TCC ACC AGA AGG CTG AG		
	Mut F: GCG GTC TGG CAG TAA AAA CTA TC		
	Mut R: GTG AAA CAG CAT TGC TGT CAC TT		
$Tsc1^{fl/fl}$	F: GTC ACG ACC GTA GGA GAA GC	JAX strain # 005680	Kwiatkowski, D.J., et al, Hum Mol Genet (2002)[2]
	R: GAA TCA ACC CCA CAG AGC AT		
$Rptor^{fl/fl}$	F: AGCCTTTAGTACCCACTTGGC	JAX strain # 013188	Sengupta, S., et al, Nature (2010)[3]
	R: GGCATCTCACAAAGGGTACAG		
$Rictor^{fl/fl}$	F: ACTGATATGTTCATGGTTGTG	JAX strain # 020649	Tang, F., et al, J Immunol (2012)[4]; Magee, J.A., et al, Cell Stem Cell (2012)[5]
	R: GACACTGGATTCAGTGGCTTG		
Ai9	WT F: AAG GGA GCT GCA GTG GAG TA	JAX strain # 007909	Madisen, L., et al, Nat Neuro (2010)[6]
	WT R: CCG AAA ATC TGT GGG AAG TC		
	Mut F: CTG TTC CTG TAC GGC ATG G		
	Mut R: GGC ATT AAA GCA GCG TAT CC		

Supplementary Table 5. Viruses and titers.

**Virus**	**Ser**	**Promoter**	**Source**	**Titer of viral stock (vg/ml)**	**Dilution amount (*in vitro*)**	**Dilution amount (*in vivo*)**	**Figure panels**
AAV1.hSyn.HI. eGFP-Cre. WPRE.SV40	1	hSyn	Penn Vector Core	1.78×10^{13}	1:20 0.5 µl/well	n/a	1, 2, 3a-f, S1, S2, S3
AAV1.hSyn.eGFP. WPRE.bGH	1	hSyn	Penn Vector Core	3.86×10^{13}	1:20 0.5 µl/well	n/a	1, 2, 3a-f, S1, S2, S3
AAV5.hSyn. eGFP	5	hSyn	UNC Vector Core	4×10^{12}	1:100 0.5 µl/well	n/a	3h,i, S4
AAV1.CBA. mCherry-Cre. WPRE.bGH	1	CBA	Penn Vector Core	1.04×10^{13}	1:100 0.5 µl/well	n/a	3h,i, S4
AAV9.CAG.Flex. tdTomato. WPRE.bGH AllenInstitute864	9	CAG	Penn Vector Core	unknown	1:20 0.5 µl/well	n/a	3h,i, S4
AAV1.pCAG. FLEX-EGFP-WPRE	1	CAG	Penn Vector Core	unknown	1:20 0.5 µl/well	n/a	S7a
AAV1.Syn.NES-jRGECO1a. WPRE.SV40	1	hSyn	Gift from Adesnik lab	2.08×10^{13}	1:20 0.5 µl/well	n/a	7, S7b-m, S8, S9d,e, S10, S11, S12i,j
AAV9.U6. shRptor-EYFP	9	hU6	Caltech CLOVER Center	1.93×10^{14}	1:20 0.5 µl/well	1:4 500 nl/mouse	8, 9, S12, S13
AAV9.U6. shControl-EYFP	9	hU6	Caltech CLOVER Center	2.17×10^{14}	1:20 0.5 µl/well	1:4 500 nl/mouse	8, 9, S12, S13

Ser=serotype

Supplementary Table 6. Antibodies and dilutions.

	Antibody	Host species	Company and catalog #	WB dilution	IHC dilution	ICC dilution
Primary	Tsc1	Rabbit	Cell Signaling 6935	1:800	-	-
	Raptor	Rabbit	Cell Signaling 2280	1:800	-	-
	Rictor	Rabbit	Cell Signaling 2114	1:600	-	-
	rpS6	Rabbit	Cell Signaling 2217	1:1000	-	-
	phospho-rpS6 Ser244/246	Rabbit	Cell Signaling 5364	1:2000	1:800	-
	Akt	Rabbit	Cell Signaling 4691	1:1500	-	-
	phospho-Akt Ser473	Rabbit	Cell Signaling 4060	1:1000	-	-
	4E-BP1	rabbit	Cell Signaling 9644	1:1000	-	-
	phospho-4E-BP1 Thr37/46	Rabbit	Cell Signaling 2855	1:1000	-	-
	Histone 3	Mouse	Cell Signaling 3638	1:2000	-	-
	GFP	Chicken	AbCam ab13970	-	1:1000	1:5000
	MBP	Rat	Abcam ab7349	-	1:350	-
	GFAP	Rabbit	Fisher 180063	-	1:400	-
	NeuN clone A60	Mouse	Millipore MAB377	-	1:800	-
	**Antibody**	**Species (reactivity)**	**Company and catalog #**	**WB dilution**	**IHC dilution**	**ICC dilution**
Secondary	Goat anti-Rabbit-HRP	Rabbit	Bio-Rad 170-5046	1:5000	-	-
	Goat anti-Mouse-HRP	Mouse	Bio-Rad 170-5047	1:5000	-	-
	Goat anti-Rat Alexa Fluor 488	Rat	Thermo Fisher A-11006	-	1:500	-
	Goat anti-Chicken Alexa Fluor 488	Chicken	Thermo Fisher A-11039	-	1:500	1:500
	Goat anti-Mouse Alexa Fluor 546	Mouse	Thermo Fisher A-11003	-	1:500	-

	Goat anti-Rabbit Alexa Fluor 633	Rabbit	Thermo Fisher A-21070	-	1:500	-

Supplementary References

1. Gorski JA, Talley T, Qiu M, Puelles L, Rubenstein JL, Jones KR. Cortical excitatory neurons and glia, but not GABAergic neurons, are produced in the Emx1-expressing lineage. *J Neurosci* **22**, 6309-6314 (2002).

2. Kwiatkowski DJ*, et al.* A mouse model of TSC1 reveals sex-dependent lethality from liver hemangiomas, and up-regulation of p70S6 kinase activity in Tsc1 null cells. *Hum Mol Genet* **11**, 525-534 (2002).

3. Sengupta S, Peterson TR, Laplante M, Oh S, Sabatini DM. mTORC1 controls fasting-induced ketogenesis and its modulation by ageing. *Nature* **468**, 1100-1104 (2010).

4. Tang F, Wu Q, Ikenoue T, Guan KL, Liu Y, Zheng P. A critical role for Rictor in T lymphopoiesis. *J Immunol* **189**, 1850-1857 (2012).

5. Magee JA, Ikenoue T, Nakada D, Lee JY, Guan KL, Morrison SJ. Temporal changes in PTEN and mTORC2 regulation of hematopoietic stem cell self-renewal and leukemia suppression. *Cell Stem Cell* **11**, 415-428 (2012).

6. Madisen L*, et al.* A robust and high-throughput Cre reporting and characterization system for the whole mouse brain. *Nature neuroscience* **13**, 133-140 (2010).
