# Supplementary material for: Raptor downregulation rescues neuronal phenotypes in mouse models of Tuberous Sclerosis Complex
Source: Nat Commun. 2022 Aug 9;13:4665. doi: 10.1038/s41467-022-31961-6 (PMC9363483; doi:10.1038/s41467-022-31961-6)
Supplement: Supplementary file 2 — Description of Additional Supplementary Files [file 41467_2022_31961_MOESM2_ESM.pdf]

Description of Additional Supplementary Files

File Name: Supplementary Movie 1
Description: **Calcium imaging of a primary hippocampal culture from a *Tsc1$^{wt/wt}$;Rptor$^{wt/wt}$;Emx1-Cre*+ (Tsc1-WT) mouse**. AAV-jRGECO1a was added on DIV 2 and spontaneous calcium transients were imaged on DIV 14. Video shows a 32 second (20 frames per second (fps)) movie recorded at 8.91 Hz frame rate. Scale bar = 250µm.

File Name: Supplementary Movie 2
Description: **Calcium imaging of a primary hippocampal culture from a *Tsc1$^{fl/fl}$;Rptor$^{wt/wt}$;Emx1-Cre*+ (Tsc1-cKO) mouse**. AAV-jRGECO1a was added on DIV 2 and spontaneous calcium transients were imaged on DIV 14. Video shows a 32 second (20 fps) movie recorded at 8.91 Hz frame rate. Scale bar = 250µm.

File Name: Supplementary Movie 3
Description:**. Calcium imaging of a primary hippocampal culture from a *Tsc1$^{fl/fl}$;Rptor$^{wt/fl}$;Emx1-Cre*+ (Tsc1-cKO;Raptor-cHet) mouse**. AAV-jRGECO1a was added on DIV 2 and spontaneous calcium transients were imaged on DIV 14. Video shows a 32 second (20 fps) movie recorded at 8.91 Hz frame rate. Scale bar = 250µm.

File Name: Supplementary Movie 4
Description: **Calcium imaging of a primary hippocampal culture from a *Tsc1$^{wt/wt}$;Rptor$^{wt/wt}$;Emx1-Cre*+ (Tsc1-WT) mouse**. AAV-jRGECO1a was added on DIV 2 and spontaneous calcium transients were imaged on DIV 14. Video shows a 32 second (20 fps) movie recorded at 8.91 Hz frame rate. Scale bar = 250µm.

File Name: Supplementary Movie 5
Description:**. Calcium imaging of a primary hippocampal culture from a *Tsc1$^{wt/fl}$;Rptor$^{wt/wt}$;Emx1-Cre*+ (Tsc1-cHet) mouse**. AAV-jRGECO1a was added on DIV 2 and spontaneous calcium transients were imaged on DIV 14. Video shows a 32 second (20 fps) movie recorded at 8.91 Hz frame rate. Scale bar = 250µm.
