# Supplementary material for: Raptor downregulation rescues neuronal phenotypes in mouse models of Tuberous Sclerosis Complex
Source: Nat Commun. 2022 Aug 9;13:4665. doi: 10.1038/s41467-022-31961-6 (PMC9363483; doi:10.1038/s41467-022-31961-6)
Supplement: Supplementary file 9 — Reporting Summary [file 41467_2022_31961_MOESM9_ESM.pdf]

# nature portfolio

Corresponding author(s): Helen Bateup

Last updated by author(s): June 9, 2022

# Reporting Summary

Nature Portfolio wishes to improve the reproducibility of the work that we publish. This form provides structure for consistency and transparency in reporting. For further information on Nature Portfolio policies, see our Editorial Policies and the Editorial Policy Checklist.

## Statistics

For all statistical analyses, confirm that the following items are present in the figure legend, table legend, main text, or Methods section.

| n/a | Confirmed | |
|---|---|---|
| ☐ | ☒ | The exact sample size ($n$) for each experimental group/condition, given as a discrete number and unit of measurement |
| ☐ | ☒ | A statement on whether measurements were taken from distinct samples or whether the same sample was measured repeatedly |
| ☐ | ☒ | The statistical test(s) used AND whether they are one- or two-sided<br>*Only common tests should be described solely by name; describe more complex techniques in the Methods section.* |
| ☒ | ☐ | A description of all covariates tested |
| ☐ | ☒ | A description of any assumptions or corrections, such as tests of normality and adjustment for multiple comparisons |
| ☐ | ☒ | A full description of the statistical parameters including central tendency (e.g. means) or other basic estimates (e.g. regression coefficient) AND variation (e.g. standard deviation) or associated estimates of uncertainty (e.g. confidence intervals) |
| ☐ | ☒ | For null hypothesis testing, the test statistic (e.g. $F$, $t$, $r$) with confidence intervals, effect sizes, degrees of freedom and $P$ value noted<br>*Give P values as exact values whenever suitable.* |
| ☒ | ☐ | For Bayesian analysis, information on the choice of priors and Markov chain Monte Carlo settings |
| ☒ | ☐ | For hierarchical and complex designs, identification of the appropriate level for tests and full reporting of outcomes |
| ☒ | ☐ | Estimates of effect sizes (e.g. Cohen's $d$, Pearson's $r$), indicating how they were calculated |

*Our web collection on statistics for biologists contains articles on many of the points above.*

## Software and code

Policy information about availability of computer code

| | |
|---|---|
| Data collection | Confocal microscopy images were acquired using Zen 2010 blue edition, FluoView 1000 and FluoView 3000. Calcium imaging data were acquired with Micro-Manager 1.4. Details on data collection for each experiment are provided in the Methods. |
| Data analysis | Confocal images and western blot data were analyzed with ImageJ 1.52p and Microsoft Excel (2016). Calcium imaging data analysis was performed with ImageJ 1.53c and custom programs written in Matlab 2020a (calcium imaging analysis code available at https://github.com/FranklinHolme/spontaneous-activity). Mouse survival and body weight data were analyzed with Microsoft Excel 2016. Graphs and statistics for all data were generated with GraphPad Prism versions 7 or 9. Details on quantification and data analysis for each experiment are provided in the Methods. BioRender application (https://biorender.com/) was used to generate all experimental schematics. |

For manuscripts utilizing custom algorithms or software that are central to the research but not yet described in published literature, software must be made available to editors and reviewers. We strongly encourage code deposition in a community repository (e.g. GitHub). See the Nature Portfolio guidelines for submitting code & software for further information.

## Data

Policy information about availability of data

All manuscripts must include a data availability statement. This statement should provide the following information, where applicable:

- Accession codes, unique identifiers, or web links for publicly available datasets
- A description of any restrictions on data availability
- For clinical datasets or third party data, please ensure that the statement adheres to our policy

The data generated in this study are provided in the Source Data file.

# Field-specific reporting

Please select the one below that is the best fit for your research. If you are not sure, read the appropriate sections before making your selection.

- [x] Life sciences
- [ ] Behavioural & social sciences
- [ ] Ecological, evolutionary & environmental sciences

For a reference copy of the document with all sections, see nature.com/documents/nr-reporting-summary-flat.pdf

# Life sciences study design

All studies must disclose on these points even when the disclosure is negative.

| | |
|---|---|
| Sample size | No sample-size calculations were performed prior to the experiments.<br>For in vitro culture based assays (WB, ICC, Ca2+ imaging) sample sizes were determined to be adequate based on prior literature and the magnitude and consistency of measurable differences between groups. (as in: PMID: 23664616, PMID: 32125271,PMID: 22810227, PMID: 33976205, PMID: 31591157). For survival, body weight measures, and IHC experiments sample size was determined to be adequate based on prior literature and the magnitude and consistency of measurable differences between groups (as in: PMID: 28183733, PMID: 22056141, PMID: 31636454, PMID: 26693177) |
| Data exclusions | One breeding pair was excluded from the survival and body weight study as all pups exhibited poor development regardless of genotype. No other data were excluded. |
| Replication | For all quantifications, the experiments were performed once using biological replicates collected across different batches (i.e. in vitro data were obtained from at least three independent culture preps and in vivo data were obtained from mice from multiple litters). For representative images that were not quantified, we have stated in the figure legends how many times that result was replicated. |
| Randomization | Where applicable, animals and samples were randomly selected to receive particular treatments. For primary culture experiments, culture wells were randomly chosen to receive AAV-Cre or AAV-GFP or rapamycin or vehicle. For intracranial injection of AAV-shRNA, mice were randomly chosen to receive either shRptor or shControl. For all other experiments, animals and samples were allocated into groups based on their genotype. |
| Blinding | Experimenters were blinded to the animals' genotypes for survival and body weight monitoring, histology, imunocytochemistry, western blotting and calcium imaging experiments. AAV-shRNA histology experiments were performed blind to genotype and treatment. Calcium imaging analysis was performed blind to genotype. |

# Reporting for specific materials, systems and methods

We require information from authors about some types of materials, experimental systems and methods used in many studies. Here, indicate whether each material, system or method listed is relevant to your study. If you are not sure if a list item applies to your research, read the appropriate section before selecting a response.

## Materials & experimental systems

| n/a | Involved in the study | |
|---|---|---|
| ☐ | ☒ | Antibodies |
| ☒ | ☐ | Eukaryotic cell lines |
| ☒ | ☐ | Palaeontology and archaeology |
| ☐ | ☒ | Animals and other organisms |
| ☒ | ☐ | Human research participants |
| ☒ | ☐ | Clinical data |
| ☒ | ☐ | Dual use research of concern |

## Methods

| n/a | Involved in the study | |
|---|---|---|
| ☒ | ☐ | ChIP-seq |
| ☒ | ☐ | Flow cytometry |
| ☒ | ☐ | MRI-based neuroimaging |

## Antibodies

| | |
|---|---|
| Antibodies used | Hamartin/TSC1 (D43E2) , Cell Signaling #6935 (WB)<br>Raptor (24C12), Cell Signaling #2280 (WB)<br>Rictor (53A2), Cell Signaling #2114 (WB)<br>S6 Ribosomal Protein (54D2), Cell Signaling #2317 (WB)<br>Phospho-S6 Ribosomal Protein (Ser240/244) (D68F8) XP®, Cell Signaling #5364 (WB, IHC)<br>Akt (pan) (C67E7), Cell Signaling #4691 (WB)<br>Phospho-Akt (Ser473) (D9E) XP®, Cell Signaling #4060 (WB)<br>4E-BP1 (53H11), Cell Signaling #9644 (WB)<br>Phospho-4E-BP1 (Thr37/46) (236B4), Cell Signaling #2855 (WB)<br>Histone H3 (96C10), Cell Signaling #3638 (WB)<br>GFP, Abcam #ab13970 (IHC, ICC) |

MBP, Abcam #ab7349 (IHC)
GFAP, Fisher #180063 (IHC)
NeuN, Millipore #MAB377, Clone A60 (IHC)
Goat anti-Rabbit-HFP, Bio-Rad #170-5046 (WB)
Goat anti-Mouse-HRP, Bio-Rad #170-5047 (WB)
Goat anti-Rat Alexa Fluor 488, Thermo Fisher #A-11006 (IHC)
Goat anti-Chicken Alexa Fluor 488, Thermo Fisher #A-11039 (IHC, ICC)
Goat anti-Mouse Alexa Fluor 546, Thermo Fisher #A-11003 (IHC)
Goat anti-Rabbit Alexa Fluor 633,Thermo Fisher #A-21070 (IHC)

Validation

All antibodies used for this study are commercially available and were validated by the manufacturer for the species and assays they were used for in this study.

Primary antibodies purchased from Cell Signaling have been validated by the manufacturer in multiple research applications (https://www.cellsignal.com/about-us/cst-antibody-performance-guarantee).
Hamartin/TSC1 (D43E2), Cell Signaling #6935. According to CST this Ab recognizes Tsc1 protein in mice by WB (Used for WB)
We have independently verified that no signal is observed in Tsc1 KO samples.

Raptor (24C12), Cell Signaling #2280. According to CST this Ab recognizes Raptor protein in mice by WB (Used for WB)
We have independently verified that signal is lost in Raptor KO samples.

Rictor (53A2), Cell Signaling #2114. According to CST this Ab recognizes Rictor protein in mice by WB (Used for WB)
We have independently verified that no signal is observed in Rictor KO samples.

S6 Ribosomal Protein (54D2), Cell Signaling #2317. According to CST this Ab recognizes S6 protein in mice by WB (Used for WB)
Phospho-S6 Ribosomal Protein (Ser240/244) (D68F8) XP®, Cell Signaling #5364. According to CST this Ab recognizes pS6-S240/244 protein in mice by WB and IHC (Used for WB and IHC)

Akt (pan) (C67E7), Cell Signaling #4691 According to CST this Ab recognizes Akt protein in mice by WB (Used for WB)

Phospho-Akt (Ser473) (D9E) XP®, Cell Signaling #4060. According to CST this Ab recognizes pAkt-S473 protein in mice by WB (Used for WB)

4E-BP1 (53H11), Cell Signaling #9644. According to CST this Ab recognizes 4EBP1 protein in mice by WB (Used for WB)

Phospho-4E-BP1 (Thr37/46) (236B4), Cell Signaling #2855. According to CST this Ab recognizes p4EBP1-T37 protein in mice by WB (Used for WB)

Histone H3 (96C10), Cell Signaling #3638. According to CST this Ab recognizes H3 protein in mice by WB (Used for WB)

Antibodies purchased from Abcam were used for assays covered by the "Abpromise guarantee" (https://www.abcam.com/help/abpromise-guarantee)
GFP, Abcam #ab13970. According to Abcam this Ab is suitable for ICC/IF (Used for IHC, ICC)
We have independently verified that the antibody staining matches GFP expression patterns.

MBP, Abcam #ab7349. According to Abcam this Ab recognizes Myelin Basic Protein in mice by IHC (Used for IHC)

The GFAP, Fisher #180063 has been validated for staining in mouse tissue (https://www.biocompare.com/9776-Antibodies/2117165-Rabbit-AntiGFAP-Antibody/#undefined)
GFAP, Fisher #180063. According to Thermo Fisher Scientific this Ab recognizes GFAP in mouse by IHC (Used for IHC)

The NeuN, Millipore #MAB377, Clone A60 has been published and validated for IF in mouse samples (https://www.emdmillipore.com/US/en/product/Anti-NeuN-Antibody-clone-A60,MM_NF-MAB377) (Used for IHC).

## Animals and other organisms

Policy information about studies involving animals; ARRIVE guidelines recommended for reporting animal research

Laboratory animals

All animal procedures and husbandry were carried out in accordance with protocols approved by the University of California, Berkeley Institutional Animal Care and Use Committee (IACUC, protocol #AUP-2016-04-8684-2). Mice were housed with same sex littermates in groups of 5-6 animals per cage and kept on a regular 12 hr light/dark cycle (lights on at 7am), with ad libitum access to food and water. Room temperature was set to 22 degrees C and humidity was not externally controlled.

Laboratory mice of the following strains were used: Tsc1fl/fl, Rptorfl/fl, Tsc1fl/fl;Rptorfl/fl, Tsc1fl/fl;Rictorfl/fl, Tsc1fl/fl;Rptorfl/fl;Emx1-Cre, Tsc1fl/fl;Rictorfl/fl;Emx1-Cre, Tsc1fl/wt;Emx1-Cre. Males and females were used for all experiments in which sex could be specified. For in vitro experiments, newborn animals were used and the sex was not determined. For IHC experiments, P14-16 animals were used. For brain weight, animals were examined at P14-15 and P40-42. For survival and body weight studies, animals were monitored from birth up to 5-6 months of age.

| | |
|---|---|
| Wild animals | This study did not involve wild animals. |
| Field-collected samples | This study did not involve samples collected from the field. |
| Ethics oversight | All animal procedures and husbandry were carried out in accordance with protocols approved by the University of California, Berkeley Institutional Animal Care and Use Committee (IACUC, protocol #AUP-2016-04-8684-2). |

Note that full information on the approval of the study protocol must also be provided in the manuscript.
